# Dimensions of early life adversity and their associations with functional brain organisation

**DOI:** 10.1162/imag_a_00145

**Published:** 2024-04-26

**Authors:** Maria Vedechkina, Duncan E. Astle, Joni Holmes

**Affiliations:** MRC Cognition and Brain Sciences Unit, University of Cambridge, Cambridge, United Kingdom; Department of Psychiatry, University of Cambridge, Cambridge, United Kingdom; School of Psychology, University of East Anglia, Norwich, United Kingdom

**Keywords:** adversity, childhood, functional connectivity, mental health, network neuroscience

## Abstract

Early life adversity is associated with differences in brain function and an elevated risk for poor mental health. Using data from children aged 10 (N = 5,798) from the Adolescent Brain Cognitive Development (ABCD) cohort, we investigated how adversity relates to functional brain organisation using a network neuroscience approach. We derived four data-driven categories of adversity by fitting a mixed graphical model: household/community instability, physical/sexual abuse, parental neglect, and financial difficulties. Analyses revealed that multiple forms of adversity were associated with reduced clustering and increased assortativity across the entire brain and that these local measures of organisation captured greater adversity-related variance than mesoscale measures like modularity. The most pronounced effects were in the somatosensory and subcortical networks. Financial difficulties showed the strongest and most widespread associations with brain organisation, with evidence of a mediating effect of assortativity on the association between financial difficulties and internalising symptoms. Adding race as a covariate attenuated most brain-adversity relationships, suggesting that experiences of adversity are strongly related to race/ethnicity in the ABCD sample. These results demonstrate that different forms of adversity are associated with both shared and unique variations in functional brain organisation, highlighting its potential significance for explaining individual differences in mental health outcomes following early life adversity.

## 1 Introduction

Early life adversity (ELA), such as poverty or abuse, is associated with a range of poor physical, cognitive, and mental health outcomes that persist across the lifespan (Copeland et al., 2018;Kessler et al., 2010;Slopen et al., 2013). It can have especially lasting consequences when occurring during periods of heightened neural plasticity in childhood and adolescence (Kolb et al., 2013). Experiencing adversity in childhood has been linked to alterations in brain structure and function, including differences in regional morphology (Andersen et al., 2008;Galinowski et al., 2015;Teicher et al., 2012), reductions in grey and white matter volumes (De Bellis & Kuchibhatla, 2006;M. A. Sheridan et al., 2012), and more adult-like patterns of functional connectivity (Callaghan & Tottenham, 2016;Gee et al., 2013). Similar alterations in functional connectivity have also been observed across a range of psychiatric conditions, leading some to argue that these differences may play a role in the onset and maintenance of impactful symptoms (Buckholtz & Meyer-Lindenberg, 2012;Gratton et al., 2020;Javaheripour et al., 2021;Xia et al., 2018). An increased understanding of how ELA shapes the developing brain may provide valuable insights into the possible neural underpinnings of psychiatric vulnerability.

While past studies have predominantly focused on regional brain differences (e.g.,Gellci et al., 2019;Herzberg & Gunnar, 2020;Sripada et al., 2014;Teicher et al., 2016) or resting-state connectivity within specific corticolimbic circuitry (Brieant et al., 2021;DeJoseph et al., 2022), there is growing recognition that individual differences in behaviour and mental health may be better explained by distributed neural dynamics across the brain, rather than by local structural or functional characteristics of specific brain regions or circuits in isolation (Fornito et al., 2015). Of critical interest is recent evidence that ELA may give rise to changes in the topological organisation of the developing brain (Gao, Alcauter, Smith, et al., 2015;Kim et al., 2019;Tooley et al., 2021).

The brain can be understood as a complex network whose properties can be studied using mathematical principles from graph theory by representing it as nodes and connections between regions as edges (*network neuroscience*;Bassett & Sporns, 2017). The organisational properties of the brain change dramatically during the first two decades of life as cortical modules become increasingly specialised with age (Betzel et al., 2014;Fair et al., 2009). This process of modular segregation is believed to support improvements in information processing and higher-order cognitive abilities throughout childhood and adolescence (Baum et al., 2017;Gu et al., 2015;Wig, 2017). The brain may be particularly sensitive to environmental input and vulnerable to insult during this period of heightened topological reorganisation, which coincides with changes in myelination and synaptic pruning (Cao et al., 2017;Di Martino et al., 2014;Fornito et al., 2015).

A recent review based on four studies (Tooley et al., 2021) found that children growing up in low socio-economic (SES) households exhibit both higher (Tooley et al., 2020) and lower (Gao, Alcauter, Elton, et al., 2015;Gellci et al., 2019;Sripada et al., 2014;Tooley et al., 2020) levels of segregation at different developmental timepoints. The authors posit that children growing up in low-SES environments may follow a shifted developmental trajectory characterised by earlier increases in age-typical segregation, whereas their higher-SES counterparts follow a later but steeper maturational curve, eventually surpassing their lower SES peers and showing attenuated declines in network segregation across adulthood (Tooley et al., 2021; see alsoChan et al., 2018). Notably, in examining the maturation of functional brain networks in infancy,Gao et al. (2015)found that neonates aged 0–1 from high SES households exhibited faster network maturation (i.e., more rapid increases in segregation), particularly in the sensorimotor and default-mode networks. In contrast,Tooley et al. (2020,^2021^) found that high SES youth had stronger associations between age and segregation two cross-sectional samples aged 4–10 and 8–22, indicative of a later but steeper maturational curve. There is evidence to support that high SES individuals continue to exhibit greater functional segregation into middle age (Chan et al., 2018), although the limited and cross-sectional nature of these studies precludes any conclusions about developmental trajectories.

Existing work applying a graph-theoretical approach to understanding the impact of early adversity on brain development has mainly focused on poverty (Gao, Alcauter, Elton, et al., 2015;Tooley et al., 2020). It is unclear whether differences in connectome organisation that have been linked to low SES and neighbourhood poverty extend to a wider range of adverse exposures, such as abuse, neighbourhood violence, household instability, parental neglect, and trauma (Belsky & De Haan, 2011;Y. Chen & Baram, 2016;McLaughlin & Sheridan, 2016). This is important to know because poverty and maltreatment may have unique associations with neural topology (Herzberg & Gunnar, 2020;McLaughlin & Sheridan, 2016). Using measures of poverty alone likely fails to capture important variation in the types of experiences children growing up in adverse environments are likely to encounter (Berman et al., 2022).

To address this, in the current study, we investigate how a wide range of adversities—including physical and sexual abuse, domestic and community violence, caregiver neglect, and deprivation—relate to functional brain organisation in young people. We aim to discern whether associations between adversity and brain organisation are somewhat specific to different forms of adversity, or whether they exhibit a general effect. This study contributes to the literature by examining adversity-related variation in functional brain organisation within a large racially and economically diverse sample of youth, allowing us to capture demographic differences in adverse exposures and their relationship to the developing brain. We extend existing work on adversity-driven differences in functional connectivity by leveraging a connectomics framework to identify differences in the*organisational*properties of the entire brain.

Deciding how to measure and categorise adversity is challenging (Berman et al., 2022;Smith & Pollak, 2020). Existing frameworks have been instrumental in demonstrating that adverse experiences can have a substantial impact on development. In the “cumulative risk” approach (Evans et al., 2013), each adverse experience is assumed to increase risk for poor outcomes in a homogeneous and additive way. In contrast, the “threat/deprivation” model (McLaughlin & Sheridan, 2016) categorises exposures into two broad theory-based dimensions, recognising that different dimensions of exposure may have unique implications for neurodevelopment. Relying on theory-driven categorisations, while easier to interpret, may also miss relationships that exist across supposed categories (Bignardi et al., 2022). To overcome this challenge, we perform two complementary data-driven analyses to explore how different forms of adversity relate to connectome differences.

First, we fit mixed graphical models to obtain data-driven categories of adversity and assess their unique associations with three measures of network topology (clustering coefficient, modularity, and assortativity) using generalised linear models (GLM). However, just because the adversities themselves fall into categories, this does not mean that these categories are mirrored in their associations with brain organisation. Given the possibility that data-driven adversity categories may not follow a 1:1 mapping with measures of brain organisation, we conduct a second analysis using partial least squares regression (PLS) to*simultaneously*model the co-variance between individual adversity items and measures of network topology. PLS is particularly well-suited to high-dimensional and multicollinear data and may overcome issues that emerge from statistically controlling for different categories of adversity in the same GLM model. Our research, therefore, extends existing frameworks of adversity by simultaneously examining both shared and non-additive, unique effects across different adversities as pertaining to functional brain organisation, recognising the high rates of co-occurrence across proposed dimensions and overlap in their reported neurobiological substrates (Herzog & Schmahl, 2018;Kessler et al., 1997;McLaughlin et al., 2021).

## 2 Methods

### 2.1 Participants

The Adolescent Brain Cognitive Development (ABCD) Study is a longitudinal study that involves 21 data acquisition sites across the US that follows over 11,000 children aged 9–10 for 10 years into early adulthood (Garavan et al., 2018). The study was designed to approximate the socio-demographic distribution of US children in this age group (Table S1). Participants were required to be of the desired age range (9–10 years at baseline) for inclusion in the ABCD study. Those who lacked English language proficiency; suffered from severe sensory, intellectual, medical, or neurological issues; or were unable to participate in MRI scanning were excluded. Parents of participants were required to have either English or Spanish proficiency. Recruitment details and data-collection procedures are described inGaravan et al. (2018). After removing participants with missing data (described below), the final sample for analyses consisted of 5,798 children (Table 1).

**Table 1.** Sample demographics and comparison with excluded participants.

Variable	Included (n = 5,798)	Excluded (n = 5,769)	Statistic	*p*
Age (months)	120 (7.5)	118 (7.4)	-11.28	<.001
Sex (f)	51.43%	43.92%	65.33	<.001
College ed. parent (yes)	63.99%	55.16%	96.36	<.001
Race/ethnicity			205.99	<.001
*White*	58%	47%		
*African american*	12%	18%		
*Asian*	2%	3%		
*Hispanic*	3%	5%		
*Multiracial*	25%	27%		
*Other*	1%	1%		
Binary adversity (yes)	35%	65%	73.88	<.001
Cumulative adversity	4.20 (1.99)	4.57 (2.23)	9.38	<.001
Household & Community Instability	0.96 (1.13)	1.11 (1.25)	6.62	<.001
Physical & Sexual Abuse	0.04 (0.29)	0.06 (0.39)	2.38	0.017
Parental Neglect	2.78 (0.66)	2.87 (0.73)	7.05	<.001
Financial Difficulties	0.41 (1.04)	0.53 (1.16)	5.67	<.001
Internalising symptoms	48.10 (10.53)	49.80 (10.74)	3.55	<.001
Externalising symptoms	45.10 (10.03)	46.35 (10.60)	6.60	<.001

*Notes*. Welch’s t-test used for continuous variables. Chi-square test used for categorical variables. Internalising and externalising symptoms represent t-scored values (adjusted for age and sex). College education reported if one or more caregivers have a college-level degree.

### 2.2 Mental health assessment

The parent-reported Child Behaviour Checklist (CBCL) was used to measure children’s mental health. The CBCL is made up of 113 items rated on a three-point scale (not true; somewhat or sometimes true; very often or always true;Achenbach, 2011). In this study, we used the t-scores from the Internalising and Externalising composite scales, which are obtained by summing the individual items from each domain. Scores are normed by age and gender, thus offering a normalised metric that facilitates comparison across studies (Barch et al., 2021). The scales have good inter-interviewer and test-retest reliability (Achenbach & Rescorla, 2001), and the distribution of t-scores in our sample fits a standard distribution (Table S3). As an additional check, we repeated our mediation analyses (described below) using the raw score values instead of the t-scored values and found that our results were qualitatively unchanged (Table S38). Measures for this study were taken at baseline when participants were aged 9–10 years and three years later at ages 12–13.

### 2.3 Measuring early life adversity

ABCD measures include 31 questions that capture different domains of adversity, including physical abuse, sexual abuse, domestic violence, community violence, caregiver neglect, material deprivation, and traumatic events. All adversity questions are listed inTable S2. Questions were designed to capture experiences from birth up to the baseline assessment, except for the material deprivation questions that ask about experiences in the past 12 months (e.g., “In the past 12 months, has there been a time when…”). Caregiver reports were used due to the age of the participants. A total of 28 questions provided binary scores of whether a child had experienced a given adversity. The remaining three questions were Likert-type scales, which were reverse-coded such that higher values indicated greater adversity. Participants missing more than 15% of data on adversity measures were removed from the analysis (n = 314), and the remaining missing answers were coded as “0” (i.e., adversity not endorsed). These responses were coded as 0 because sensitivity analyses (reported inSupplement 1.2) revealed that either coding the missing responses as 1 (endorsing adversity) or using imputation resulted in estimates of adversity that were substantially higher than population prevalence estimates (Finkelhor et al., 2005;McLaughlin et al., 2012;Struck et al., 2020), indicating both approaches were heavily biased. Moreover, imputation was not appropriate for the adversity data as it was both non-binary and not missing at random (seeSupplementfor details).

### 2.4 Obtaining categories of adversity

To obtain data-driven categories of adversity, mixed graphical models were fit to the adversity measures using the*mgm*package in R (Haslbeck et al., 2019). This method graphically estimates relationships between mixed-type data using nodewise regression to produce a network. Only pairwise interactions were included in the model. To reduce overfitting, networks were regularised using least absolute shrinkage and selection operator (LASSO;Tibshirani, 1996). Lambda (λ), the tuning parameter that controls the strength of the penalization applied to weak edges, was set using the extended Bayesian information criterion which performs well in selecting sparse graphs (EBIC;Foygel & Drton, 2010). The gamma (γ) hyperparameter was set to the recommended value of 0.25 (Hevey, 2018). Edges were retained if both estimates of pairwise interactions (i.e., one from regressing A on B and one from B on A) were nonzero. Adversity networks were visualised using the*qgraph*package in R (Epskamp et al., 2012).

To delineate categories of adversity, we performed community detection on the adversity network using a Walktrap algorithm, implemented using the*igraph*package (Csárdi & Nepusz, 2006). The algorithm captures the community structure of a network by measuring the similarity between its edges based on random walks around the network (Pons & Latapy, 2006). Nodes are iteratively merged into different communities before an optimal partition is selected to maximise the network’s modularity. We evaluated the quality of the resulting partition using the modularity index*Q*, calculated as the number of edges falling within communities minus the number that would be expected by chance (Newman, 2004), Moderate modularity was defined as*Q *> 0.3, and good modularity was defined as*Q*> 0.5. Centrality measures for individual nodes were calculated using the*qgraph*(Epskamp et al., 2012) and*networktools*packages (Jones, 2020). Item response scores within each community were summed to obtain adversity scores for each category (i.e., network community) of adversity. In supplementary analyses, we also calculated a cumulative adversity score by summing all individual adversity items, and a binary adversity categorisation was derived using a median split of high- and low-adversity groups (Table S3).

### 2.5 Imaging acquisition and preprocessing

ABCD standard imaging protocols for resting-state functional MRI (rsfMRI) including acquisition, processing, and quality assurance procedures have been described in detail elsewhere (Casey et al., 2018;Hagler et al., 2019). Briefly, the preprocessing pipeline includes within- and between-scan head-motion correction, distortion corrections, removal of initial frames, normalisation, demeaning, regression, and temporal filtering (Holland et al., 2010;Jovicich et al., 2006). Average time courses for each region of interest (ROI) were calculated using FreeSurfer’s automated brain segmentation (aseg) and resampled to align with voxels from the fMRI data. Motion time courses are adjusted to account for signals linked to respiration (Hagler et al., 2019). This study uses imaging data from a subset of participants with 10 minutes of valid rsfMRI data below a framewise displacement threshold of 0.2 mm collected at the baseline assessment (n = 5,995).

### 2.6 Functional connectome construction

Using the processed rsfMRI data, parcellated time series were computed using a seed-based correlational approach (Van Dijk et al., 2010). Regions of interest (ROIs) were defined using the functional Gordon atlas template which comprises 352 ROIs (333 cortical) belonging to one of 13 networks (12 cortical and 1 subcortical;Gordon et al., 2016). The functional connectivity between any two ROIs was estimated by calculating the lag-zero Pearson correlation coefficient of parcellated time-series. This produced an ROI x ROI correlation matrix for each participant, which underwent an additional variance stabilization procedure using a Fischer z-transform (Feczko et al., 2020).

We represented this signed, undirected matrix as a graph in which ROIs represented network nodes and the functional connectivity between any given pair of ROIs represented the weight of the network edge between those two nodes. Traditionally, most graph measures required edge weights to fall between 0 and 1, which was accomplished either by using absolute values, setting negative weights to zero, or applying other arbitrary thresholds (Rubinov & Sporns, 2010). However, growing evidence suggests that dense weighted networks are more suited than sparse networks for adequately capturing the complexity of neurobiological systems. Further, in the case of functional connectivity, negative edges likely contain valuable and biologically meaningful information (Bassett & Bullmore, 2017;Rubinov & Sporns, 2011). Finally, different thresholds can generate different topological properties in the same network, making it difficult to compare across studies and resulting in poor reliability. The use of fully weighted networks may counteract some of these limitations, while also diminishing the influence of connectivity strength on network organisation (Rubinov & Sporns, 2011;van den Heuvel et al., 2017). In this study, we, therefore, chose to maintain all edge weights, including negative connections, and restricted our analyses to graph measures that can handle negative edges while remaining neurobiologically interpretable (Rubinov & Sporns, 2010).

### 2.7 Extracting connectome statistics

We evaluated the topological properties of functional connectomes by quantifying several graph theoretical measures, implemented with the Brain Connectivity Toolbox in Matlab (BCT;Sporns & Rubinov, 2019). For each participant’s network, the following graph properties were computed at the global level (i.e., across the entire brain): clustering coefficient (C), modularity (Q), and assortativity (A). We also computed the clustering and assortativity measure separately for each Gordon network (12 cortical, 1 subcortical;Gordon et al., 2016).

The clustering coefficient (C), a measure of local segregation, is defined as the ratio of the number of edges around a node to the maximum number of possible edges (Rubinov & Sporns, 2010). This represents the extent to which closely and densely connected nodes form clusters in the connectome. We used Zhang and Horvath’s formula to calculate the clustering coefficient (Zhang & Horvath, 2005). This formulation reduces the sensitivity of the measure to weights directly connected to the node of interest in networks with both positive and negative weights. We obtained a single clustering coefficient value for the entire connectome by averaging the clustering coefficient across all network nodes.

Modularity (Q), a mesoscale measure of segregation, quantifies the degree to which a network can be divided into nonoverlapping communities of nodes in a way that maximises the number of edges within a community and minimises the number of edges between communities (Rubinov & Sporns, 2011). We used a Louvain community detection algorithm to define the global network community structure and obtain a modularity index for the entire signed and weighted functional connectome (Blondel et al., 2008).

Assortativity (A) measures the tendency of nodes in a network to be connected to other nodes with a similar degree and is thought to represent the extent to which a network can resist failure in its main components (Farahani et al., 2019). This is a relatively understudied property in network neuroscience that may provide information about network robustness and resilience to pathological spread (Betzel et al., 2019;Grayson & Fair, 2017). In assortative networks, highly connected nodes tend to be connected to other highly connected nodes, resulting in a network that is robust to random node failures by allowing hubs to take on each other’s activity. However, damage to high-degree nodes in assortative networks can also lead to rapid network degeneration by facilitating the spread of network failure. In contrast, in non-assortative networks, high-degree nodes tend to be connected to low-degree nodes. This results in a network that is less resilient to targeted perturbation due to the presence of vulnerable hubs (Newman, 2002). Despite evidence of its functional importance in biological and non-biological networks, assortativity remains a relatively understudied property in network neuroscience (Betzel et al., 2018;F. Luo et al., 2009). We usedThedchanamoorthy and colleagues’ (2014)formula as a measure of nodal assortativity. A single assortativity value was obtained for the entire functional connectome by summing the local assortativity across all network nodes.

### 2.8 Statistical analysis

#### 2.8.1 Generalised linear models

After testing and ruling out the possibility of any non-linear relationships (Supplement 1.3), generalised linear models (GLM) were performed to examine the association between categories of adversity and clustering, modularity, and assortativity computed at the global level (i.e., across the entire brain). GLM models controlled for age, sex, scanner head motion, and scanner type, as these characteristics can be associated with the topological organisation of functional connectomes and may in turn vary with adversity (A. A. Chen et al., 2022;Gu et al., 2015;Satterthwaite et al., 2012). We did not control for site-level clustering models due to excessive multicollinearity with scanner type. Scanner differences have been shown to introduce significant variability and bias in imaging results (i.e., scanner effects) which can impact functional connectome metrics (A. A. Chen et al., 2022). We therefore chose to maintain scanner type, over site-level clustering, in our models. Unstandardised regression coefficients were reported to maintain interpretability and enable comparison of regression weights between the primary predictors (i.e., adversity measures).

In the Supplement, we present analyses that additionally control for parental education, and race/ethnicity. All data-driven categories of adversity were included in the same model to capture their unique associations with network topology. We calculated the variance inflation factor (VIF) and tolerance for each predictor variable in the model to ensure that the degree of multicollinearity was within the acceptable range. Post-hoc analyses were conducted to check whether the best-fit link function for the model was Gaussian, logarithmic, or inverse. For any significant GLM results, we tested for potential mediating effects of global connectome properties on the relationship between adversity and mental health, both concurrently and three years later. One thousand bootstrap samples were used to calculate 95% confidence intervals.

We pursued several sensitivity analyses (reported inSupplement 2) to further test the utility of data-driven adversity categories and to test whether our findings were sensitive to our chosen method. First, to test the utility of using data-driven categories of adversity, we ran two supplementary GLMs, one with a cumulative adversity score, and another with a binary adversity categorisation (i.e., high vs. low) instead of the data-driven adversity categories. Second, to test whether the significant effects were driven by differences in topology, rather than connection strength only, empirical network measures were compared to those of randomised null networks with preserved weight, degree, and strength distributions (Rubinov & Sporns, 2011). We randomised the functional connectome for each participant by rewiring the edges 10,000 times. We then recalculated global network measures for the randomised networks and compared these to the empirical measures for each participant. We repeated our GLM analyses using the randomised measures to assess the extent to which connectivity strength, rather than topology, was driving results. Non-significant, or less significant, randomised network measures would indicate that our findings reflect true topological differences.

Third, to examine the extent to which our findings were driven by weaker connections, we applied five different edge thresholds—one absolute threshold retaining only positive weights and four proportional thresholds retaining the top 10%, 20%, 30%, and 40% of strongest edges for individual networks—and constructed five corresponding connectomes for each individual retaining only the connections above a given threshold. We then recalculated global network measures for each of the thresholded connectomes and repeated the GLMs using these values. For these analyses, we were restricted to a formulation of assortativity calculated at the global level (Sporns & Rubinov, 2019), which is suitable for networks without any negative weights (Newman, 2002) but which differs fromThedchanamoorthy and colleagues’ (2014)formula of nodal assortativity.

Next, to investigate whether adversity-related alterations vary across functional systems, we performed another set of GLMs using the clustering coefficient and assortativity measures computed at each Gordon network (12 cortical and 1 subcortical;Gordon et al., 2016). All analyses performed at the network level were corrected for the number of networks using false-discovery rate (FDR;*q*< 0.05;Benjamini & Hochberg, 1995). Analyses were performed in Matlab using functions from the*BCT*(Rubinov & Sporns, 2010) and in R using the*stats*(R Core Team, 2021) and*emmeans*(Searle et al., 2022) packages.

#### 2.8.2 Partial least squares

We used partial least squares (PLS) regression as a complementary method to identify individual adversity items that best explain variability in brain network topology. PLS is a data-reduction technique ideally suited to capturing covariance and explaining complex relationships between a large set of noisy and multicollinear variables (e.g., adversity items and brain measures). Instead of deriving latent categories of adversity*before*assessing their relationship with brain network topology, PLS models the relationship between linear combinations of predictor items and linear combinations of outcome variables*simultaneously*by projecting them to a new space. In other words, it models the covariance structure between the X and Y matrices and identifies a set of orthogonal latent variables that best explain this relationship (Wold et al., 2001). By doing so, PLS may help counteract issues of construct validity that may emerge from statistically controlling for frequently co-occurring types of adversity in the same model and may capture the shared and unique variance between adversity and functional connectome organisation more accurately than traditional latent variable and linear regression techniques.

We ran two separate PLS models. The 24 adversity items used in the adversity categories were included as predictors in both. The three global network measures (clustering coefficient, modularity, assortativity) were used as outcomes in the*global*PLS model, whereas the clustering coefficient and assortativity measure at each Gordon network (n = 13) were used as outcomes in the*regional*PLS model. The*p*corr significance values for the PLS components were obtained by permuting the data 10,000 times and comparing the observed coefficients relative to their null distributions. Variable importance in projection (VIP) was used to assess the relative importance of each adversity item to the model, with scores above 1 considered most influential in terms of their explanatory power. Additionally, we applied the Jack-Knife approach, which cross-validates the model to generate a regression distribution, to obtain regression coefficients, confidence intervals, and*p*-values for each adversity item loading (Kucheryavskiy, 2020). The stability of item loadings was assessed by averaging the mean squared error of prediction, R^2^, and Q^2^across 10 cross-validation runs (nrepeat = 10, folds = 10), with a significance threshold of 0.01 for improvement in component error rate. After regressing the predictor and response scores obtained from each PLS model, age, sex, scanner head motion, and scanner type were added as covariates. In the Supplement, we present analyses that additionally control for parental education, and race/ethnicity. For the global PLS results, we tested for potential mediating effects of the component response scores (i.e., latent scores based on connectome measures) on the relationship between the component predictor scores (i.e., latent scores based on adversity items) and mental health, both concurrently and three years later. One thousand bootstrap samples were used to estimate the 95% confidence intervals for indirect effects using the bias-corrected percentile method proposed byBiesanz and colleagues (2010). Analyses were performed in R using the*MixOmics*(Rohart et al., 2017),*caret*(Kuhn, 2022), and*mdatools*(Kucheryavskiy, 2020).

## 3 Results

### 3.1 Descriptive statistics

After removing participants with over 15% missing adversity data (n = 314) and those with less than 10 minutes of valid rsfMRI data (n = 5881), the final study sample consisted of 5,798 participants. The exclusion of participants due to missing data and excessive head motion during scanning resulted in a final sample with the demographic characteristics shown inTable 1. Participants with greater head motion, which necessitated their exclusion, also reported higher levels of adversity and had a different racial/ethnic composition, notably with a greater proportion of African American children. Spearman correlations between adversity categories, global connectome measures, and mental health are shown inFigure 1.

**Fig. 1.**
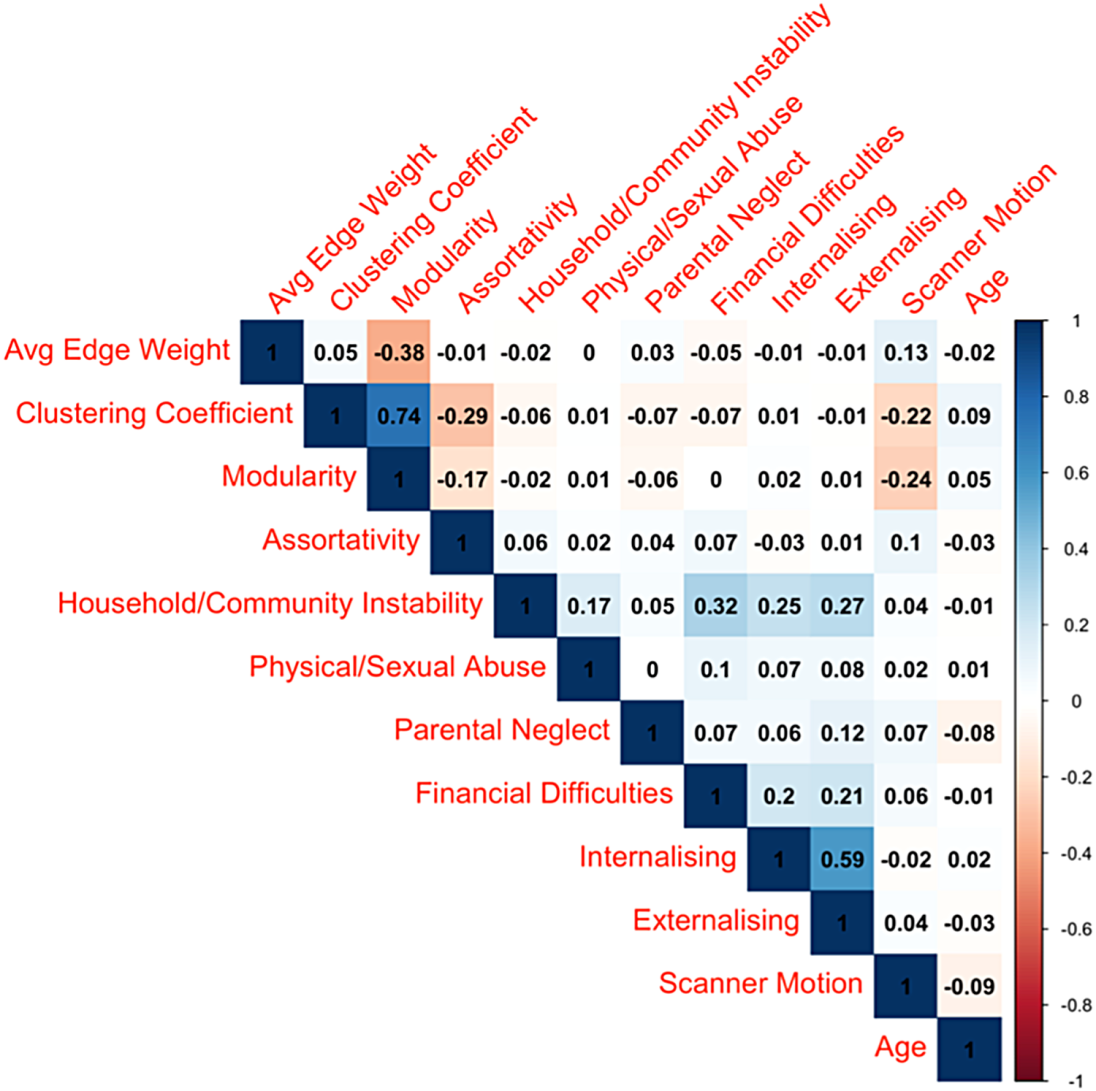
Correlations between study variables.*Notes*. Spearman correlations between adversity categories, global connectome measures, and mental health scales.

We conducted a set of ANOVAs to compare adversity levels by race/ethnicity and parental education (Tables S4-S5). Race/ethnicity and parental education were both significantly associated with adversity in the ABCD sample (Figs S1–S2). The confounding effect of race, education and adversity in our sample therefore posed a statistical difficulty for addressing the question of whether differences in adversity would persist in individuals who differ on race/ethnicity or parental education if they had not differed on these demographic characteristics. Put simply, statistically controlling for pre-existing group differences that are inherently related to adversity would likely remove some or all adversity-related variance, thereby producing biased estimates (*Lord's Paradox*;G. A. Miller & Chapman, 2001). We therefore do not control for race/ethnicity and parental education in our main analyses but provide a supplementary set of results where they are added as additional covariates for comparison (Supplement 2.3).

### 3.2 Obtaining categories of adversity

The adversity network, obtained by fitting mixed graphical models on all adversity items, is shown inFigure 2. Seven of the 31 measures of adversity were removed from the final adversity network as they did not cluster with any other nodes in the network. These consisted of 6 items measuring one-time traumatic events and one broad measure of family conflict (Table S2).

**Fig. 2.**
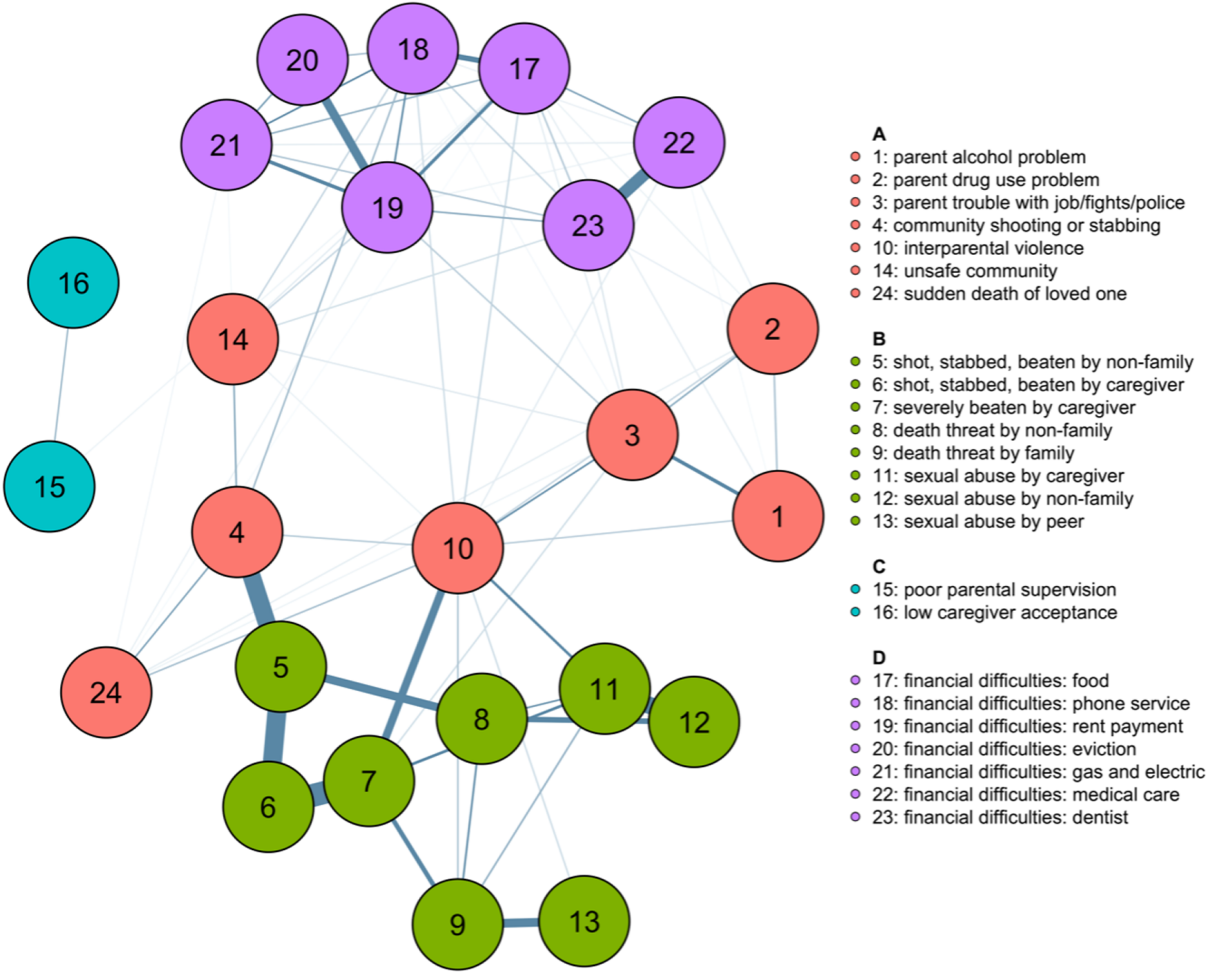
Adversity network.*Notes*. Adversity categories were obtained using mixed graphical models with nodewise regression as an estimate of the association between individual adversity items. The network was regularised using least absolute shrinkage and selection operator (LASSO). Optimal network partition selected based on modularity (*Q*). The colour of nodes denotes categorical membership, and edge thickness represents its strength. Four categories of adversity were identified and were labelled based on the construct that best represented their items: A = household/community instability; B = physical/sexual abuse; C = parental neglect; D = financial difficulties.

Community detection on the adversity network identified four categories of adversity. The first consisted of events relating to instability and lack of safety in the child’s household or community. The second represented experiences of sexual or physical abuse. The third consisted of items measuring parental neglect, and the fourth of items measuring financial difficulties. For ease of reporting, we henceforth refer to these adversity categories as (1) household/community instability; (2) physical/sexual abuse; (3) parental neglect; and (4) financial difficulties. The modularity index for the clustering solution was moderate (*Q*= 0.44). The strongest nodes in the network were items representing physical abuse to the child, followed by interparental violence and financial difficulties (Table S6,Fig. S3). Interparental violence and community shooting/stabbing items had the highest bridge strength, indicating their strong connection to the physical and sexual abuse cluster.

### 3.3 Generalised linear model

#### 3.3.1 Adversity and global connectome topology

**Fig. 3.**
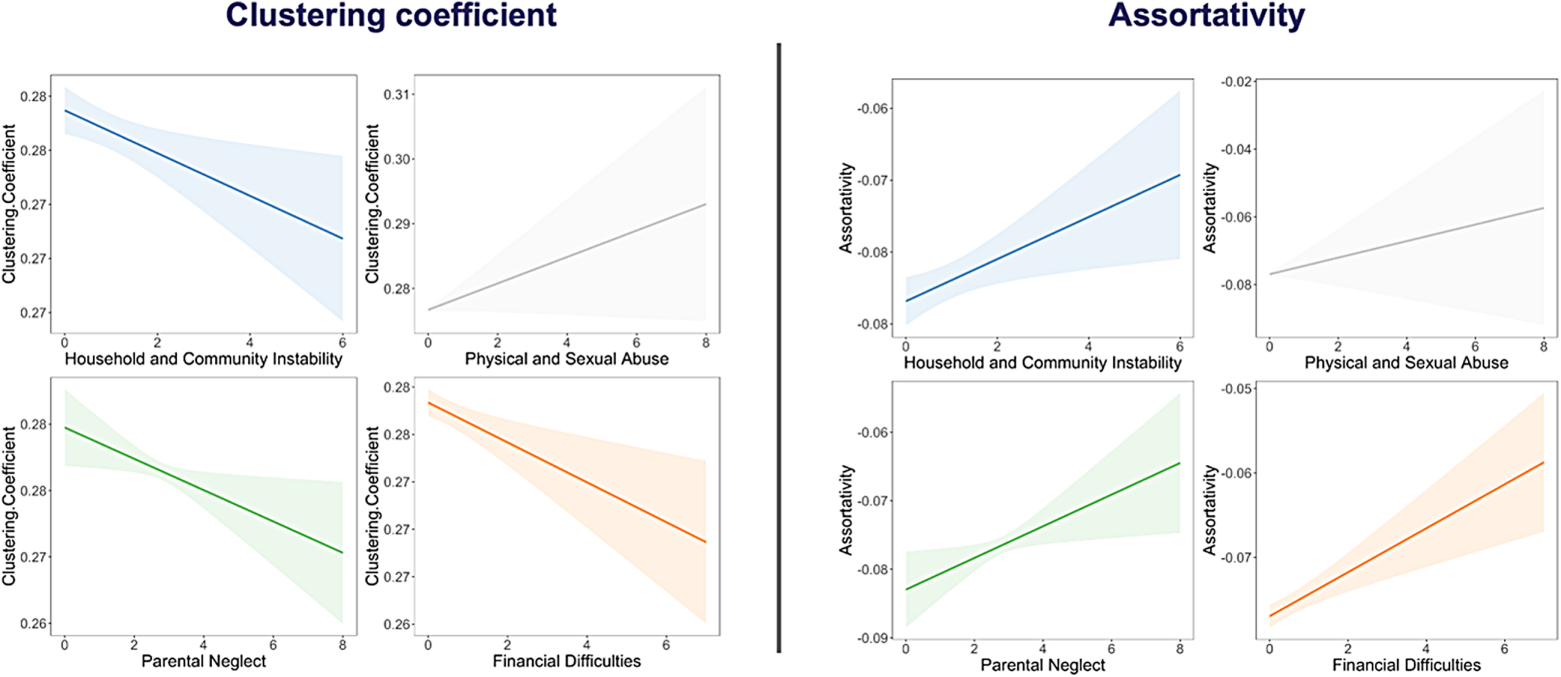
Association between categories of adversity and network measures.*Notes.*GLM with age, sex, scanner head motion, and scanner type were added as covariates. Age reported in years. Scanner type uses GE as the reference category. Correlation shown for Physical and Sexual Abuse is non-significant.

We conducted a set of GLM analyses to determine whether categories of adversity were associated with variation in global clustering coefficient, modularity, and assortativity. Age, sex, scanner head motion, scanner type, and all four categories of adversity were included in the same model as covariates. Greater household/community instability (*t*= -2.43,*p*= .015), parental neglect (*t*= -2.25,*p*= .026), and financial difficulties (*t*= -3.01,*p*= .003) were associated with less global clustering and greater assortativity (Table S7;Fig. 3). There were no significant associations between physical/sexual abuse and any of the global connectome measures. There were no significant associations between adversity and global modularity. There were no significant interaction effects of age or gender. Supplementary analyses revealed that most associations between adversity and global connectome measures disappeared after controlling for race/ethnicity and parental education (Supplement 2.3.1). To test the utility of using data-driven categorisations of adversity, we ran two supplementary GLMs using (1) a cumulative adversity score and (2) a binary split of high- and low-adversity groups instead of the four data-driven categories of adversity. The results mirrored our categorical findings, with significant associations of cumulative and binary adversity scores with clustering and assortativity, but not with modularity (Tables S18–S19).

#### 3.3.2 Mediating effects of global connectome measures on mental health

We tested for possible mediating effects of global connectome measures on the relationship between adversity and mental health. Household/community instability, parental neglect, and financial difficulties were directly associated with greater concurrent internalising (*β’s*= 0.64-1.92,*p’s*= .003-.000;Table S8) and externalising difficulties (*β’s*= 1.24-2.01,*p’s*< .001;Table S9) controlling for age, sex, scanner motion, and scanner type. The only indirect effect was that of assortativity mediating the association between financial difficulties and concurrent internalising difficulties (*β*= -0.02,*p*= .017;Tables S10–S11). Mediation analyses using the cumulative adversity score, instead of data-driven categories, revealed a direct association with concurrent internalising and externalising difficulties, as well as mediating effects of clustering and assortativity (Tables S12–S13). The direct and indirect effects were stronger than those observed in financial difficulties alone, indicating that while differences in brain organisation that mediate mental ill-health most strongly relate to financial difficulties (above other adverse categories), using a cumulative risk score can increase the statistical power by leveraging shared variance across different adverse experiences.

After controlling for baseline symptoms, household and community instability directly predicted greater internalising difficulties 3 years later (*β*= 0.702,*p*< .001;Table S14), whereas household and community instability and parental neglect directly predicted greater externalising difficulties 3 years later (*β’s*= 0.43-0.51,*p*’s = .035-.000;Table S15). There were no significant mediating effects of global connectome measures on later mental health (Tables S16-S17).

#### 3.3.3 Sensitivity analyses

Supplementary sensitivity analyses revealed that additionally controlling for race/ethnicity and parental education removed most significant associations due to their covariance with adversity in our sample (Supplement 2.3.1). We further tested the significance of our main results by comparing them to those obtained from randomised networks with preserved weight, degree, and strength distributions (Table S20). The connectome measures obtained for the empirical networks were significantly different from those expected by chance (*p*’s < .001). Parental neglect was associated with clustering in the randomised network (*β*= -.001,*p=*.003), while financial difficulties were associated with both clustering (*β*= -.001,*p*= .001) and assortativity (*β*= .001,*p*< .001), suggesting that differences in network strength were contributing, in part, to the respective associations between adversity and connectome topology.

To examine the extent to which our results were driven by weaker connections, we repeated our analyses using five different thresholds that differed in their level of stringency. Results for the clustering coefficient were qualitatively similar across all thresholds, with slightly weaker effects with increasing stringency (Tables S21–S25). Results for assortativity were qualitatively similar for household/community instability and parental neglect, with slightly stronger effects with increasing stringency. However, the association between financial difficulties and assortativity did not hold when connectomes were thresholded, suggesting that negative connections were necessary to establish the effect. Follow-up analyses found that only financial difficulties were associated with differences in average edge weight, controlling for age, sex, scanner motion, and scanner type (*β*= -0.0003,*p*< .001;Table S26).

#### 3.3.4 Adversity and regional (network-level) topology

**Fig. 4.**
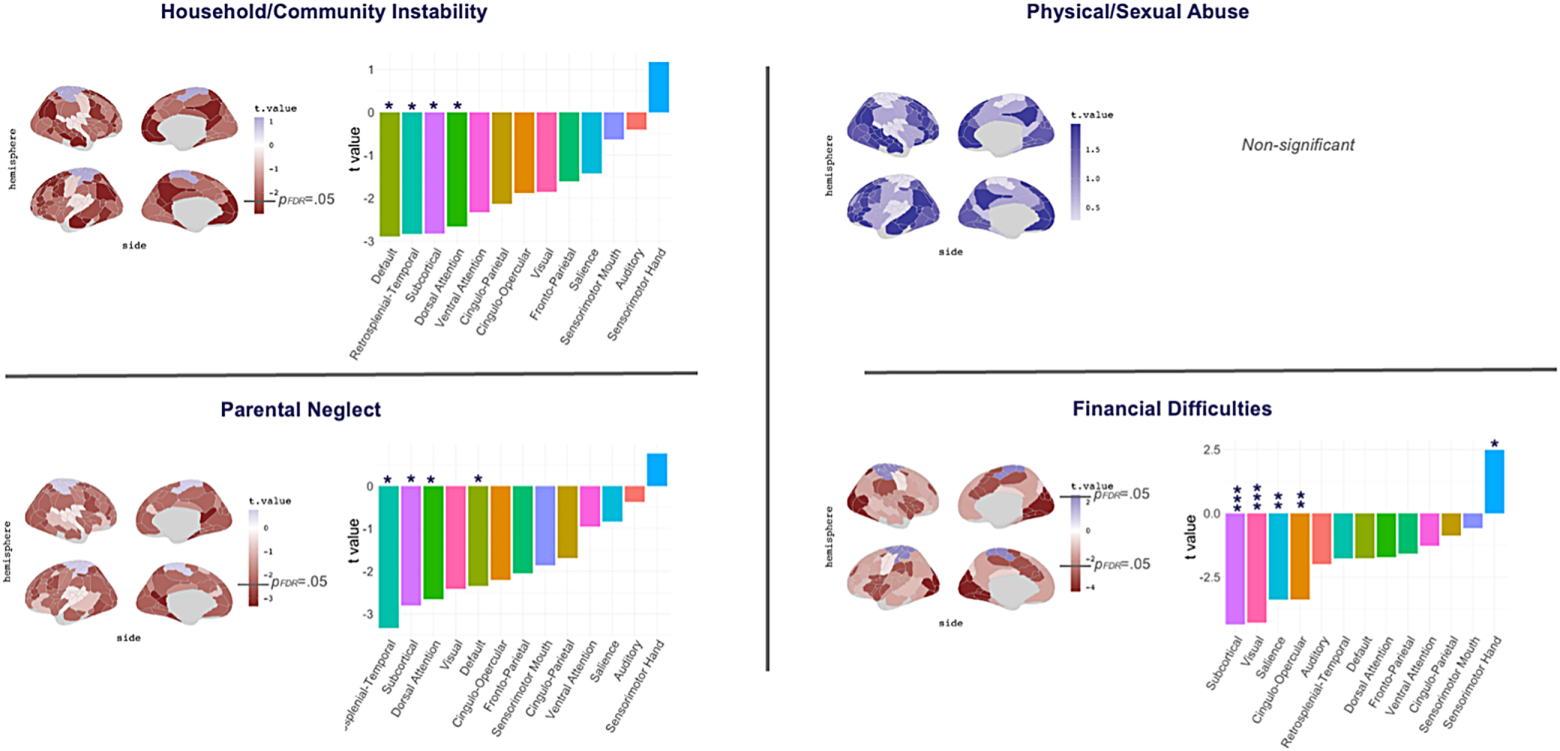
GLM associations between categories of adversity and network-level clustering.*Notes*. Functional networks are defined using the Gordon atlas. Age, sex, scanner type, and scanner head motion were included as covariates. P-values are corrected for the number of Gordon networks (n = 13) using false-discovery rate (FDR;*q*< 0.05). *p< 0.05, **p< 0.01, ***p< 0.001.

**Fig. 5.**
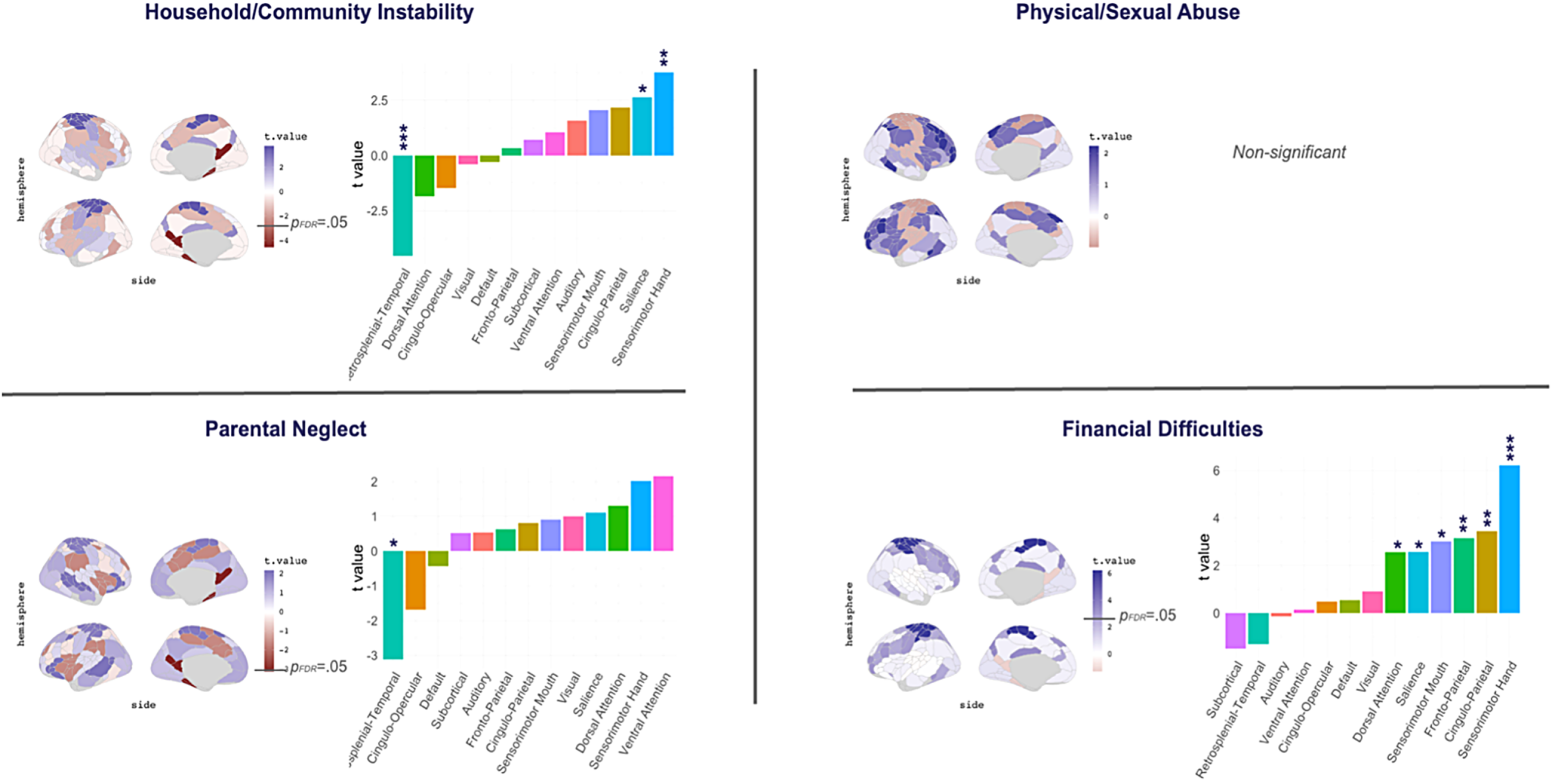
GLM associations between categories of adversity and network-level assortativity.*Notes*. Functional networks are defined using the Gordon atlas. Age, sex, scanner type, and scanner head motion were included as covariates.*p*-Values were corrected for the number of Gordon networks (n = 13) using false-discovery rate (FDR;*q*< 0.05). *p< 0.05, **p< 0.01, ***p< 0.001.

To test whether the effects of adversity varied across functional networks, we repeated our GLMs using the local clustering coefficient and assortativity measure for each 13 Gordon networks. Adversity across the three categories of household/community instability, parental neglect, and financial difficulties had overlapping and unique associations with clustering reductions across various networks, including the cingulo-opercular (COP), dorsal attention (DAN), default, retrosplenial-temporal (RSPT), salience, visual, and subcortical, and an increase in clustering in the sensorimotor hand (SMH;Fig 4;Table S27). These three categories also showed shared and unique associations with assortativity increases in the cingulo-parietal (CPN), DAN, fronto-parietal (FPN), salience, SMH, and SMM, and a reduction in assortativity in the RSPT network (Fig 5;Table S27). There were no significant associations of physical/sexual abuse with network-level clustering or assortativity. Supplementary analyses revealed that all adversity-related differences in network-level topology disappeared after controlling for race/ethnicity and parental education, except for decreased assortativity in the RSPT (Supplement 2.3.1).

### 3.4 Partial least squares

#### 3.4.1 Adversity and global connectome topology

**Fig. 6.**
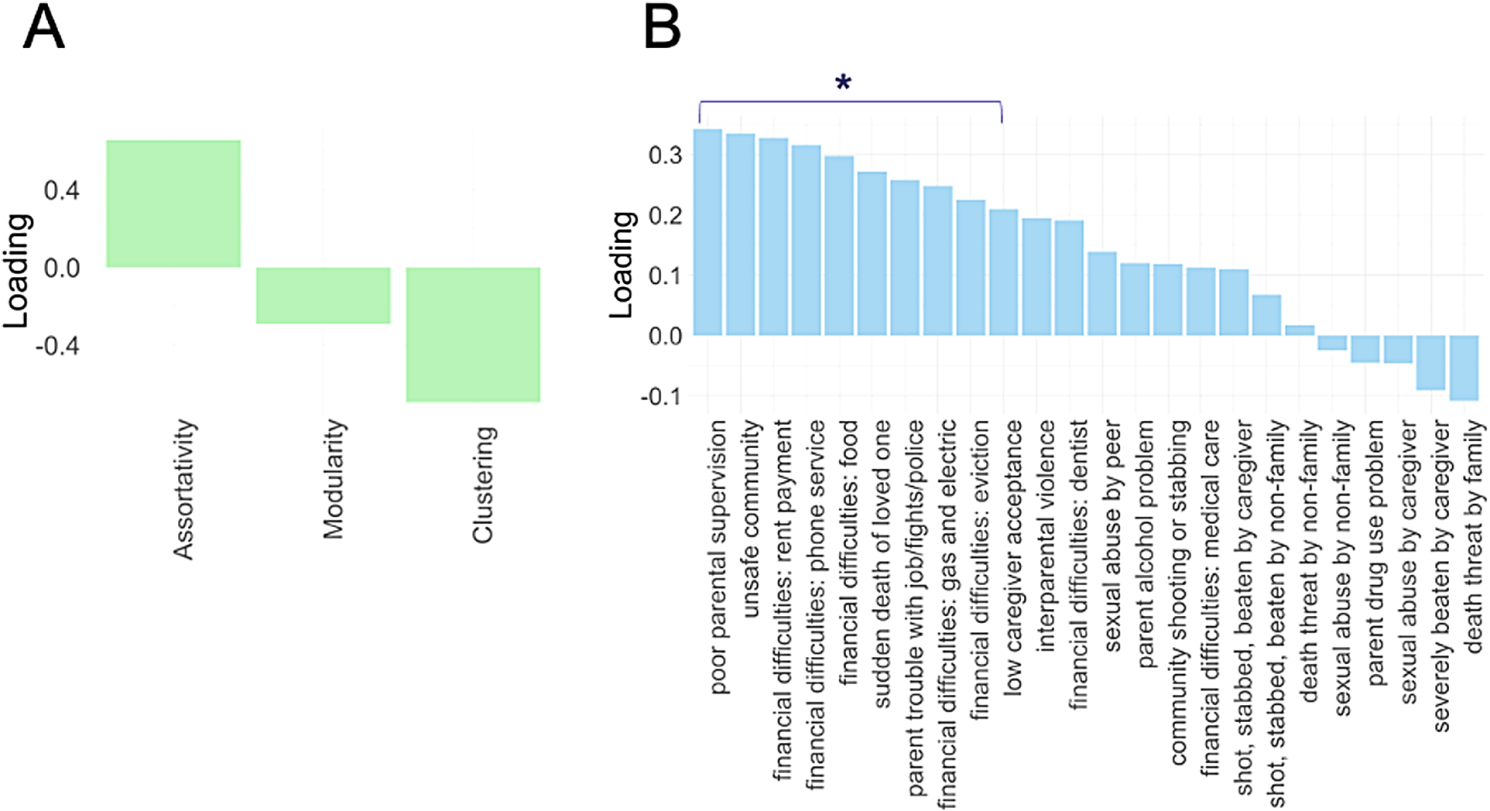
PLS: adversity and global measures.*Notes*. A = PLS global network loadings. B = Adversity items loadings. Star indicates items identified as important based on VIP scores and cross-validation with a significance value of*p*> .05.

Using PLS, we also tested the relationship between individual adversity items and functional connectome topology. We conducted two separate PLS analyses, which correlated the 24 adversity items with (1) global connectome measures and (2) regional (network-level) measures. For the global PLS, the first component explained 13% of the variance in adversity and 59% of the variance in global connectome measures. The correlation between the first pair of latent variables from each set was*r*= 0.1,*p*< .001,*p*perm < .001. Assortativity loaded positively onto the PLS (assortativity loading = 0.66), whereas clustering (clustering loading = -0.70) and modularity (modularity loading = -0.29) loaded negatively, with modularity showing the weakest co-variance with adversity (Fig. 6-A). In other words, these results mirror our GLM findings, of decreased assortativity and increased clustering with greater, and weaker modularity effects. Adversity items identified as important based on VIP scores and cross-validation were from categories of household/community instability, parental neglect, and financial difficulties (Fig. 6-B,Table S28). The correlation between the predictor and response scores after including age, sex, scanner head motion, and scanner type as covariates was*r*= 0.073,*p*< .001. Overall, the PLS broadly mirrored results from the global GLM, with clustering and assortativity loading more strongly than modularity, and physical/sexual abuse showing weaker associations with connectome topology compared to other forms of adversity.

#### 3.4.2 Mediating effects of PLS response scores on mental health

We tested for potential mediating effects of the component response scores on the relationship between the component predictor scores and mental health concurrently and three years later. The PLS predictor score (i.e., a latent score derived from adversity items) was directly associated with greater concurrent internalising (*β*= 1.503, SE = 0.09, z = 17.65,*p*< .001) and externalising difficulties (*β*= 1.684, SE = 0.08, z = 20.97,*p*< .001) controlling for age, sex, scanner motion, and scanner type. Further, the PLS response score (i.e., a latent score derived from global connectome measures) mediated the association between predictor scores and internalising (*β*= -0.036, SE = 0.009, z = 13.85,*p *< .001) and externalising (*β*= -0.019, SE = 0.008, z = -2.423,*p*= .015) difficulties. However, there were no mediating effects of PLS response scores on mental health 3 years later after controlling for baseline symptoms (Tables S29–S30).

#### 3.4.3 Adversity and network-level topology

**Fig. 7.**
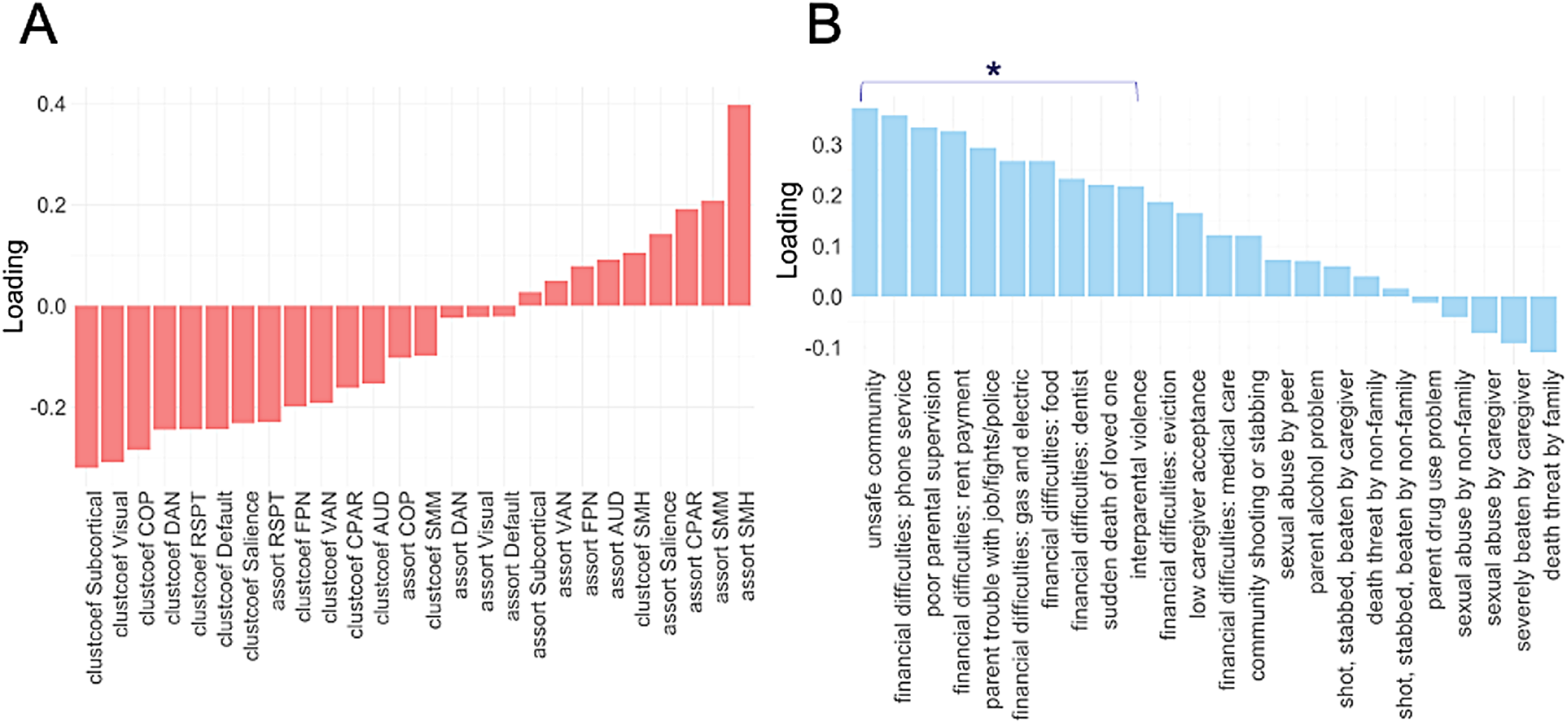
PLS: adversity and network-level measures.*Notes*. A = PLS network-level loadings. B = Adversity items loadings. Star indicates items identified as important based on VIP scores and cross-validation with a significance value of*p*> .05.

For the regional PLS, the first component explained 13% of the variance in adversity and 31% of the variance in network-level measures. The correlation between the first pair of latent variables from each set was*r*= 0.25,*p*< .001,*p*perm < .001. Overall, network-level clustering co-varied more with adversity than assortativity. The strongest clustering effects were reductions in the subcortical, visual, COP, DAN, RSPT, default, and salience networks, whereas the strongest assortativity effects were increases in the SMH and SMM networks, and a reduction in the RSPT respectively (Fig. 7-A;Table S31). Adversity items identified as important based on VIP scores and cross-validation were from categories of household/community instability, parental neglect, and financial difficulties (Fig. 7-B;Table S32). The correlation between the predictor and response scores after including age, sex, scanner head motion, and scanner type as covariates was*r*= 0.19,*p*< .001. Overall, the PLS broadly mirrored results from the network-level clustering GLM. However, assortativity showed weaker overall loadings, suggesting that overlapping variance was being captured by the clustering measures. Supplementary analyses revealed that adding race/ethnicity and parental education as additional covariates decreases the significance of PLS loadings and wash out adversity-related effects in both the global and network-level PLS models (Supplement 2.3.2).

## 4 Discussion

The purpose of this study was to examine the relationship between early life adversity and global and network-level functional brain organisation in children aged 10 (*n*= 5,798) using two complementary approaches: generalised linear models (GLM) and partial least squares (PLS). We first fitted a mixed graphical model and obtained four data-driven categories of adversity: (1) household/community instability; (2) physical/sexual abuse; (3) parental neglect; and (4) financial difficulties. GLM analyses revealed that household/community instability, parental neglect, and financial difficulties were associated with decreased clustering and increased assortativity across the entire brain. None of the adversity categories were significantly related to modularity, suggesting local measures of connectome organisation captured greater adversity-related variance than mesoscale measures. Additionally, there was evidence of a mediating effect of global assortativity on the association between financial difficulties and internalising symptoms. Our regional network-level analyses demonstrate that different categories of adversity have both shared and unique associations with network-level organisation, with financial difficulties showing the most widespread and unique effects across different Gordon networks. In contrast, our PLS results highlight that there is a substantial degree of overlap between different forms of adversity in terms of how they map onto the brain, with the most pronounced cross-category adversity effects in the somatosensory and subcortical networks. Notably, adding race as a covariate attenuated most brain-adversity relationships, suggesting that experiences of early life adversity are strongly related to race/ethnicity in the ABCD sample.

### 4.1 Categories of adversity

Existing dimensional models suggest that dimensions of threat and deprivation alter neurodevelopment in distinct ways (McLaughlin & Sheridan, 2016). Our study suggests some additional nuance by showing that threat-related experiences further split into two distinct categories in this sample, the first of which represents exposures relating to a lack of safety and stability in the environment and the second relating to more direct forms of violence and physical abuse. Deprivation-related experiences also split into more financially-driven versus emotionally-driven aspects of deprivation (i.e., financial difficulties and parental neglect). We identified both shared and unique regional brain differences across the proposed dimensions of adversity, suggesting both overlap across as well as heterogeneity within dimensions of threat and deprivation in terms of how they may influence neurodevelopment. Our results, which we review in detail in the following sections, align with the proposal that environmental instability or unpredictability is a dimension that may have a distinct influence on developmental outcomes (Belsky & De Haan, 2011;Davis et al., 2017;Ellis et al., 2009), but which has been relatively understudied area due to challenges with measuring environmental unpredictability (Young et al., 2020). We found that deprivation-related experiences also split into two distinct categories, the first of which reflected reductions in parental interaction, involvement, and supervision, while the second represented more material forms of financial need. Although there is a wealth of evidence on the developmental implications of financial deprivation, which represents a large component of most measures of household socioeconomic status (SES;A. B. Miller et al., 2018), more emotional aspects of deprivation, including parental neglect, remain relatively understudied beyond the most extreme cases (e.g.,M. Sheridan et al., 2010) due to the lack of adequate measurement tools (Berman et al., 2022). By identifying these four data-driven categories, we were able to explore which aspects of adversity most strongly relate to functional brain organisation and to identify both shared and unique associations while accounting for the high rates of co-occurrence and variability among exposures.

The sensitivity analyses provided novel insights into how different operationalisations of adversity may influence the observed associations with brain and behavioural outcomes. While the categorical approach allowed us to consider distinct adversity types in isolation, the cumulative approach seemed to capture the overlapping and additive effects of multiple adversities, thereby increasing the statistical power by leveraging shared variance. The stronger effects observed with the cumulative score suggest that when adversities are considered collectively, their impact on both brain organisation and behaviour may be amplified. Indeed, our subsequent PLS analyses confirm that adversity, at least in terms of brain organisation, is best represented by a single factor. This is unsurprising given the high rates of co-occurrence and overlap across different adversities (Walsh et al., 2019). However, this finding does not necessarily negate the utility of more complex measures of life experience in research and policy. Indeed, our findings show that financial difficulties emerge as a prepotent predictor of brain and behaviour, with unique network-level effects when examining regional brain variation. Categorical frameworks that are formulated using empirical data and that map onto biological systems in a meaningful way can therefore provide insight into specific types of experiences that may warrant targeted intervention above others.

### 4.2 Global connectome findings

Results from both the PLS and GLM results revealed that experiences of early life adversity were associated with reduced clustering (a measure of local segregation) and increased assortativity across the entire brain. These global patterns were evident across experiences in the form of financial difficulties, household/community instability, and parental neglect, but not physical/sexual abuse. While our findings contrast with previous evidence that children from lower SES households exhibit greater levels of clustering than children from higher SES households (Tooley et al., 2020), a recent review theorised that low-SES children may show earlier developmental increases in functional segregation, whereas high-SES children follow a later but steeper maturational curve, eventually surpassing their lower-SES peers and showing attenuated declines across adulthood (Tooley et al., 2021; see alsoChan et al., 2018). Although our findings are not necessarily at odds with this theory of a faster, yet less refined, trajectory of functional brain maturation in low-SES children, we show that children with fewer financial difficulties already exhibit a more segregated network architecture by age 10, suggesting that this maturational shift may occur earlier than previously proposed (Tooley et al., 2020). However, it is important to note that the measure used here is derived from self-reported financial difficulties and, therefore, captures only one aspect of the broader construct of SES, which traditionally includes income, parental education and other neighbourhood influences (Ursache & Noble, 2016). We found no significant differences in modularity (a mesoscale measure of segregation), consistent with previous findings (Tooley et al., 2020) that SES-related variation in functional network segregation operates primarily at the nodal, rather than mesoscale, level of organisation during childhood.

Increased segregation across development is believed to reflect a greater differentiation of neural activity that occurs from infancy through adolescence (Gao, Alcauter, Elton, et al., 2015;Grayson & Fair, 2017). Growing evidence suggests that this process of connectome segregation supports improvements in information processing and the maturation of higher-order cognitive abilities that underlie behavioural adaptation and problem-solving (Baum et al., 2017;Gu et al., 2015;Wig, 2017). Conversely, reduced levels of segregation following adversity may indicate weaker functional specialisation across brain networks (Fornito et al., 2015;Li et al., 2001). Weaker connectome segregation, or more generalised dysconnectivity between brain networks, has been previously reported across a range of behavioural and mental health conditions (Bassett et al., 2018;Javaheripour et al., 2021;Xia et al., 2018). In contrast, we found no evidence of a mediating effect of reduced clustering on mental health. It is worth noting that since the majority of extant studies have drawn on adult samples with clinical-level difficulties, one possibility is that differences in functional segregation become an increasingly salient marker of mental ill health only in later adolescence and adulthood (Tooley et al., 2021).

Our finding that greater adversity is associated with greater assortativity is particularly notable as few studies have examined this brain parameter in relation to environmental exposures, despite its potential to provide information about network efficiency and robustness (Lim et al., 2019;Noldus & Mieghem, 2015), with possible implications for our understanding of the neural basis of psychopathology (Fornito et al., 2015). Functional connectivity is typically characterised by a disassortative mixing, reflecting its capacity for integration and communication across different regions (Lim et al., 2019). Increased assortativity in technological and biological systems may represent less stable information flow, as well as an enhanced risk for degeneracy in the face of targeted attacks (e.g., removal of links or nodes) and by facilitating the spread of pathological processes (e.g., viruses) throughout the network (Newman, 2002;Vázquez & Moreno, 2003;Zhou et al., 2012). Although it remains a relatively understudied property in network neuroscience, increased functional assortativity has been observed across a range of neurological and mental health conditions (Bassett et al., 2008;Y. Luo et al., 2021;Vo et al., 2021), with the most extreme degree-degree correlations occurring during epileptic seizures (Bialonski & Lehnertz, 2013). The finding that assortativity mediated the association between financial difficulties and internalising symptoms highlights its potential significance for explaining individual differences in mental health outcomes following childhood adversity. While additional longitudinal studies are needed to better elucidate its role in the onset and maintenance of mental health symptoms across development, we did find that network assortativity is significantly related to internalising symptoms. Assortativity is a measure of preferential connection, with nodes connecting to nodes with similar properties. One possibility is that this preferential connectivity is itself an early precursor to subsequent global changes in network integration and segregation. Indeed, in generative networks, the ‘preference for sameness’ is a key ingredient for simulating the formation of plausible biological networks (Akarca et al., 2021) and individual differences in the strength of this preference shape global network properties (Carozza et al., 2023). Put simply, one possibility is that the association between assortativity and mental health cascades over development to encompass a wider set of subsequent network measures.

Overall, our findings suggest that financial difficulties most strongly relate to functional brain organisation compared to other forms of adversity. There are three possible explanations for this finding: (1) Financial difficulties exert a particularly strong influence on development, operating through a variety of mechanistic pathways, including exposure to toxins and stressors, diet, education, the social environment (Jensen et al., 2017;Raphael, 2011); (2) Financial difficulties may have shared or highly correlated genetic susceptibility with measures of brain organisation (Chiang et al., 2009); and (3) Financial difficulties may more readily capture the shared variation across multiple types of commonly co-occurring adversities due to their relative prevalence and importance in shaping the immediate environment (Walsh et al., 2019). Indeed, the last hypothesis aligns with our PLS findings showing that individual items relating to household/community instability and parental supervision were also strongly predictive of whole-brain clustering and assortativity, suggesting that the covariance between these three categories may best explain variation in global patterns of functional brain organisation. These three interrelated explanations are not mutually exclusive and cannot be easily disentangled in the context of complex systems, like the human brain, that emerge probabilistically through dynamic interactions between genetic and environmental influences (Gottlieb, 2007). It is also worth mentioning that financial difficulties were inversely associated with overall edge strength in our sample, suggesting that topological differences were likely driven, in part, by differences in connectivity strength.

Relatedly, our results show that including race as a covariate removes most adversity-related effects, even when statistical models are designed to optimise for covariance between adversity and brain network measures (seesupplementary PLS). Black and Hispanic children in the US have a higher prevalence of poverty and other adverse experiences than their white counterparts (Slopen et al., 2016;U.S. Census Bureau, 2021). Such patterns reflect broader racial and socio-economic disparities across the US (Assari, 2018). Our analyses confirm that race/ethnicity is strongly related to adversity, particularly financial difficulties in the ABCD sample. We, therefore, recognise that it is impossible within this sample to disentangle the experience of early-life adversity and race or ethnicity (see alsoDumornay et al., 2023), because controlling for this source of difference largely eliminates brain-adversity relationships. In our view, this is itself an incredibly important finding and consideration for future research using the ABCD cohort.

### 4.3 Network-level connectome findings

While our global findings suggest that various forms of adversity are associated with comparable patterns of clustering and assortativity across the entire brain, our network-level GLM analyses also reveal unique adversity-related differences across distinct brain networks. Household/community instability and parental neglect were both associated with reduced clustering in the default, retrosplenial temporal (RSPT), and dorsal-attention (DAN) networks and increased assortativity in the RSPT network, indicating significant overlap between these two forms of adverse exposures in terms of functional brain organisation. Conversely, financial difficulties were associated with more widespread and unique alterations across different networks, including in the fronto-parietal (FPN) and cingulo-parietal (CPN), visual, salience, and cingulo-opercular (COP), sensorimotor mouth (SMM), and sensorimotor hand (SMH) networks. These results complement our global GLM finding of a stronger association between financial difficulties and functional brain organisation compared to other forms of adversity. Interestingly, the clustering effect observed in the SMH was inversed from that seen across other networks: greater financial difficulties were associated with*higher*levels of clustering in SMH, a potential marker for heightened sensitivity to sensory input frequently reported in children growing up in poverty (Cassady et al., 2019).

By simultaneously modelling the covariance between individual adversity items and network-level measures, our PLS results additionally highlight that there is a substantial degree of overlap between different forms of adversity in terms of how they map onto the brain. The most pronounced network-level alterations identified by the PLS were clustering reductions in the subcortical network, aligning with our GLM findings of this network being implicated across multiple forms of adversity. The subcortical network is made up of brain regions with a high density of glucocorticoid receptors, making it particularly sensitive to experiences of stress in early life (Teicher et al., 2016). Indeed, functional and morphological alterations in the subcortical network have been previously reported in individuals who experienced early life adversity (Frodl et al., 2017;Loman & Gunnar, 2010). Conversely, the strongest network-level assortativity effects identified by the PLS were in the SMH network, aligning with previous findings of SES-related differences in sensorimotor regions observable from early infancy through adolescence (Gao et al., 2015;Tooley et al., 2020). Alterations in these regions may reflect a tendency for altered sensory processing in those who have experienced adversity (Cassady et al., 2019;Teicher & Samson, 2016) and may play a role in linking early childhood adversities to later mental health difficulties (Bernard et al., 2017;Doucet et al., 2017;Kebets et al., 2019), although further research is needed to bridge these two lines of evidence.

It is also possible that our network-level findings may be capturing developmental timing differences in the regional effects of adversity at age 10 (e.g.,Gao et al., 2015;Gu et al., 2015). Sensorimotor and subcortical networks mature earlier, reaching adult-like properties by late childhood, while higher-order association areas continue to mature throughout adolescence (Gu et al., 2015;Tooley et al., 2021). This developmental sequence may make it easier to observe adversity-related alterations during childhood and adolescence in regions that undergo earlier maturation (Gao et al., 2015;D. J. Miller et al., 2012).

### 4.4 Limitations and future directions

This study has several limitations worth noting. First, it did not consider the timing at which adverse exposures occurred due to limited retrospective data. The brain undergoes substantial change over the first decade of life (Fair et al., 2009;Khundrakpam et al., 2013), with specific sensitive periods to different forms of environmental input (Humphreys & Salo, 2020;Knudsen, 2004). Longitudinal studies with data on the timing, severity, and duration of adverse exposure are necessary to better characterise the impact of adverse experiences in childhood on later outcomes (Cohodes et al., 2021;J. G. Miller et al., 2022). Relatedly, the single imaging timepoint used in this study limits our ability to make developmental inferences, particularly in relation to prior cross-sectional work. Longitudinal imaging studies may offer a more robust framework for observing developmental change and disentangling the temporal aspects of brain organisation following adversity to uncover mechanistic relationships and sensitive periods of susceptibility to environmental influences. Further, our study, which included 363 sibling pairs, did not control for potential family-level confounding due to shared environmental and genetic influences. We would also like to acknowledge that the lack of significant findings in relation to physical/sexual abuse may be due to the (thankfully) relatively low endorsement of this type of adversity in our sample, making it difficult to detect significant effects. This highlights a well-known limitation in prospective adversity research, stemming from reporting biases and attrition rates (Baldwin et al., 2019;Fisher et al., 2011). The exclusion of nearly half of our sample due to excessive head motion, although standard practice in the field (Hagler et al., 2019), raises the potential for selection bias in our findings. The excluded participants had significantly different SES-backgrounds backgrounds, were more likely to be African American, and had higher reported levels adversity. The results of our analyses may therefore reflect brain correlates of early adversity in only a subset of the population that does not include individuals with the highest levels of levels of adversity or from all demographic sectors. This should be considered when interpreting the generalisability of our findings. The challenge of retaining high-adversity participants in neuroimaging studies is well known, but our findings serve to underscore the potential importance of targeted recruitment and retention strategies.

## 5 Conclusion

In summary, our findings demonstrate that adversity is associated with decreased clustering and increased assortativity across the entire brain and that local measures of functional connectome organisation capture greater adversity-related variance than mesoscale measures. Financial difficulties showed the strongest and most widespread associations with functional brain organisation compared to other forms of adversity. The most pronounced effects across multiple forms of adversity were in the somatosensory and subcortical networks. Notably, adding race as a covariate attenuated most brain-adversity relationships, suggesting that experiences of early life adversity are strongly related to race/ethnicity in the ABCD sample—a pattern reflective of widespread racial and socio-economic disparities. Overall, our findings suggest that different forms of adversity are associated with both shared and unique variations in functional brain organisation and highlight the potential significance of assortativity for explaining individual differences in mental health outcomes following childhood adversity.

## Supplementary Material

Supplementary Material

## Data Availability

Data used in this study are held in the ABCD data repository which grows and changes over time. The ABCD data used in this report came from DOI 10.15154/1523041 found athttp://dx.doi.org/10.15154/1523041. Codes that support the findings of this study are available from the corresponding author upon request.

## References

[b1] Achenbach , T. M. ( 2011 ). Child Behavior Checklist . In Encyclopedia of Clinical Neuropsychology (pp. 546 – 552 ). Springer New York . 10.1007/978-0-387-79948-3_1529

[b2] Achenbach , T. M. , & Rescorla , L. A. ( 2001 ). Manual for the ASEBA school age forms & profiles . In ASEBA (pp. 99 – 135 ). https://store.aseba.org/MANUAL-FOR-THE-ASEBA-SCHOOL-AGE-FORMS-PROFILES/productinfo/505/

[b3] Akarca , D. , Vértes , P. E. , Bullmore , E. T. , Baker , K. , Gathercole , S. E. , Holmes , J. , Kievit , R. A. , Manly , T. , Bathelt , J. , Bennett , M. , Bignardi , G. , Bishop , S. , Bottacin , E. , Bridge , L. , Brkic , D. , Bryant , A. , Butterfield , S. , Byrne , E. M. , Crickmore , G. , … Astle , D. E. ( 2021 ). A generative network model of neurodevelopmental diversity in structural brain organization . Nature Communications , 12 ( 1 ), 1 – 18 . 10.1038/s41467-021-24430-z PMC8270998 34244490

[b4] Andersen , S. L. , Tomada , A. , Vincow , E. S. , Valente , E. , Polcari , A. , & Teicher , M. H. ( 2008 ). Preliminary evidence for sensitive periods in the effect of childhood sexual abuse on regional brain development . The Journal of Neuropsychiatry and Clinical Neurosciences , 20 ( 3 ), 292 – 301 . 10.1176/JNP.2008.20.3.292 18806232 PMC4270804

[b5] Assari , S. ( 2018 ). Health disparities due to diminished return among Black Americans: Public policy solutions . Social Issues and Policy Review , 12 ( 1 ), 112 – 145 . 10.1111/SIPR.12042

[b6] Baldwin , J. R. , Reuben , A. , Newbury , J. B. , & Danese , A. ( 2019 ). Agreement between prospective and retrospective measures of childhood maltreatment: A systematic review and meta-analysis . JAMA Psychiatry , 76 ( 6 ), 584 – 593 . 10.1001/JAMAPSYCHIATRY.2019.0097 30892562 PMC6551848

[b7] Barch , D. M. , Albaugh , M. D. , Baskin-Sommers , A. , Bryant , B. E. , Clark , D. B. , Dick , A. S. , Feczko , E. , Foxe , J. J. , Gee , D. G. , Giedd , J. , Glantz , M. D. , Hudziak , J. J. , Karcher , N. R. , LeBlanc , K. , Maddox , M. , McGlade , E. C. , Mulford , C. , Nagel , B. J. , Neigh , G. , … Xie , L. ( 2021 ). Demographic and mental health assessments in the adolescent brain and cognitive development study: Updates and age-related trajectories . Developmental Cognitive Neuroscience , 52 , 1878 – 9293 . 10.1016/J.DCN.2021.101031 PMC8579129 34742018

[b8] Bassett , D. S. , & Bullmore , E. T. ( 2017 ). Small-world brain networks revisited . The Neuroscientist : A Review Journal Bringing Neurobiology, Neurology and Psychiatry , 23 ( 5 ), 499 – 516 . 10.1177/1073858416667720 27655008 PMC5603984

[b9] Bassett , D. S. , Bullmore , E. , Verchinski , B. A. , Mattay , V. S. , Weinberger , D. R. , & Meyer-Lindenberg , A. ( 2008 ). Hierarchical organization of human cortical networks in health and schizophrenia . Journal of Neuroscience , 28 ( 37 ), 9239 – 9248 . 10.1523/JNEUROSCI.1929-08.2008 18784304 PMC2878961

[b10] Bassett , D. S. , & Sporns , O. ( 2017 ). Network neuroscience . Nature Neuroscience , 20 ( 3 ), 353 – 364 . 10.1038/nn.4502 28230844 PMC5485642

[b136] Bassett , D. S. , Xia , C. H. , & Satterthwaite , T. D. ( 2018 ). Understanding the emergence of neuropsychiatric disorders with network neuroscience. Biological psychiatry . Cognitive neuroscience and neuroimaging , 3 ( 9 ), 742 – 753 . 10.1016/j.bpsc.2018.03.015 PMC6119485 29729890

[b11] Baum , G. L. , Ciric , R. , Roalf , D. R. , Betzel , R. F. , Moore , T. M. , Shinohara , R. T. , Kahn , A. E. , Vandekar , S. N. , Rupert , P. E. , Quarmley , M. , Cook , P. A. , Elliott , M. A. , Ruparel , K. , Gur , R. E. , Gur , R. C. , Bassett , D. S. , & Satterthwaite , T. D. ( 2017 ). Modular segregation of structural brain networks supports the development of executive function in youth . Current Biology : CB , 27 ( 11 ), 1561.e8 – 1572.e8 . 10.1016/J.CUB.2017.04.051 28552358 PMC5491213

[b12] Belsky , J. , & De Haan , M. ( 2011 ). Annual research review: Parenting and children’s brain development: The end of the beginning . Journal of Child Psychology and Psychiatry , 52 ( 4 ), 409 – 428 . 10.1111/J.1469-7610.2010.02281.X 20626527

[b13] Benjamini , Y. , & Hochberg , Y. ( 1995 ). Controlling the false discovery rate: A practical and powerful approach to multiple testing . Journal of the Royal Statistical Society: Series B (Methodological) , 57 ( 1 ), 289 – 300 . 10.1111/J.2517-6161.1995.TB02031.X

[b14] Berman , I. S. , McLaughlin , K. A. , Tottenham , N. , Godfrey , K. , Seeman , T. , Loucks , E. , Suomi , S. , Danese , A. , & Sheridan , M. A. ( 2022 ). Measuring early life adversity: A dimensional approach . Development and Psychopathology , 34 ( 2 ), 499 – 511 . 10.1017/S0954579421001826 35314009 PMC7613038

[b15] Bernard , J. A. , Goen , J. R. M. , & Maldonado , T. ( 2017 ). A case for motor network contributions to schizophrenia symptoms: Evidence from resting-state connectivity . Human Brain Mapping , 38 ( 9 ), 4535 – 4545 . 10.1002/HBM.23680 28603856 PMC5547006

[b16] Betzel , R. F. , Bertolero , M. A. , Gordon , E. M. , Gratton , C. , Dosenbach , N. U. F. , & Bassett , D. S. ( 2019 ). The community structure of functional brain networks exhibits scale-specific patterns of inter- and intra-subject variability . NeuroImage , 202 , 115990 . 10.1016/J.NEUROIMAGE.2019.07.003 31291606 PMC7734597

[b17] Betzel , R. F. , Byrge , L. , He , Y. , Goñi , J. , Zuo , X. N. , & Sporns , O. ( 2014 ). Changes in structural and functional connectivity among resting-state networks across the human lifespan . NeuroImage , 102 ( P2 ), 345 – 357 . 10.1016/J.NEUROIMAGE.2014.07.067 25109530

[b18] Betzel , R. F. , Medaglia , J. D. , & Bassett , D. S. ( 2018 ). Diversity of meso-scale architecture in human and non-human connectomes . Nature Communications , 9 ( 1 ), 1 – 14 . 10.1038/s41467-017-02681-z PMC5783945 29367627

[b19] Bialonski , S. , & Lehnertz , K. ( 2013 ). Assortative mixing in functional brain networks during epileptic seizures . Chaos: An Interdisciplinary Journal of Nonlinear Science , 23 ( 3 ), 033139 . 10.1063/1.4821915 24089975

[b20] Biesanz , J. C. , Falk , C. F. , & Savalei , V. ( 2010 ). Assessing mediational models: Testing and interval estimation for indirect effects . Multivariate Behavioral Research , 45 ( 4 ), 661 – 701 . 10.1080/00273171.2010.498292 26735714

[b21] Bignardi , G. , Dalmaijer , E. S. , & Astle , D. E. ( 2022 ). Testing the specificity of environmental risk factors for developmental outcomes . Child Development , 93 ( 3 ), e282 – e298 . 10.1111/CDEV.13719 34936096

[b22] Blondel , V. D. , Guillaume , J. L. , Lambiotte , R. , & Lefebvre , E. ( 2008 ). Fast unfolding of communities in large networks . Journal of Statistical Mechanics: Theory and Experiment , 2008 ( 10 ), P10008 . 10.1088/1742-5468/2008/10/P10008

[b23] Brieant , A. E. , Sisk , L. M. , & Gee , D. G. ( 2021 ). Associations among negative life events, changes in cortico-limbic connectivity, and psychopathology in the ABCD Study . Developmental Cognitive Neuroscience , 52 , 101022 . 10.1016/J.DCN.2021.101022 34710799 PMC8556598

[b24] Buckholtz , J. W. , & Meyer-Lindenberg , A. ( 2012 ). Psychopathology and the human connectome: Toward a transdiagnostic model of risk for mental illness . Neuron , 74 ( 6 ), 990 – 1004 . 10.1016/J.NEURON.2012.06.002 22726830

[b25] Callaghan , B. L. , & Tottenham , N. ( 2016 ). The stress acceleration hypothesis: Effects of early-life adversity on emotion circuits and behavior . Current Opinion in Behavioral Sciences , 7 , 76 – 81 . 10.1016/J.COBEHA.2015.11.018 29644262 PMC5890821

[b26] Cao , M. , Huang , H. , & He , Y. ( 2017 ). Developmental connectomics from infancy through early childhood . Trends in Neurosciences , 40 ( 8 ), 494 – 506 . 10.1016/J.TINS.2017.06.003 28684174 PMC5975640

[b27] Carozza , S. , Holmes , J. , Vértes , P. E. , Bullmore , E. , Arefin , T. M. , Pugliese , A. , Zhang , J. , Kaffman , A. , Akarca , D. , & Astle , D. E. ( 2023 ). Early adversity changes the economic conditions of mouse structural brain network organization . Developmental Psychobiology , 65 ( 6 ). 10.1002/DEV.22405 PMC10505050 37607894

[b28] Casey , B. J. , Cannonier , T. , Conley , M. I. , Cohen , A. O. , Barch , D. M. , Heitzeg , M. M. , Soules , M. E. , Teslovich , T. , Dellarco , D. V. , Garavan , H. , Orr , C. A. , Wager , T. D. , Banich , M. T. , Speer , N. K. , Sutherland , M. T. , Riedel , M. C. , Dick , A. S. , Bjork , J. M. , Thomas , K. M. , … Dale , A. M. ( 2018 ). The Adolescent Brain Cognitive Development (ABCD) study: Imaging acquisition across 21 sites . Developmental Cognitive Neuroscience , 32 , 43 – 54 . 10.1016/J.DCN.2018.03.001 29567376 PMC5999559

[b29] Cassady , K. , Gagnon , H. , Lalwani , P. , Simmonite , M. , Foerster , B. , Park , D. , Peltier , S. J. , Petrou , M. , Taylor , S. F. , Weissman , D. H. , Seidler , R. D. , & Polk , T. A. ( 2019 ). Sensorimotor network segregation declines with age and is linked to GABA and to sensorimotor performance . NeuroImage , 186 , 234 – 244 . 10.1016/J.NEUROIMAGE.2018.11.008 30414983 PMC6338503

[b30] Chan , M. Y. , Na , J. , Agres , P. F. , Savalia , N. K. , Park , D. C. , & Wig , G. S. ( 2018 ). Socioeconomic status moderates age-related differences in the brain’s functional network organization and anatomy across the adult lifespan . Proceedings of the National Academy of Sciences of the United States of America , 115 ( 22 ), E5144 – E5153 . 10.1073/PNAS.1714021115 29760066 PMC5984486

[b31] Chen , A. A. , Srinivasan , D. , Pomponio , R. , Fan , Y. , Nasrallah , I. M. , Resnick , S. M. , Beason-Held , L. L. , Davatzikos , C. , Satterthwaite , T. D. , Bassett , D. S. , Shinohara , R. T. , & Shou , H. ( 2022 ). Harmonizing functional connectivity reduces scanner effects in community detection . NeuroImage , 256 , 119198 . 10.1016/J.NEUROIMAGE.2022.119198 35421567 PMC9202339

[b32] Chen , Y. , & Baram , T. Z. ( 2016 ). Toward understanding how early-life stress reprograms cognitive and emotional brain networks . Neuropsychopharmacology , 41 ( 1 ), 197 – 206 . 10.1038/NPP.2015.181 26105143 PMC4677123

[b33] Chiang , M. C. , Barysheva , M. , Shattuck , D. W. , Lee , A. D. , Madsen , S. K. , Avedissian , C. , Klunder , A. D. , Toga , A. W. , McMahon , K. L. , De Zubicaray , G. I. , Wright , M. J. , Srivastava , A. , Balov , N. , & Thompson , P. M. ( 2009 ). Genetics of brain fiber architecture and intellectual performance . Journal of Neuroscience , 29 ( 7 ), 2212 – 2224 . 10.1523/JNEUROSCI.4184-08.2009 19228974 PMC2773128

[b34] Cohodes , E. M. , Kitt , E. R. , Baskin-Sommers , A. , & Gee , D. G. ( 2021 ). Influences of early-life stress on frontolimbic circuitry: Harnessing a dimensional approach to elucidate the effects of heterogeneity in stress exposure . Developmental Psychobiology , 63 ( 2 ), 153 – 172 . 10.1002/DEV.21969 32227350

[b35] Copeland , W. E. , Shanahan , L. , Hinesley , J. , Chan , R. F. , Aberg , K. A. , Fairbank , J. A. , Van Den Oord , E. J. C. G. , & Costello , E. J. ( 2018 ). Association of childhood trauma exposure with adult psychiatric disorders and functional outcomes . JAMA Network Open , 1 ( 7 ), e184493 – e184493 . 10.1001/JAMANETWORKOPEN.2018.4493 30646356 PMC6324370

[b36] Csárdi , G. , & Nepusz , T. ( 2006 ). The igraph software package for complex network research (No. 1695). InterJournal Complex Systems . https://igraph.org/c/doc/igraph-Introduction.html

[b37] Davis , E. P. , Stout , S. A. , Molet , J. , Vegetabile , B. , Glynn , L. M. , Sandman , C. A. , Heins , K. , Stern , H. , & Baram , T. Z. ( 2017 ). Exposure to unpredictable maternal sensory signals influences cognitive development across species . Proceedings of the National Academy of Sciences of the United States of America , 114 ( 39 ), 10390 – 10395 . 10.1073/PNAS.1703444114 28893979 PMC5625898

[b38] De Bellis , M. D. , & Kuchibhatla , M. ( 2006 ). Cerebellar volumes in pediatric maltreatment-related posttraumatic stress disorder . Biological Psychiatry , 60 ( 7 ), 697 – 703 . 10.1016/J.BIOPSYCH.2006.04.035 16934769

[b39] DeJoseph , M. L. , Herzberg , M. P. , Sifre , R. D. , Berry , D. , & Thomas , K. M. ( 2022 ). Measurement matters: An individual differences examination of family socioeconomic factors, latent dimensions of children’s experiences, and resting state functional brain connectivity in the ABCD sample . Developmental Cognitive Neuroscience , 53 , 101043 . 10.1016/J.DCN.2021.101043 34915436 PMC8683693

[b40] Di Martino , A. , Fair , D. A. , Kelly , C. , Satterthwaite , T. D. , Castellanos , F. X. , Thomason , M. E. , Craddock , R. C. , Luna , B. , Leventhal , B. , Zuo , X. N. , & Milham , M. P. ( 2014 ). Unraveling the miswired connectome: A developmental perspective . Neuron , 83 ( 6 ), 1335 – 1353 . 10.1016/J.NEURON.2014.08.050 25233316 PMC4169187

[b41] Doucet , G. E. , Bassett , D. S. , Yao , N. , Glahn , D. C. , & Frangou , S. ( 2017 ). The role of intrinsic brain functional connectivity in vulnerability and resilience to bipolar disorder . The American Journal of Psychiatry , 174 ( 12 ), 1214 – 1222 . 10.1176/APPI.AJP.2017.17010095 28817956 PMC5711589

[b42] Dumornay , N. M. , Lebois , L. A. M. , Ressler , K. J. , & Harnett , N. G. ( 2023 ). Racial disparities in adversity during childhood and the false appearance of race-related differences in brain structure . The American Journal of Psychiatry , 180 ( 2 ), 127 – 138 . 10.1176/APPI.AJP.21090961 36722118 PMC9897449

[b43] Ellis , B. J. , Figueredo , A. J. , Brumbach , B. H. , & Schlomer , G. L. ( 2009 ). Fundamental dimensions of environmental risk : The impact of harsh versus unpredictable environments on the evolution and development of life history strategies . Human Nature (Hawthorne, N.Y.) , 20 ( 2 ), 204 – 268 . 10.1007/S12110-009-9063-7 25526958

[b44] Epskamp , S. , Cramer , A. O. J. , Waldorp , L. J. , Schmittmann , V. D. , & Borsboom , D. ( 2012 ). qgraph: Network visualizations of relationships in psychometric data . Journal of Statistical Software , 48 . 10.18637/JSS.V048.I04

[b135] Evans , G. W. , Li , D. , & Whipple , S. S. ( 2013 ). Cumulative risk and child development . Psychological bulletin , 139 ( 6 ), 1342 – 1396 . 10.1037/a0031808 23566018

[b45] Fair , D. A. , Cohen , A. L. , Power , J. D. , Dosenbach , N. U. F. , Church , J. A. , Miezin , F. M. , Schlaggar , B. L. , & Petersen , S. E. ( 2009 ). Functional brain networks develop from a “local to distributed” organization . PLoS Computational Biology , 5 ( 5 ), e1000381 . 10.1371/JOURNAL.PCBI.1000381 19412534 PMC2671306

[b46] Farahani , F. V. , Karwowski , W. , & Lighthall , N. R. ( 2019 ). Application of graph theory for identifying connectivity patterns in human brain networks: A systematic review . Frontiers in Neuroscience , 13 ( JUN ), 585 . 10.3389/FNINS.2019.00585 31249501 PMC6582769

[b47] Feczko , E. , Earl , E. , Perrone , A. , & Fair , D. ( 2020 ). ABCD-BIDS community collection (ABCC) . OSF . 10.17605/OSF.IO/PSV5M

[b48] Finkelhor , D. , Ormrod , R. , Turner , H. , & Hamby , S. L. ( 2005 ). The victimization of children and youth: A comprehensive, national survey . Child Maltreatment , 10 ( 1 ), 5 – 25 . 10.1177/1077559504271287 15611323

[b49] Fisher , H. L. , Bunn , A. , Jacobs , C. , Moran , P. , & Bifulco , A. ( 2011 ). Concordance between mother and offspring retrospective reports of childhood adversity . Child Abuse & Neglect , 35 ( 2 ), 117 . 10.1016/J.CHIABU.2010.10.003 21354622 PMC3272365

[b50] Fornito , A. , Zalesky , A. , & Breakspear , M. ( 2015 ). The connectomics of brain disorders . Nature Reviews Neuroscience 2015 16:3 , 16 ( 3 ), 159 – 172 . 10.1038/nrn3901 25697159

[b51] Foygel , R. , & Drton , M. ( 2010 ). Extended Bayesian information criteria for Gaussian graphical models . In Advances in neural information processing systems , 23 . https://papers.nips.cc/paper_files/paper/2010/hash/072b030ba126b2f4b2374f342be9ed44-Abstract.html

[b52] Frodl , T. , Janowitz , D. , Schmaal , L. , Tozzi , L. , Dobrowolny , H. , Stein , D. J. , Veltman , D. J. , Wittfeld , K. , van Erp , T. G. M. , Jahanshad , N. , Block , A. , Hegenscheid , K. , Völzke , H. , Lagopoulos , J. , Hatton , S. N. , Hickie , I. B. , Frey , E. M. , Carballedo , A. , Brooks , S. J. , … Grabe , H. J. ( 2017 ). Childhood adversity impacts on brain subcortical structures relevant to depression . Journal of Psychiatric Research , 86 , 58 . 10.1016/J.JPSYCHIRES.2016.11.010 27918926 PMC5564511

[b53] Galinowski , A. , Miranda , R. , Lemaitre , H. , Paillère Martinot , M. L. , Artiges , E. , Vulser , H. , Goodman , R. , Penttilä , J. , Struve , M. , Barbot , A. , Fadai , T. , Poustka , L. , Conrod , P. , Banaschewski , T. , Barker , G. J. , Bokde , A. , Bromberg , U. , Büchel , C. , Flor , H. , … Rogers , J. ( 2015 ). Resilience and corpus callosum microstructure in adolescence . Psychological Medicine , 45 ( 11 ), 2285 – 2294 . 10.1017/S0033291715000239 25817177

[b54] Gao , W. , Alcauter , S. , Elton , A. , Hernandez-Castillo , C. R. , Smith , J. K. , Ramirez , J. , & Lin , W. ( 2015 ). Functional network development during the first year: Relative sequence and socioeconomic correlations . Cerebral Cortex (New York, N.Y. : 1991) , 25 ( 9 ), 2919 – 2928 . 10.1093/CERCOR/BHU088 24812084 PMC4537436

[b55] Gao , W. , Alcauter , S. , Smith , J. K. , Gilmore , J. H. , & Lin , W. ( 2015 ). Development of human brain cortical network architecture during infancy . Brain Structure & Function , 220 ( 2 ), 1173 – 1186 . 10.1007/S00429-014-0710-3 24469153 PMC4850360

[b56] Garavan , H. , Bartsch , H. , Conway , K. , Decastro , A. , Goldstein , R. Z. , Heeringa , S. , Jernigan , T. , Potter , A. , Thompson , W. , & Zahs , D. ( 2018 ). Recruiting the ABCD sample: Design considerations and procedures . Developmental Cognitive Neuroscience , 32 , 16 – 22 . 10.1016/j.dcn.2018.04.004 29703560 PMC6314286

[b57] Gee , D. G. , Humphreys , K. L. , Flannery , J. , Goff , B. , Telzer , E. H. , Shapiro , M. , Hare , T. A. , Bookheimer , S. Y. , & Tottenham , N. ( 2013 ). A developmental shift from positive to negative connectivity in human amygdala-prefrontal circuitry . Journal of Neuroscience , 33 ( 10 ), 4584 – 4593 . 10.1523/JNEUROSCI.3446-12.2013 23467374 PMC3670947

[b58] Gellci , K. , Marusak , H. A. , Peters , C. , Elrahal , F. , Iadipaolo , A. S. , & Rabinak , C. A. ( 2019 ). Community and household-level socioeconomic disadvantage and functional organization of the salience and emotion network in children and adolescents . NeuroImage , 184 , 729 – 740 . 10.1016/J.NEUROIMAGE.2018.09.077 30287301 PMC6230495

[b59] Gordon , E. M. , Laumann , T. O. , Adeyemo , B. , Huckins , J. F. , Kelley , W. M. , & Petersen , S. E. ( 2016 ). Generation and evaluation of a cortical area parcellation from resting-state correlations . Cerebral Cortex , 26 ( 1 ), 288 – 303 . 10.1093/CERCOR/BHU239 25316338 PMC4677978

[b60] Gottlieb , G. ( 2007 ). Probabilistic epigenesis . Developmental science , 10 ( 1 ), 1 – 11 . 10.1111/j.1467-7687.2007.00556.x 17181692

[b61] Gratton , C. , Kraus , B. T. , Greene , D. J. , Gordon , E. M. , Laumann , T. O. , Nelson , S. M. , Dosenbach , N. U. F. , & Petersen , S. E. ( 2020 ). Defining individual-specific functional neuroanatomy for precision psychiatry . Biological Psychiatry , 88 ( 1 ), 28 – 39 . 10.1016/J.BIOPSYCH.2019.10.026 31916942 PMC7203002

[b62] Grayson , D. S. , & Fair , D. A. ( 2017 ). Development of large-scale functional networks from birth to adulthood: A guide to the neuroimaging literature . NeuroImage , 160 , 15 – 31 . 10.1016/J.NEUROIMAGE.2017.01.079 28161313 PMC5538933

[b63] Gu , S. , Satterthwaite , T. D. , Medaglia , J. D. , Yang , M. , Gur , R. E. , Gur , R. C. , & Bassett , D. S. ( 2015 ). Emergence of system roles in normative neurodevelopment . Proceedings of the National Academy of Sciences of the United States of America , 112 ( 44 ), 13681 – 13686 . 10.1073/PNAS.1502829112 26483477 PMC4640772

[b64] Hagler , D. J. , Hatton , S. N. , Cornejo , M. D. , Makowski , C. , Fair , D. A. , Dick , A. S. , Sutherland , M. T. , Casey , B. J. , Barch , D. M. , Harms , M. P. , Watts , R. , Bjork , J. M. , Garavan , H. P. , Hilmer , L. , Pung , C. J. , Sicat , C. S. , Kuperman , J. , Bartsch , H. , Xue , F. , … Dale , A. M. ( 2019 ). Image processing and analysis methods for the adolescent brain cognitive development study . NeuroImage , 202 . 10.1016/J.NEUROIMAGE.2019.116091 PMC6981278 31415884

[b65] Haslbeck , J. M. B. , Borsboom , D. , & Waldorp , L. J. ( 2019 ). Moderated network models . Multivariate Behavioral Research , 56 ( 2 ), 256 – 287 . 10.1080/00273171.2019.1677207 31782672

[b66] Herzberg , M. P. , & Gunnar , M. R. ( 2020 ). Early life stress and brain function: Activity and connectivity associated with processing emotion and reward . NeuroImage , 209 , 116493 . 10.1016/J.NEUROIMAGE.2019.116493 31884055 PMC7056544

[b67] Herzog , J. I. , & Schmahl , C. ( 2018 ). Adverse childhood experiences and the consequences on neurobiological, psychosocial, and somatic conditions across the lifespan . Frontiers in Psychiatry , 9 , 357654 . 10.3389/FPSYT.2018.00420 PMC6131660 30233435

[b68] Hevey , D. ( 2018 ). Network analysis: A brief overview and tutorial . Health Psychology and Behavioral Medicine , 6 ( 1 ), 301 – 328 . 10.1080/21642850.2018.1521283 34040834 PMC8114409

[b69] Holland , D. , Kuperman , J. M. , & Dale , A. M. ( 2010 ). Efficient correction of inhomogeneous static magnetic field-induced distortion in echo planar imaging . NeuroImage , 50 ( 1 ), 175 – 183 . 10.1016/J.NEUROIMAGE.2009.11.044 19944768 PMC2819607

[b70] Humphreys , K. L. , & Salo , V. C. ( 2020 ). Expectable environments in early life . Current Opinion in Behavioral Sciences , 36 , 115 – 119 . 10.1016/J.COBEHA.2020.09.004 33718532 PMC7945685

[b71] Javaheripour , N. , Li , M. , Chand , T. , Krug , A. , Kircher , T. , Dannlowski , U. , Nenadić , I. , Hamilton , J. P. , Sacchet , M. D. , Gotlib , I. H. , Walter , H. , Frodl , T. , Grimm , S. , Harrison , B. J. , Wolf , C. R. , Olbrich , S. , van Wingen , G. , Pezawas , L. , Parker , G. , … Wagner , G. ( 2021 ). Altered resting-state functional connectome in major depressive disorder: A mega-analysis from the PsyMRI consortium . Translational Psychiatry , 11 ( 1 ), 1 – 9 . 10.1038/s41398-021-01619-w 34620830 PMC8497531

[b72] Jensen , S. K. G. , Berens , A. E. , & Nelson , C. A. ( 2017 ). Effects of poverty on interacting biological systems underlying child development . The Lancet Child and Adolescent Health , 1 ( 3 ), 225 – 239 . 10.1016/S2352-4642(17)30024-X 30169171

[b73] Jones , P. ( 2020 ). networktools: Tools for identifying important nodes in networks version 1.5.0 from CRAN (1.5.0). https://rdrr.io/cran/networktools/

[b74] Jovicich , J. , Czanner , S. , Greve , D. , Haley , E. , Van Der Kouwe , A. , Gollub , R. , Kennedy , D. , Schmitt , F. , Brown , G. , MacFall , J. , Fischl , B. , & Dale , A. ( 2006 ). Reliability in multi-site structural MRI studies: Effects of gradient non-linearity correction on phantom and human data . NeuroImage , 30 ( 2 ), 436 – 443 . 10.1016/J.NEUROIMAGE.2005.09.046 16300968

[b75] Kebets , V. , Holmes , A. J. , Orban , C. , Tang , S. , Li , J. , Sun , N. , Kong , R. , Poldrack , R. A. , & Yeo , B. T. T. ( 2019 ). Somatosensory-motor dysconnectivity spans multiple transdiagnostic dimensions of psychopathology . Biological Psychiatry , 86 ( 10 ), 779 – 791 . 10.1016/J.BIOPSYCH.2019.06.013 31515054

[b76] Kessler , R. C. , Davis , C. G. , & Kendler , K. S. ( 1997 ). Childhood adversity and adult psychiatric disorder in the US National Comorbidity Survey . Psychological Medicine , 27 ( 5 ), 1101 – 1119 . 10.1017/S0033291797005588 9300515

[b77] Kessler , R. C. , McLaughlin , K. A. , Green , J. G. , Gruber , M. J. , Sampson , N. A. , Zaslavsky , A. M. , Aguilar-Gaxiola , S. , Alhamzawi , A. O. , Alonso , J. , Angermeyer , M. , Benjet , C. , Bromet , E. , Chatterji , S. , De Girolamo , G. , Demyttenaere , K. , Fayyad , J. , Florescu , S. , Gal , G. , Gureje , O. , … Williams , D. R. ( 2010 ). Childhood adversities and adult psychopathology in the WHO World Mental Health Surveys . The British Journal of Psychiatry : The Journal of Mental Science , 197 ( 5 ), 378 – 385 . 10.1192/BJP.BP.110.080499 21037215 PMC2966503

[b78] Khundrakpam , B. S. , Reid , A. , Brauer , J. , Carbonell , F. , Lewis , J. , Ameis , S. , Karama , S. , Lee , J. , Chen , Z. , Das , S. , & Evans , A. C. ( 2013 ). Developmental changes in organization of structural brain networks . Cerebral Cortex (New York, N.Y. : 1991) , 23 ( 9 ), 2072 – 2085 . 10.1093/CERCOR/BHS187 22784607 PMC3729193

[b79] Kim , D. J. , Davis , E. P. , Sandman , C. A. , Glynn , L. , Sporns , O. , O’Donnell , B. F. , & Hetrick , W. P. ( 2019 ). Childhood poverty and the organization of structural brain connectome . NeuroImage , 184 , 409 – 416 . 10.1016/J.NEUROIMAGE.2018.09.041 30237035

[b80] Knudsen , E. I. ( 2004 ). Sensitive periods in the development of the brain and behavior . Journal of Cognitive Neuroscience , 16 ( 8 ), 1412 – 1425 . 10.1162/0898929042304796 15509387

[b81] Kolb , B. , Mychasiuk , R. , Muhammad , A. , & Gibb , R. ( 2013 ). Chapter 2—Brain plasticity in the developing brain . Progress in Brain Research , 207 , 35 – 64 . 10.1016/B978-0-444-63327-9.00005-9 24309250

[b82] Kucheryavskiy , S. ( 2020 ). mdatools—R package for chemometrics . Chemometrics and Intelligent Laboratory Systems , 198 . 10.1016/J.CHEMOLAB.2020.103937

[b83] Kuhn , M. ( 2022 ). Classification and Regression Training (Version 6.0-93). Comprehensive R Archive Network (CRAN). https://cran.r-project.org/package=caret

[b84] Li , S. C. , Lindenberger , U. , & Sikström , S. ( 2001 ). Aging cognition: From neuromodulation to representation . Trends in Cognitive Sciences , 5 ( 11 ), 479 – 486 . 10.1016/S1364-6613(00)01769-1 11684480

[b85] Lim , S. , Radicchi , F. , van den Heuvel , M. P. , & Sporns , O. ( 2019 ). Discordant attributes of structural and functional brain connectivity in a two-layer multiplex network . Scientific Reports , 9 ( 1 ), 2885 . 10.1038/S41598-019-39243-W 30814615 PMC6393555

[b86] Loman , M. M. , & Gunnar , M. R. ( 2010 ). Early experience and the development of stress reactivity and regulation in children . Neuroscience & Biobehavioral Reviews , 34 ( 6 ), 867 – 876 . 10.1016/J.NEUBIOREV.2009.05.007 19481109 PMC2848877

[b87] Luo , F. , Li , B. , Wan , X.-F. , & Scheuermann , R. H. ( 2009 ). Core and periphery structures in protein interaction networks . BMC Bioinformatics , 10 ( Suppl 4 ), S8 . 10.1186/1471-2105-10-S4-S8 PMC2681073 19426456

[b88] Luo , Y. , Sun , T. , Ma , C. , Zhang , X. , Ji , Y. , Fu , X. , & Ni , H. ( 2021 ). Alterations of brain networks in Alzheimer’s disease and mild cognitive impairment: A resting state fMRI study based on a population-specific brain template . Neuroscience , 452 , 192 – 207 . 10.1016/J.NEUROSCIENCE.2020.10.023 33197505

[b89] McLaughlin , K. A. , Green , J. G. , Gruber , M. J. , Sampson , N. A. , Zaslavsky , A. M. , & Kessler , R. C. ( 2012 ). Childhood adversities and first onset of psychiatric disorders in a national sample of US adolescents . Archives of General Psychiatry , 69 ( 11 ), 1151 – 1160 . 10.1001/ARCHGENPSYCHIATRY.2011.2277 23117636 PMC3490224

[b90] McLaughlin , K. A. , & Sheridan , M. A. ( 2016 ). Beyond cumulative risk: A dimensional approach to childhood adversity . Current Directions in Psychological Science , 25 ( 4 ), 239 – 245 . 10.1177/0963721416655883 27773969 PMC5070918

[b91] McLaughlin , K. A. , Sheridan , M. A. , Humphreys , K. L. , Belsky , J. , & Ellis , B. J. ( 2021 ). The value of dimensional models of early experience: Thinking clearly about concepts and categories . Perspectives on Psychological Science , 16 ( 6 ), 1463 – 1472 . 10.1177/1745691621992346 34491864 PMC8563369

[b92] Miller , A. B. , Sheridan , M. A. , Hanson , J. L. , McLaughlin , K. A. , Bates , J. E. , Lansford , J. E. , Pettit , G. S. , & Dodge , K. A. ( 2018 ). Dimensions of deprivation and threat, psychopathology, and potential mediators: A multi-year longitudinal analysis . Journal of Abnormal Psychology , 127 ( 2 ), 160 – 170 . 10.1037/ABN0000331 29528670 PMC5851283

[b93] Miller , D. J. , Duka , T. , Stimpson , C. D. , Schapiro , S. J. , Baze , W. B. , McArthur , M. J. , Fobbs , A. J. , Sousa , A. M. M. , Sěstan , N. , Wildman , D. E. , Lipovich , L. , Kuzawa , C. W. , Hof , P. R. , & Sherwood , C. C. ( 2012 ). Prolonged myelination in human neocortical evolution . Proceedings of the National Academy of Sciences of the United States of America , 109 ( 41 ), 16480 – 16485 . 10.1073/PNAS.1117943109 23012402 PMC3478650

[b94] Miller , G. A. , & Chapman , J. P. ( 2001 ). Misunderstanding analysis of covariance . Journal of Abnormal Psychology , 110 ( 1 ), 40 – 48 . 10.1037/0021-843X.110.1.40 11261398

[b95] Miller , J. G. , Chahal , R. , & Gotlib , I. H. ( 2022 ). Early life stress and neurodevelopment in adolescence: Implications for risk and adaptation . In K. A. Miczek & R. Sinha (Eds.), Neuroscience of social stress (pp. 313 – 339 ). Springer . 10.1007/7854_2022_302 35290658

[b96] Newman , M. E. J. ( 2002 ). Assortative mixing in networks . Physical Review Letters , 89 ( 20 ), 208701 . 10.1103/PHYSREVLETT.89.208701 12443515

[b97] Newman , M. E. J. ( 2004 ). Analysis of weighted networks . Physical Review E - Statistical Physics, Plasmas, Fluids, and Related Interdisciplinary Topics , 70 ( 5 ), 9 . 10.1103/PHYSREVE.70.056131

[b98] Noldus , R. , & Mieghem , P. V. ( 2015 ). Assortativity in complex networks . Journal of Complex Networks , 3 ( 4 ), 507 – 542 . 10.1093/COMNET/CNV005

[b99] Pons , P. , & Latapy , M. ( 2006 ). Computing communities in large networks using random walks . Journal of Graph Algorithms and Applications , 10 ( 2 ), 191 – 218 . http://jgaa.info/vol http://www.liafa.jussieu.fr/

[b100] R Core Team . ( 2021 ). A language and environment for statistical computing . (4.0.3). R Foundation for Statistical Computing . https://www.r-project.org/

[b101] Raphael , D. ( 2011 ). Poverty in childhood and adverse health outcomes in adulthood . Maturitas , 69 ( 1 ), 22 – 26 . 10.1016/J.MATURITAS.2011.02.011 21398059

[b102] Rohart , F. , Gautier , B. , Singh , A. , & Lê Cao , K. A. ( 2017 ). mixOmics: An R package for ‘omics feature selection and multiple data integration . PLOS Computational Biology , 13 ( 11 ), e1005752 . 10.1371/JOURNAL.PCBI.1005752 29099853 PMC5687754

[b103] Rubinov , M. , & Sporns , O. ( 2010 ). Complex network measures of brain connectivity: Uses and interpretations . NeuroImage , 52 ( 3 ), 1059 – 1069 . 10.1016/J.NEUROIMAGE.2009.10.003 19819337

[b104] Rubinov , M. , & Sporns , O. ( 2011 ). Weight-conserving characterization of complex functional brain networks . NeuroImage , 56 ( 4 ), 2068 – 2079 . 10.1016/J.NEUROIMAGE.2011.03.069 21459148

[b105] Satterthwaite , T. D. , Wolf , D. H. , Loughead , J. , Ruparel , K. , Elliott , M. A. , Hakonarson , H. , Gur , R. C. , & Gur , R. E. ( 2012 ). Impact of in-scanner head motion on multiple measures of functional connectivity: Relevance for studies of neurodevelopment in youth . NeuroImage , 60 ( 1 ), 623 . 10.1016/J.NEUROIMAGE.2011.12.063 22233733 PMC3746318

[b106] Searle , S. R. , Speed , F. M. , & Milliken , G. A. ( 2022 ). Estimated marginal means, aka least-squares means [R package emmeans version 1.8.1-1] . American Statistician , 34 ( 4 ), 216 – 221 . 10.1080/00031305.1980.10483031

[b107] Sheridan , M. A. , Fox , N. A. , Zeanah , C. H. , McLaughlin , K. A. , & Nelson , C. A. ( 2012 ). Variation in neural development as a result of exposure to institutionalization early in childhood . Proceedings of the National Academy of Sciences of the United States of America , 109 ( 32 ), 12927 – 12932 . 10.1073/PNAS.1200041109 22826224 PMC3420193

[b108] Sheridan , M. , Drury , S. , McLaughlin , K. , & Almas , A. ( 2010 ). Early institutionalization: Neurobiological consequences and genetic modifiers . Neuropsychology Review , 20 ( 4 ), 414 . 10.1007/S11065-010-9152-8 21042937 PMC3100174

[b109] Slopen , N. , Kubzansky , L. D. , McLaughlin , K. A. , & Koenen , K. C. ( 2013 ). Childhood adversity and inflammatory processes in youth: A prospective study . Psychoneuroendocrinology , 38 ( 2 ), 188 – 200 . 10.1016/J.PSYNEUEN.2012.05.013 22727478 PMC3632283

[b110] Slopen , N. , Shonkoff , J. P. , Albert , M. A. , Yoshikawa , H. , Jacobs , A. , Stoltz , R. , & Williams , D. R. ( 2016 ). Racial disparities in child adversity in the U.S.: Interactions with family immigration history and income . American Journal of Preventive Medicine , 50 ( 1 ), 47 – 56 . 10.1016/J.AMEPRE.2015.06.013 26342634

[b111] Smith , K. E. , & Pollak , S. D. ( 2020 ). Rethinking concepts and categories for understanding the neurodevelopmental effects of childhood adversity . Perspectives on Psychological Science , 16 ( 1 ), 67 – 93 . 10.1177/1745691620920725 32668190 PMC7809338

[b112] Sporns , O. , & Rubinov , M. ( 2019 ). Brain connectivity toolbox . http://www.brain-connectivity-toolbox.net

[b113] Sripada , R. K. , Swain , J. E. , Evans , G. W. , Welsh , R. C. , & Liberzon , I. ( 2014 ). Childhood poverty and stress reactivity are associated with aberrant functional connectivity in default mode network . Neuropsychopharmacology : Official Publication of the American College of Neuropsychopharmacology , 39 ( 9 ), 2244 – 2251 . 10.1038/NPP.2014.75 24675708 PMC4104343

[b114] Struck , N. , Krug , A. , Yuksel , D. , Stein , F. , Schmitt , S. , Meller , T. , Brosch , K. , Dannlowski , U. , Nenadić , I. , Kircher , T. , & Brakemeier , E. L. ( 2020 ). Childhood maltreatment and adult mental disorders—The prevalence of different types of maltreatment and associations with age of onset and severity of symptoms . Psychiatry Research , 293 , 113398 . 10.1016/J.PSYCHRES.2020.113398 32920524

[b115] Teicher , M. H. , Anderson , C. M. , & Polcari , A. ( 2012 ). Childhood maltreatment is associated with reduced volume in the hippocampal subfields CA3, dentate gyrus, and subiculum . Proceedings of the National Academy of Sciences of the United States of America , 109 ( 9 ), E563 – E572 . 10.1073/PNAS.1115396109 22331913 PMC3295326

[b116] Teicher , M. H. , & Samson , J. A. ( 2016 ). Annual research review: Enduring neurobiological effects of childhood abuse and neglect . Journal of Child Psychology and Psychiatry, and Allied Disciplines , 57 ( 3 ), 241 . 10.1111/JCPP.12507 26831814 PMC4760853

[b117] Teicher , M. H. , Samson , J. A. , Anderson , C. M. , & Ohashi , K. ( 2016 ). The effects of childhood maltreatment on brain structure, function and connectivity . Nature Reviews Neuroscience 2016 17:10 , 17 ( 10 ), 652 – 666 . 10.1038/nrn.2016.111 27640984

[b118] Thedchanamoorthy , G. , Piraveenan , M. , Kasthuriratna , D. , & Senanayake , U. ( 2014 ). Node assortativity in complex networks: An alternative approach . Procedia Computer Science , 29 , 2449 – 2461 . 10.1016/J.PROCS.2014.05.229

[b119] Tibshirani , R. ( 1996 ). Regression Shrinkage and Selection Via the Lasso . Journal of the Royal Statistical Society: Series B (Methodological) , 58 ( 1 ), 267 – 288 . 10.1111/J.2517-6161.1996.TB02080.X

[b120] Tooley , U. A. , Bassett , D. S. , & Mackey , A. P. ( 2021 ). Environmental influences on the pace of brain development . Nature Reviews Neuroscience , 22 ( 6 ), 372 – 384 . 10.1038/s41583-021-00457-5 33911229 PMC8081006

[b121] Tooley , U. A. , MacKey , A. P. , Ciric , R. , Ruparel , K. , Moore , T. M. , Gur , R. C. , Gur , R. E. , Satterthwaite , T. D. , & Bassett , D. S. ( 2020 ). Associations between neighborhood SES and functional brain network development . Cerebral Cortex (New York, N.Y. : 1991) , 30 ( 1 ), 1 – 19 . 10.1093/CERCOR/BHZ066 31220218 PMC7029704

[b122] Ursache , A. , & Noble , K. G. ( 2016 ). Neurocognitive development in socioeconomic context: Multiple mechanisms and implications for measuring socioeconomic status . Psychophysiology , 53 ( 1 ), 71 – 82 . 10.1111/PSYP.12547 26681619 PMC4685721

[b123] US Census Bureau . ( 2021 ). Income and Poverty in the United States: 2019 . Current Population Survey Annual Social and Economic Supplements (CPS ASEC) . https://www.census.gov/library/publications/2020/demo/p60-270.html

[b124] van den Heuvel , M. P. , de Lange , S. C. , Zalesky , A. , Seguin , C. , Yeo , B. T. T. , & Schmidt , R. ( 2017 ). Proportional thresholding in resting-state fMRI functional connectivity networks and consequences for patient-control connectome studies: Issues and recommendations . NeuroImage , 152 , 437 – 449 . 10.1016/J.NEUROIMAGE.2017.02.005 28167349

[b125] Van Dijk , K. R. A. , Hedden , T. , Venkataraman , A. , Evans , K. C. , Lazar , S. W. , & Buckner , R. L. ( 2010 ). Intrinsic functional connectivity as a tool for human connectomics: Theory, properties, and optimization . Journal of Neurophysiology , 103 ( 1 ), 297 – 321 . 10.1152/JN.00783.2009 19889849 PMC2807224

[b126] Vázquez , A. , & Moreno , Y. ( 2003 ). Resilience to damage of graphs with degree correlations . Physical Review E , 67 ( 1 ), 015101 . 10.1103/PhysRevE.67.015101 12636544

[b127] Vo , A. , Schindlbeck , K. , Nguyen , N. , Rommal , A. , Niethammer , M. , & Eidelgerg , D. ( 2021 ). Resting state functional MRI network assortativity is increased in Parkinson’s disease—MDS Abstracts . In MDS Virtual Congress 2021 . https://www.mdsabstracts.org/abstract/resting-state-functional-mri-network-assortativity-is-increased-in-parkinsons-disease/

[b128] Walsh , D. , McCartney , G. , Smith , M. , & Armour , G. ( 2019 ). Relationship between childhood socioeconomic position and adverse childhood experiences (ACEs): A systematic review . Journal of Epidemiology & Community Health , 73 ( 12 ), 1087 – 1093 . 10.1136/JECH-2019-212738 31563897 PMC6872440

[b129] Wig , G. S. ( 2017 ). Segregated systems of human brain networks . Trends in Cognitive Sciences , 21 ( 12 ), 981 – 996 . 10.1016/J.TICS.2017.09.006 29100737

[b130] Wold , S. , Sjöström , M. , & Eriksson , L. ( 2001 ). PLS-regression: A basic tool of chemometrics . Chemometrics and Intelligent Laboratory Systems , 58 ( 2 ), 109 – 130 . 10.1016/S0169-7439(01)00155-1

[b131] Xia , C. H. , Ma , Z. , Ciric , R. , Gu , S. , Betzel , R. F. , Kaczkurkin , A. N. , Calkins , M. E. , Cook , P. A. , García de la Garza , A. , Vandekar , S. N. , Cui , Z. , Moore , T. M. , Roalf , D. R. , Ruparel , K. , Wolf , D. H. , Davatzikos , C. , Gur , R. C. , Gur , R. E. , Shinohara , R. T. , … Satterthwaite , T. D. ( 2018 ). Linked dimensions of psychopathology and connectivity in functional brain networks . Nature Communications 2018 9:1 , 9 ( 1 ), 1 – 14 . 10.1038/s41467-018-05317-y PMC6070480 30068943

[b132] Young , E. S. , Frankenhuis , W. E. , & Ellis , B. J. ( 2020 ). Theory and measurement of environmental unpredictability . Evolution and Human Behavior , 41 ( 6 ), 550 – 556 . 10.1016/J.EVOLHUMBEHAV.2020.08.006

[b133] Zhang , B. , & Horvath , S. ( 2005 ). A general framework for weighted gene co-expression network analysis . Statistical Applications in Genetics and Molecular Biology , 4 ( 1 ). 10.2202/1544-6115.1128 16646834

[b134] Zhou , D. , D’Agostino , G. , Scala , A. , & Stanley , H. E. ( 2012 ). Assortativity decreases the robustness of interdependent networks . Physical Review E - Statistical, Nonlinear, and Soft Matter Physics , 86 ( 6 ). 10.1103/PhysRevE.86.066103 23368000

