# Supplementary material for: Dimensions of early life adversity and their associations with functional brain organisation
Source: Imaging Neurosci (Camb). 2024 Apr 26;2:imag-2-00145. doi: 10.1162/imag_a_00145 (PMC12247618; doi:10.1162/imag_a_00145)
Supplement: Supplementary Material [file imag_a_00145-supp.pdf]

1. METHODS SUPPLEMENT

Table S1. Demographics for all ABCD participants at baseline

N	11,876
Age mean *(SD)*	9.9 *(0.6)*
% Female	47.8%
Race/Ethnicity	
% White	52.2%
% African American	15.1%
% Hispanic	3.9%
% Asian	2.2%
% Other/Multi-racial	25.9%
Household characteristics	
% Married caregivers	65.5%
% College-level education	59.4%
Household income	
< $25k	17.2%
$25k - $49.99k	22.4%
$50k -$74.99k	14.5%
$75k -$99.99k	30.5%
$100k +	15.4%

Notes. Age reported in years. College education reported if one or more caregivers has a college-level degree.

Table S2. Adversity questions

Description	ABCD variable name	Category	Timescale	% missing
parent alcohol problem	famhx_ss_momdad_alc_p	Household/community instability	Lifetime (age 0-9)	3.77
parent drug use problem	famhx_ss_momdad_dg_p	Household/community instability	Lifetime (age 0-9)	5.45
parent trouble with job/fights/police	famhx_ss_momdad_trb_p	Household/community instability	Lifetime (age 0-9)	3.05
community shooting or stabbing	ksads_ptsd_raw_760_p	Household/community instability	Lifetime (age 0-9)	2.41
shot, stabbed, beaten by non-family	ksads_ptsd_raw_761_p	Physical/sexual abuse	Lifetime (age 0-9)	2.41
shot, stabbed, beaten by caregiver	ksads_ptsd_raw_762_p	Physical/sexual abuse	Lifetime (age 0-9)	2.41
severely beaten by caregiver	ksads_ptsd_raw_763_p	Physical/sexual abuse	Lifetime (age 0-9)	2.41
death threat by non-family	ksads_ptsd_raw_764_p	Physical/sexual abuse	Lifetime (age 0-9)	2.41
death threat by family	ksads_ptsd_raw_765_p	Physical/sexual abuse	Lifetime (age 0-9)	2.41
interparental violence	ksads_ptsd_raw_766_p	Household/community instability	Lifetime (age 0-9)	2.41
sexual abuse by caregiver	ksads_ptsd_raw_767_p	Physical/sexual abuse	Lifetime (age 0-9)	2.41
sexual abuse by non-family	ksads_ptsd_raw_768_p	Physical/sexual abuse	Lifetime (age 0-9)	2.41
sexual abuse by peer	ksads_ptsd_raw_769_p	Physical/sexual abuse	Lifetime (age 0-9)	2.41
unsafe community	nsc_p_ss_mean_3_items	Household/community instability	Lifetime (age 0-9)	0.07
poor parental supervision	pmq_y_ss_mean	Parental neglect	Lifetime (age 0-9)	0.18
low caregiver acceptance	crpbi_y_ss_parent	Parental neglect	Lifetime (age 0-9)	0.29
financial difficulties: food	demo_fam_exp1_v2	Financial difficulties	Past 12 months	0.64
financial difficulties: phone service	demo_fam_exp2_v2	Financial difficulties	Past 12 months	0.39
financial difficulties: rent payment	demo_fam_exp3_v2	Financial difficulties	Past 12 months	0.51
financial difficulties: eviction	demo_fam_exp4_v2	Financial difficulties	Past 12 months	0.28
financial difficulties: gas and electric	demo_fam_exp5_v2	Financial difficulties	Past 12 months	0.37

financial difficulties: medical care	demo_fam_exp6_v2	Financial difficulties	Past 12 months	0.34
financial difficulties: dentist	demo_fam_exp7_v2	Financial difficulties	Past 12 months	0.41
sudden death of loved one	ksads_ptsd_raw_770_p	Household/community instability	Lifetime (age 0-9)	2.41
death/mass destruction in war	ksads_ptsd_raw_759_p	NA	Lifetime (age 0-9)	2.41
family conflict	fes_y_ss_fc_pr	NA	Lifetime (age 0-9)	0.20
serious car accident	ksads_ptsd_raw_754_p	NA	Lifetime (age 0-9)	2.41
other serious accident	ksads_ptsd_raw_755_p	NA	Lifetime (age 0-9)	2.41
injury/destruction from fire	ksads_ptsd_raw_756_p	NA	Lifetime (age 0-9)	2.41
injury/destruction from natural disaster	ksads_ptsd_raw_757_p	NA	Lifetime (age 0-9)	2.41
witness act of terrorism	ksads_ptsd_raw_758_p	NA	Lifetime (age 0-9)	2.41

Notes. Adversity items taken at baseline assessment (T1). Parental-report used. Categories obtained using mixed-graphical models (see main manuscript). NA category represents items that did not cluster with any other network nodes. Missingness represents the percentage of participants with missing data on a given question.

1.2 Measuring early life adversity

Missing adversity data was coded as “0” because sensitivity analyses revealed that either coding it as 1 or imputing it resulted in an overestimation of adversity in the sample relative to population prevalence estimates (Finkelhor et al., 2005; McLaughlin et al., 2012; Struck et al., 2020). In the first sensitivity analysis, we coded missing data as 1 instead of 0. This resulted in an additional 524 participants with adversity exposures, and an ELA group representing 27% of the total sample. This was much higher than population prevalence estimates would suggest, meaning this approach was heavily biased unlikely representative of real-world data.

Next, we used a multiple imputation package for mixed-type data, missForest in R (Stekhoven et al., 2012) to impute the missing values. Imputing missing values resulted in an additional 512 participants with adversity exposures (1251 in; 26.5% of the

total sample), again much higher than population prevalence estimates (Finkelhor et al., 2005; McLaughlin et al., 2012; Struck et al., 2020). Imputation algorithms are heavily biased towards rare cases with binary data (e.g., exposures to ELA). This means adversity is likely over-estimated, explaining why using this method resulted in an unusually high number of children classified as having experienced adversity relative to population prevalence estimates (Finkelhor et al., 2005; McLaughlin et al., 2012; Struck et al., 2020). There were other reasons for not using imputation. First, it would have increased the standard error, which would have been problematic for our subsequent analyses. Second, the data were not missing at random. We tested for associations between missingness and several key cognitive and demographic variables. Missingness was associated interview ethnicity ($p<.001$); parental education ($p<.001$); parental income ($p<.001$) and 3 out of 5 measures of cognition that were tested (*p*s= .001-.05). The missing at random assumption was therefore not plausible, meaning that imputation would be heavily biased. Although imputation is beneficial in some cases, it must be balanced against the possible risks of inducing bias and overfitting, particularly in the case of non-normally distributed binary data (Sterne et al., 2009). While some procedures can handle non-normally distributed data better than others (Van Buuren et al., 1999), it is an ongoing area of development (Horton et al., 2007; Bernaards et al, 2007) that currently has no well-defined solution (Lee & Carlin, 2016; Sullivan et al., 2017). For these reasons, we decided imputation was not appropriate for our data.

1.3 Diagnostic tests for linearity and multicollinearity

To ensure the appropriateness of employing Generalized Linear Models (GLMs) for our analysis, we undertook a series of diagnostic tests to evaluate the linearity of the relationships between each predictor and the outcome variable. Q-Q plots were generated for the residuals of a preliminary linear regression model to compare against a normal distribution. Residuals following a straight line indicate that the normality assumption is not violated. Residual plots were generated to plot the residuals against fitted values (with constant-information scale transformations) to visually inspect for non-linear patterns. Patterns or systematic deviations would suggest non-linearity or heteroscedasticity. Partial residual plots used to isolate the relationship between each predictor and the

outcome, adjusting for the presence of other predictors. A non-random pattern would indicate a non-linear relationship or interaction effects that are not captured by the model. The absence of systematic patterns in the below plots led us to conclude that the assumption of linearity was not violated.

Multicollinearity can inflate the variance of the coefficient estimates and make the model unstable. To check for this, we used the Variance Inflation Factor (VIF). A VIF value exceeding 10 was considered indicative of significant multicollinearity, while a value below 4 and above 0.25 is typically regarded as acceptable. No VIF values exceeded these thresholds (reported below). Consequently, we determined that multicollinearity was not a concern that would prevent the use of GLMs.

1.3.1 Clustering

Q-Q plot: Standardized deviance residuals

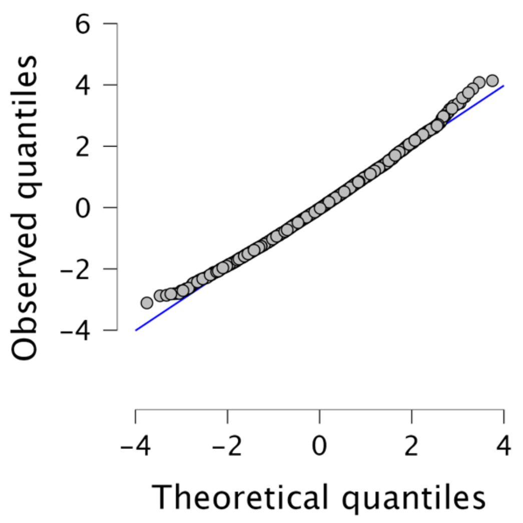

Residuals vs Fitted Plot

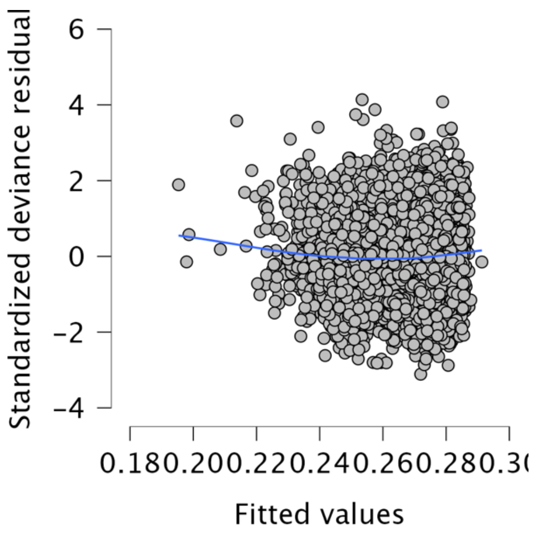

Partial Residual Plots

Partial residual plot for Household & Community Instability

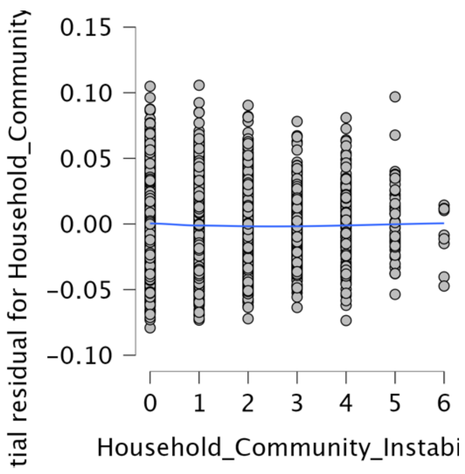

Partial residual plot for Physical & Sexual Abuse

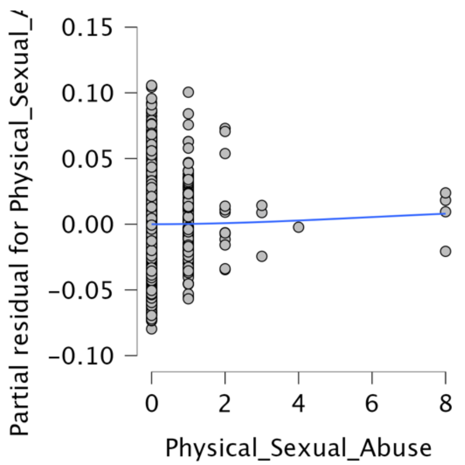

Partial residual plot for Parental Neglect

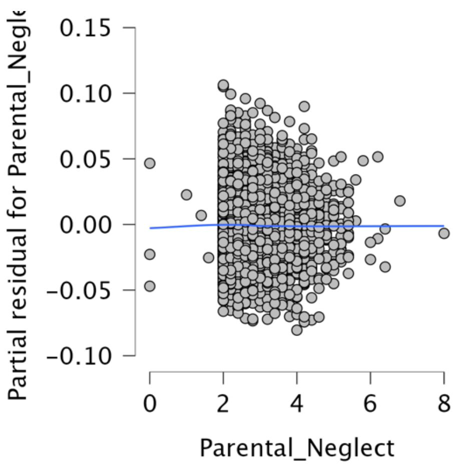

Partial residual plot for Financial Difficulties

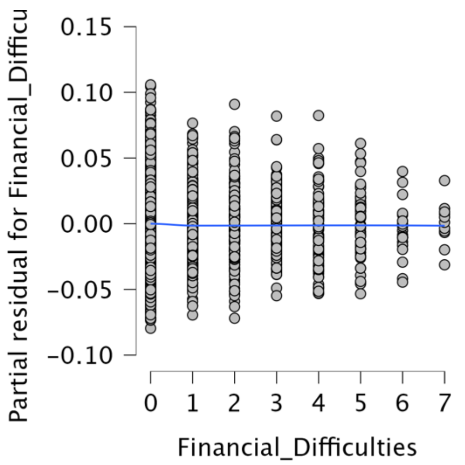

Multicollinearity Diagnostics

	**VIF**
Household Community Instability	1.156
Physical Sexual Abuse	1.034
Parental Neglect	1.042
Financial Difficulties	1.179
Age	1.019
Mean head motion	1.034
Sex	1.029
Scanner type	1.038
Race/ethnicity	1.132

Notes. VIF above 4 or below 0.25 indicates that multicollinearity may exist.

1.3.2 Modularity

Q-Q plot: Standardized deviance residuals

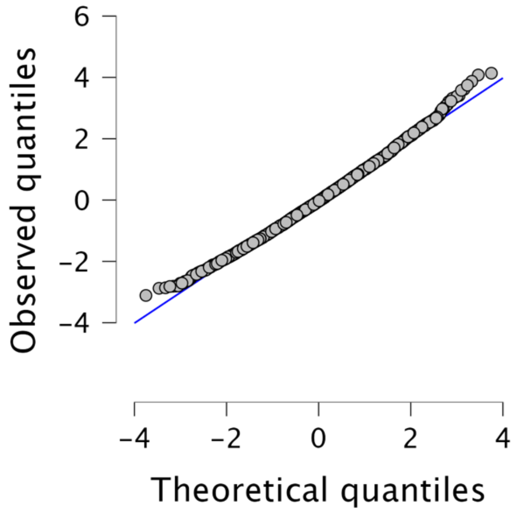

Residuals vs. Fitted Plot

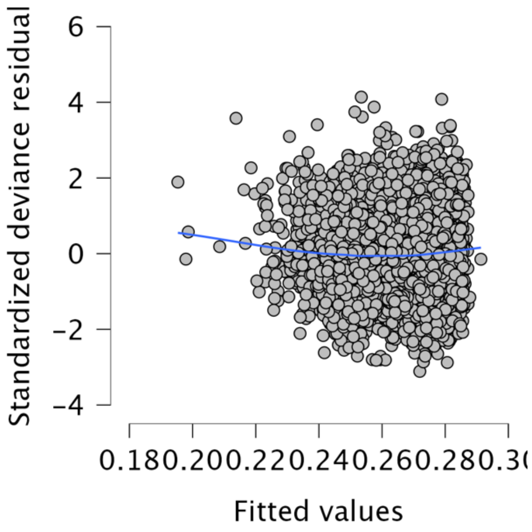

Partial Residual Plots

Partial residual plot for Household & Community Instability

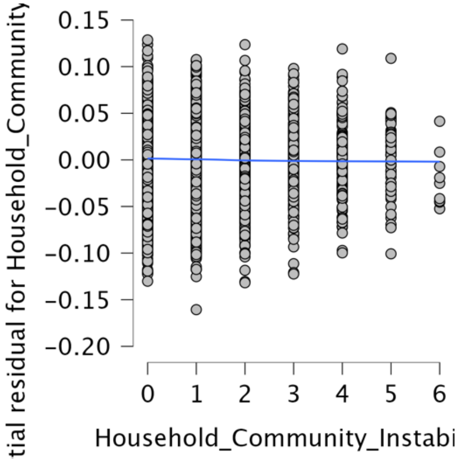

Partial residual plot for Physical & Sexual Abuse

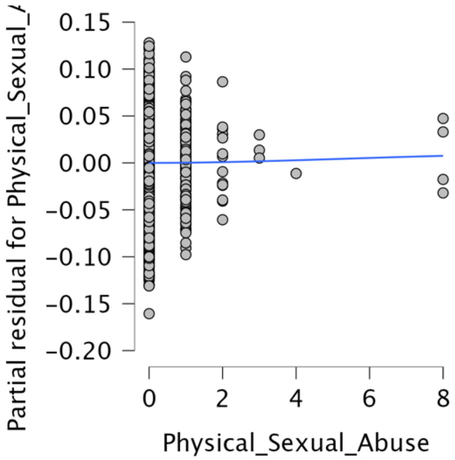

Partial residual plot for Parental Neglect

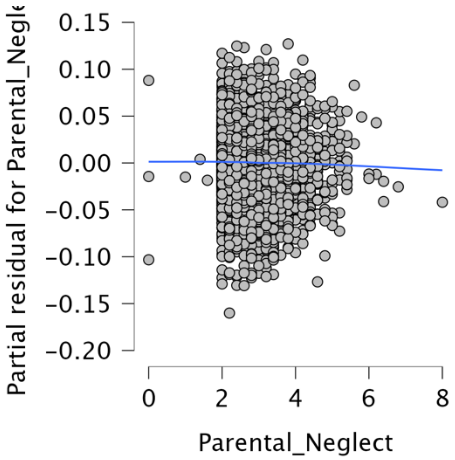

Partial residual plot for Financial_Difficulties

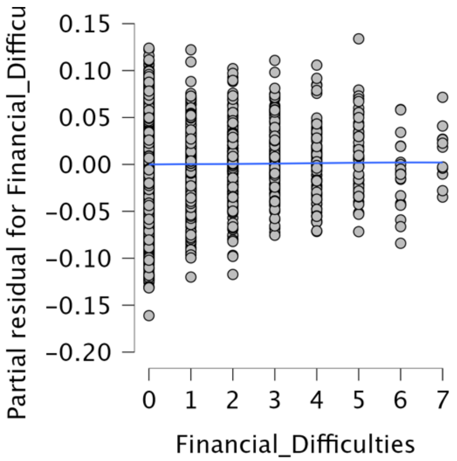

Multicollinearity Diagnostics

	**VIF**
Household & Community Instability	1.156
Physical & Sexual Abuse	1.034
Parental Neglect	1.042
Financial Difficulties	1.179
Age	1.019
Mean head motion	1.034
Sex	1.029
Scanner type	1.038
Race/ethnicity	1.132

Notes. VIF above 4 or below 0.25 indicates that multicollinearity may exist.

1.3.3 Assortativity

Q-Q plot: Standardized deviance residuals

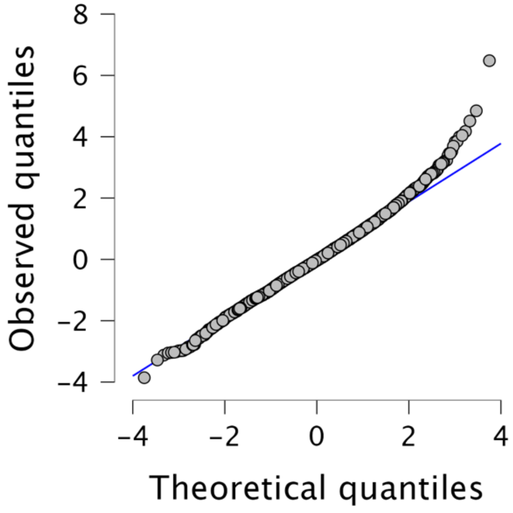

Std deviance residuals vs. fitted values

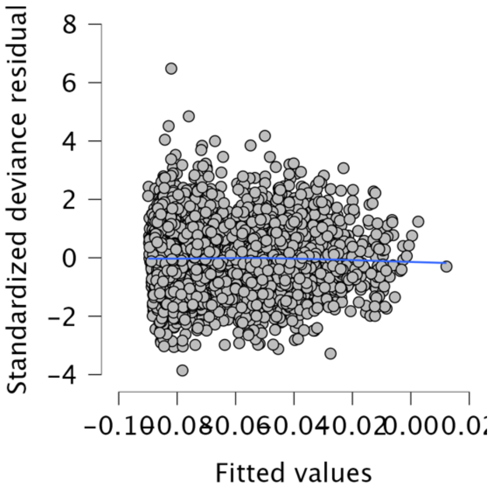

Partial Residual Plots

Partial residual plot for Household & Community Instability

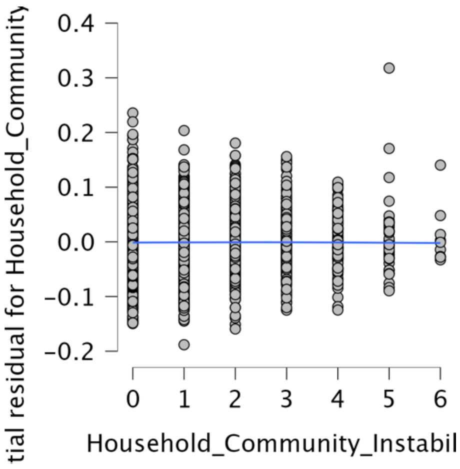

Partial residual plot for Physical & Sexual Abuse

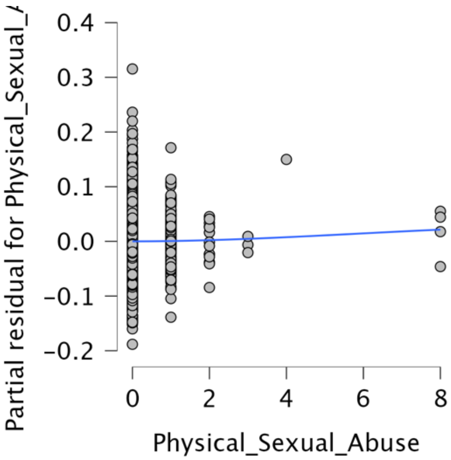

Partial residual plot for Parental Neglect

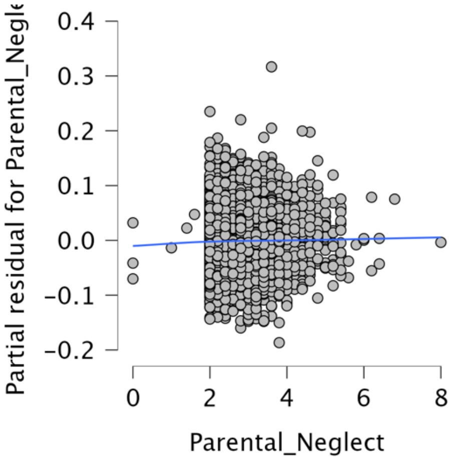

Partial residual plot for Financial Difficulties

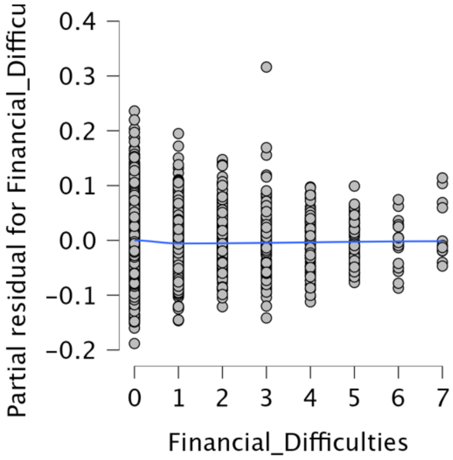

Multicollinearity Diagnostics

	**VIF**
Household & Community Instability	1.156
Physical & Sexual Abuse	1.034
Parental Neglect	1.042
Financial Difficulties	1.179
Age	1.019
Mean head motion	1.034
Sex	1.029
Race/ethnicity	1.132
Scanner type	1.038

Notes. VIF above 4 or below 0.25 indicates that multicollinearity may exist.

2. RESULTS SUPPLEMENT

Table S3. Descriptive statistics for study variables

	Mean	SD	min	max
Adversity				
Household/Community Instability	0.96	1.13	0	6
Physical/Sexual Abuse	0.04	0.29	0	8
Parental Neglect	2.78	0.66	0	8
Financial Difficulties	0.41	1.04	0	7
Cumulative adversity	4.20	0.48	0	17.8
Adversity class (high)	N= 2034			
Mental health				
Internalising	48.1	10.53	33	93
Externalising	45.1	10.03	33	83

Notes. Measures taken at baseline when participants were aged 9-10. Categories of adversity obtained using mixed-graphical models (see main manuscript). The cumulative adversity score was obtained by summing all adversity items, and a binary adversity categorisation derived using a median split of high- and low-adversity groups.

Table S4. ANOVAs comparing adversity levels by race/ethnicity and parental education.

	SS	MS	F	η^2	p
**Cumulative adversity**					
Race/Ethnicity	1447.16	361.79	98.634	0.063	<.001
College-educated caregiver	2782.67	2782.67	614.871	0.121	<.001
**Houshold/Community Instability**					
Race/Ethnicity	185.277	46.319	51.107	0.025	<.001
College-educated caregiver	479.229	479.229	335.955	0.065	<.001
**Physical/Sexual Abuse**					
Race/Ethnicity	0.552	0.138	1.594	0.001	0.173
College-educated caregiver	0.841	0.841	9.217	0.002	0.002
**Parental Neglect**					
Race/Ethnicity	20.081	5.02	9.433	0.008	<.001
College-educated caregiver	19.22	19.22	41.093	0.008	<.001
**Financial Difficulties**					
Race/Ethnicity	406.706	101.676	77.075	0.065	<.001
College-educated caregiver	653.238	653.238	461.993	0.104	<.001

Notes. SS= sum of squares; MS= mean of squares. Type III Sum of Squares. Welch homogeneity correction used. College education reported if one or more caregivers has a college-level degree.

Figure S1. Cumulative adversity by parental education

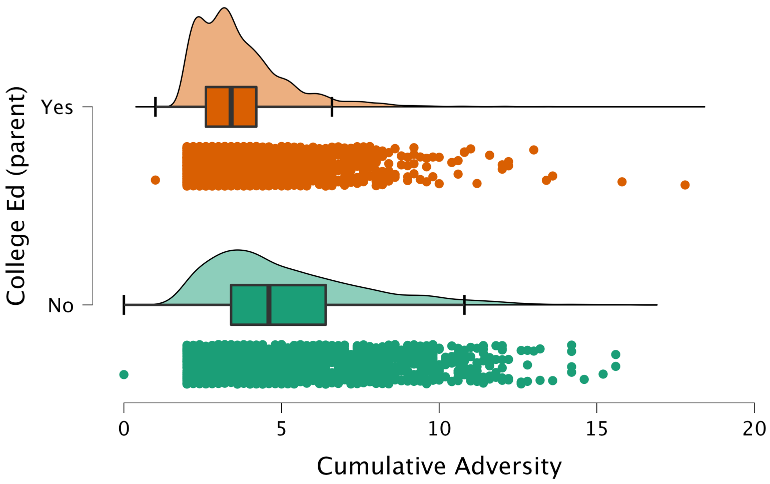

Notes. Statistics shown in Table S4. College education reported if one or more caregivers has a college-level degree.

Table S5. Post-hoc comparisons of adversity by race/ethnicity

		Mean Diff	SE	t	Cohen's d	p_{bonf}
**Cumulative Adversity**						
Asian	Black	-2.197	0.202	-10.902	-1.14	<.001
	Hispanic	-1.304	0.245	-5.329	-0.676	<.001
	Other	-1.337	0.195	-6.863	-0.694	<.001
	White	-0.766	0.191	-4.009	-0.397	<.001
Black	Hispanic	0.894	0.172	5.188	0.464	<.001
	Other	0.86	0.088	9.735	0.446	<.001
	White	1.431	0.08	18	0.743	<.001
Hispanic	Other	-0.034	0.164	-0.205	-0.017	1
	White	0.538	0.16	3.364	0.279	0.008
Other	White	0.571	0.061	9.427	0.296	<.001
**Household/Community Instability**						
Asian	Black	-0.979	0.116	-8.416	-0.88	<.001
	Hispanic	-0.591	0.141	-4.187	-0.531	<.001
	Other	-0.731	0.112	-6.501	-0.657	<.001
	White	-0.518	0.11	-4.698	-0.466	<.001
Black	Hispanic	0.388	0.099	3.9	0.349	<.001
	Other	0.248	0.051	4.861	0.223	<.001
	White	0.461	0.046	10.044	0.414	<.001
Hispanic	Other	-0.14	0.095	-1.475	-0.126	1
	White	0.073	0.092	0.794	0.066	1
Other	White	0.213	0.035	6.093	0.192	<.001
**Physical/Sexual Abuse**						
Asian	Black	-0.051	0.031	-1.646	-0.172	0.998
	Hispanic	-0.007	0.037	-0.176	-0.022	1

	Other	-0.048	0.03	-1.609	-0.163	1
	White	-0.036	0.029	-1.245	-0.123	1
Black	Hispanic	0.044	0.026	1.676	0.15	0.939
	Other	0.003	0.013	0.206	0.009	1
	White	0.014	0.012	1.181	0.049	1
Hispanic	Other	-0.041	0.025	-1.645	-0.14	1
	White	-0.03	0.024	-1.219	-0.101	1
Other	White	0.012	0.009	1.248	0.039	1
**Parental Neglect**						
Asian	Black	-0.225	0.069	-3.285	-0.343	0.01
	Hispanic	-0.071	0.083	-0.856	-0.109	1
	Other	-0.113	0.066	-1.7	-0.172	0.892
	White	-0.05	0.065	-0.765	-0.076	1
Black	Hispanic	0.154	0.059	2.627	0.235	0.086
	Other	0.113	0.03	3.744	0.172	0.002
	White	0.176	0.027	6.488	0.268	<.001
Hispanic	Other	-0.041	0.056	-0.741	-0.063	1
	White	0.022	0.054	0.397	0.033	1
Other	White	0.063	0.021	3.055	0.096	0.023
**Financial Difficulties**						
Asian	Black	-0.942	0.105	-8.964	-0.937	<.001
	Hispanic	-0.634	0.128	-4.973	-0.631	<.001
	Other	-0.445	0.102	-4.383	-0.443	<.001
	White	-0.162	0.1	-1.623	-0.161	1
Black	Hispanic	0.308	0.09	3.426	0.306	0.006
	Other	0.497	0.046	10.785	0.494	<.001
	White	0.78	0.041	18.82	0.776	<.001
Hispanic	Other	0.189	0.086	2.205	0.188	0.275
	White	0.473	0.083	5.671	0.47	<.001

Other	White	0.284	0.032	8.973	0.282	<.001

Notes. Significance value adjusted for comparing a family of 5.

Figure S2. Cumulative adversity by race/ethnicity

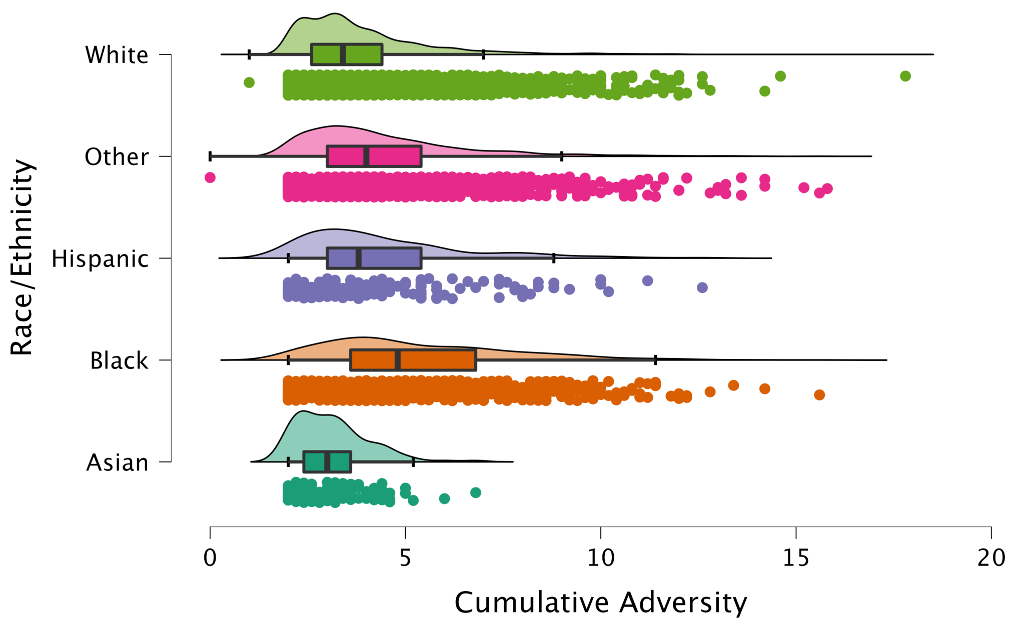

Notes. Statistics shown in Table S5.

Table S6. Centrality indices for the adversity network

Adversity Item	strength	bridge strength	node #
interparental violence	1.090	2.853	10
community shooting or stabbing	0.428	2.257	4
shot, stabbed, beaten by non-family	1.683	1.816	5
severely beaten by caregiver	1.698	0.909	7
financial difficulties: phone service	0.454	0.234	18
parent trouble with job/fights/police	-0.155	0.144	3
unsafe community	-1.044	0.143	14
financial difficulties: food	0.420	0.117	17
sexual abuse by caregiver	0.980	0.109	11
financial difficulties: rent payment	1.030	-0.108	19
financial difficulties: dentist	0.159	-0.287	23
death threat by family	0.389	-0.385	9
parent drug use problem	-1.073	-0.433	2
financial difficulties: medical care	-0.256	-0.441	22
sexual abuse by peer	-0.995	-0.541	13
sudden death of loved one	-1.214	-0.550	24
parent alcohol problem	-0.944	-0.588	1
financial difficulties: gas and electric	-0.114	-0.639	21
poor parental supervision	-1.686	-0.696	15
shot, stabbed, beaten by caregiver	1.171	-0.782	6
death threat by non-family	0.272	-0.782	8
sexual abuse by non-family	0.025	-0.782	12
financial difficulties: eviction	-0.586	-0.782	20
low caregiver acceptance	-1.732	-0.782	16

Notes. Strength reflects the degree to which the node is connected to other nodes in the network. Bridge strength reflects the degree to which the node is connected to other nodes that are not in the same community. Node # refers to structure shown on Figure 2 of the main manuscript.

Figure S3. Centrality indices for the adversity network

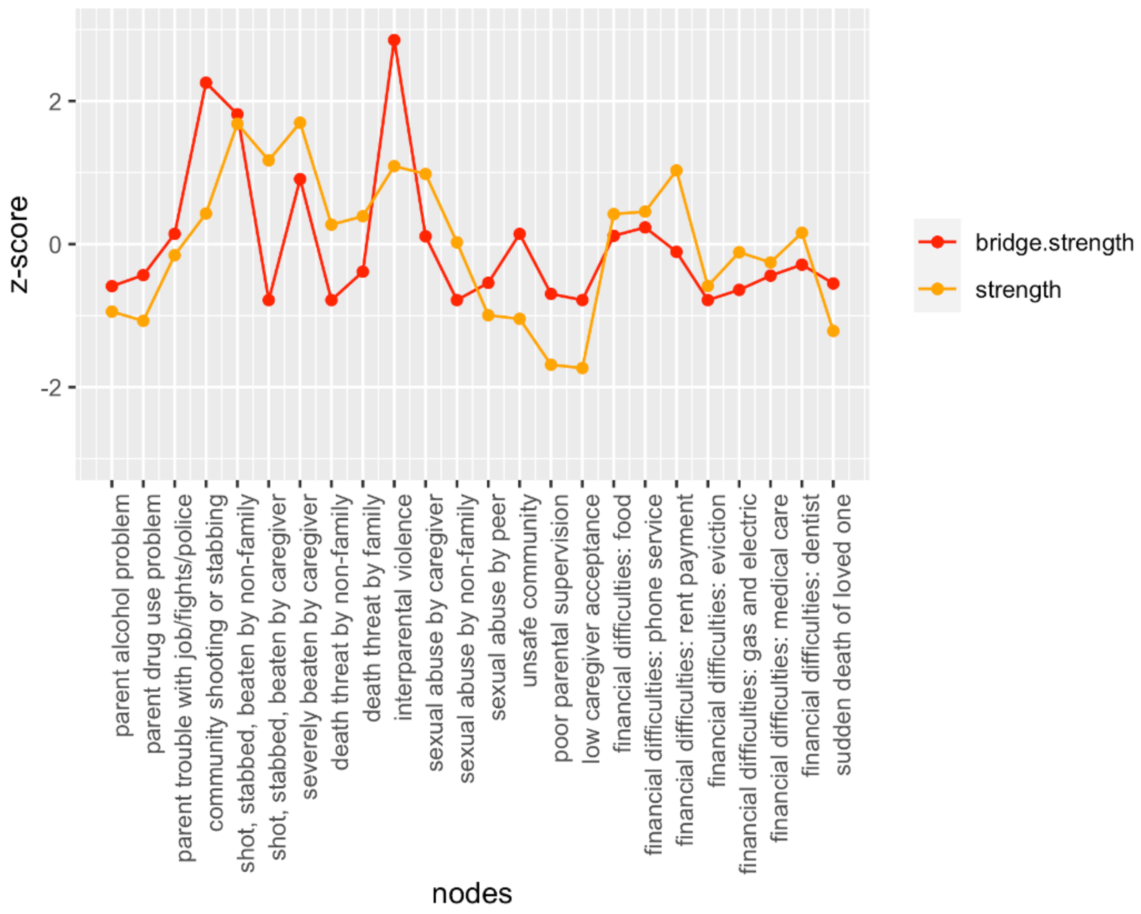

Notes. Strength and bridge strength for each adversity item using z-standardised scores. Higher scores indicate greater centrality.

2.1 Generalized Linear Model

2.1.1 Adversity and global network topology

Table S7. GLM association between categories of adversity and global network measures

	Clustering Coefficient				Modularity				Assortativity			
	β	SE	*t*	*P*	β	SE	*t*	*P*	β	SE	*t*	*P*
	Dev=3.914, X^2=1.058, *P*<.001				*Dev*=7.956, X^2= 1.587, *P*<.001				*Dev*=14.323, X^2=1.657, *P*<.001			
Age	0.000	0.000	4.630	<.001	0.000	0.000	2.334	0.020	0.000	0.000	-0.467	0.640
Sex (M)	-0.007	0.001	-9.649	<.001	-0.013	0.001	-13.002	<.001	-0.002	0.001	-1.559	0.119
MRI mean head motion	-0.053	0.003	-16.675	<.001	-0.085	0.005	-18.541	<.001	0.042	0.006	6.903	<.001
Scanner type (Philips)	-0.003	0.002	-1.058	0.290	-0.018	0.002	-8.984	<.001	0.013	0.003	4.781	<.001
Scanner type (Siemens)	-0.024	0.002	-9.819	<.001	0.018	0.001	14.941	<.001	-0.030	0.002	-18.176	<.001
Household/Community Inst	-0.001	0.000	-2.434	0.015	-0.001	0.000	-1.828	0.068	0.001	0.001	2.358	0.018
Physical/Sexual Abuse	0.002	0.001	1.727	0.084	0.001	0.002	0.709	0.478	0.002	0.002	1.081	0.280
Parental Neglect	-0.001	0.001	-2.225	0.026	-0.001	0.001	-1.407	0.159	0.002	0.001	2.278	0.023
Financial Difficulties	-0.001	0.000	-3.005	0.003	0.001	0.000	1.722	0.085	0.003	0.001	3.886	<.001

Notes. Generalized linear model (GLM) with age, sex, scanner head motion, and scanner type added as covariates. Age reported in years. Scanner type uses GE as reference category.

Table S8. Direct effects of adversity on internalising difficulties at baseline

			Estimate	Std. Error	z-value	p	95% CI Lower	95% CI Upper
Household/community inst	→	Internalising	1.919	0.137	13.966	<.001	1.65	2.189
Physical/sexual abuse	→	Internalising	0.903	0.633	1.427	0.154	-0.338	2.144
Parental neglect	→	Internalising	0.636	0.213	2.995	0.003	0.22	1.053
Financial difficulties	→	Internalising	1.367	0.158	8.626	<.001	1.056	1.677

Notes. Delta method standard errors, normal theory 1000 bootstrap confidence intervals, ML estimator. Controlling for age, sex, MRI head motion and scanner-type.

Table S9. Direct effects of adversity on externalising difficulties at baseline

			Estimate	Std. Error	z-value	p	95% CI Lower	95% CI Upper
Household/community inst	→	Externalising	2.009	0.133	15.068	<.001	1.747	2.27
Physical/sexual abuse	→	Externalising	0.831	0.645	1.288	0.198	-0.434	2.095
Parental neglect	→	Externalising	1.386	0.207	6.691	<.001	0.98	1.792
Financial difficulties	→	Externalising	1.236	0.158	7.809	<.001	0.926	1.547

Notes. Delta method standard errors, normal theory 1000 bootstrap confidence intervals, ML estimator. Controlling for age, sex, MRI head motion and scanner-type.

Table S10. Indirect effects of adversity on internalising difficulties at baseline

					Estimate	Std. Error	z-value	p	95% CI Lower	95% CI Upper
Household/community inst	→	clustering coefficient	→	Internalising	-0.006	0.006	-1.111	0.267	-0.018	0.005
Household/community inst	→	modularity	→	Internalising	-0.002	0.003	-0.539	0.590	-0.008	0.005
Household/community inst	→	assortativity	→	Internalising	-0.01	0.006	-1.567	0.117	-0.022	0.002
Physical/sexual abuse	→	clustering coefficient	→	Internalising	0.02	0.018	1.118	0.264	-0.015	0.054
Physical/sexual abuse	→	modularity	→	Internalising	0.004	0.008	0.482	0.630	-0.012	0.019
Physical/sexual abuse	→	assortativity	→	Internalising	-0.022	0.02	-1.094	0.274	-0.06	0.017
Parental neglect	→	clustering coefficient	→	Internalising	-0.012	0.01	-1.14	0.254	-0.032	0.008
Parental neglect	→	modularity	→	Internalising	-0.003	0.005	-0.536	0.592	-0.013	0.008
Parental neglect	→	assortativity	→	Internalising	-0.022	0.011	-1.919	0.055	-0.043	0.001
Financial difficulties	→	clustering coefficient	→	Internalising	-0.009	0.008	-1.201	0.230	-0.025	0.006
Financial difficulties	→	modularity	→	Internalising	0.003	0.005	0.559	0.576	-0.007	0.013
Financial difficulties	→	assortativity	→	Internalising	-0.02	0.009	-2.39	0.017	-0.037	-0.004

Notes. Delta method standard errors, normal theory 1000 bootstrap confidence intervals, ML estimator. Controlling for age, sex, MRI head motion and scanner-type.

Table S11. Indirect effects of adversity on externalising difficulties at baseline

					Estimate	Std. Error	z-value	p	95% CI Lower	95% CI Upper
Household/community inst	→	clustering coefficient	→	Externalising	-0.003	0.005	-0.659	0.51	-0.012	0.006
Household/community inst	→	modularity	→	Externalising	-0.003	0.003	-0.745	0.457	-0.009	0.004
Household/community inst	→	assortativity	→	Externalising	-0.003	0.003	-0.821	0.411	-0.009	0.004
Physical/sexual abuse	→	clustering coefficient	→	Externalising	0.01	0.014	0.668	0.504	-0.019	0.038
Physical/sexual abuse	→	modularity	→	Externalising	0.006	0.009	0.607	0.544	-0.012	0.023
Physical/sexual abuse	→	assortativity	→	Externalising	-0.006	0.008	-0.716	0.474	-0.021	0.01
Parental neglect	→	clustering coefficient	→	Externalising	-0.006	0.009	-0.669	0.504	-0.023	0.011
Parental neglect	→	modularity	→	Externalising	-0.004	0.006	-0.737	0.461	-0.016	0.007
Parental neglect	→	assortativity	→	Externalising	-0.006	0.007	-0.854	0.393	-0.018	0.007
Financial difficulties	→	clustering coefficient	→	Externalising	-0.005	0.007	-0.684	0.494	-0.018	0.009
Financial difficulties	→	modularity	→	Externalising	0.004	0.005	0.82	0.412	-0.006	0.014
Financial difficulties	→	assortativity	→	Externalising	-0.005	0.006	-0.888	0.374	-0.017	0.006

Notes. Delta method standard errors, normal theory 1000 bootstrap confidence intervals, ML estimator. Controlling for age, sex, MRI head motion and scanner-type.

Table S12. Direct effects of cumulative adversity on concurrent internalising difficulties

			Estimate	Std. Error	z-value	p	95% CI Lower	95% CI Upper
Cumulative adversity	→	Internalising	1.499	0.1	19.756	<.001	1.350	1.648
Cumulative adversity	→	Externalising	1.582	0.1	20.595	<.001	1.431	1.732

Notes. Delta method standard errors, normal theory 1000 bootstrap confidence intervals, ML estimator. Controlling for age, sex, MRI head motion and scanner-type.

Table S13. Indirect effects of cumulative adversity on concurrent internalising difficulties

					Estimate	Std. Error	z-value	p	95% CI Lower	95% CI Upper
Cumulative adversity	→	clustering	→	Internalising	-0.009	0.004	-2.097	0.036	-0.018	-0.001
Cumulative adversity	→	assortativity	→	Internalising	-0.016	0.005	-2.933	0.003	-0.027	-0.005
Cumulative adversity	→	clustering	→	Externalising	-0.007	0.004	-1.692	0.091	-0.014	0.001
Cumulative adversity	→	assortativity	→	Externalising	-0.004	0.005	-0.929	0.353	-0.013	0.005

Notes. Delta method standard errors, normal theory 1000 bootstrap confidence intervals, ML estimator. Controlling for age, sex, MRI head motion and scanner-type.

Table S14. Direct effects of adversity on internalising difficulties three years later

			Estimate	Std. Error	z-value	p	95% CI Lower	95% CI Upper
Household/community inst	→	Internalising	0.702	0.147	4.766	<.001	0.413	0.991
Physical/sexual abuse	→	Internalising	-0.900	0.494	-1.822	0.068	-1.867	0.068
Parental neglect	→	Internalising	0.135	0.24	0.562	0.574	-0.335	0.605
Financial difficulties	→	Internalising	-0.031	0.16	-0.192	0.848	-0.345	0.283

Notes. Delta method standard errors, normal theory 1000 bootstrap confidence intervals, ML estimator. Controlling for age, sex, MRI head motion, scanner-type, and baseline symptoms.

Table S15. Direct effects of adversity on externalising difficulties three years later

			Estimate	Std. Error	z-value	p	95% CI Lower	95% CI Upper
Household/community inst	→	Externalising	0.508	0.126	4.035	<.001	0.261	0.754
Physical/sexual abuse	→	Externalising	-0.73	0.422	-1.727	0.084	-1.558	0.098
Parental neglect	→	Externalising	0.434	0.206	2.107	0.035	0.03	0.838
Financial difficulties	→	Externalising	-0.019	0.137	-0.138	0.891	-0.288	0.25

Notes. Delta method standard errors, normal theory 1000 bootstrap confidence intervals, ML estimator. Controlling for age, sex, MRI head motion, scanner-type, and baseline symptoms.

Table S16. Indirect effects of adversity on internalising difficulties three years later

					Estimate	Std. Error	z-value	p	95% CI Lower	95% CI Upper
Household/community inst	→	modularity	→	Internalising	0.002	0.005	0.306	0.759	-0.008	0.011
Household/community inst	→	clustering coefficient	→	Internalising	0.000	0.002	-0.255	0.799	-0.004	0.003
Household/community inst	→	assortativity	→	Internalising	-0.002	0.004	-0.537	0.592	-0.009	0.005
Physical/sexual abuse	→	modularity	→	Internalising	-0.009	0.018	-0.524	0.601	-0.044	0.025
Physical/sexual abuse	→	clustering coefficient	→	Internalising	0.007	0.02	0.324	0.746	-0.033	0.046
Physical/sexual abuse	→	assortativity	→	Internalising	-0.011	0.014	-0.766	0.444	-0.038	0.017
Parental neglect	→	modularity	→	Internalising	0.006	0.009	0.641	0.521	-0.012	0.023
Parental neglect	→	clustering coefficient	→	Internalising	-0.003	0.008	-0.321	0.748	-0.019	0.014
Parental neglect	→	assortativity	→	Internalising	-0.009	0.009	-0.981	0.326	-0.026	0.009
Financial difficulties	→	modularity	→	Internalising	-0.009	0.009	-1.054	0.292	-0.027	0.008
Financial difficulties	→	clustering coefficient	→	Internalising	-0.003	0.009	-0.327	0.744	-0.02	0.014
Financial difficulties	→	assortativity	→	Internalising	-0.005	0.005	-0.924	0.356	-0.015	0.006

Notes. Delta method standard errors, normal theory 1000 bootstrap confidence intervals, ML estimator. Controlling for age, sex, MRI head motion, scanner-type, and baseline symptoms.

Table S17. Indirect effects of adversity on externalising difficulties three years later

					Estimate	Std. Error	z-value	p	95% CI Lower	95% CI Upper
Household/community inst	→	modularity	→	Externalising	0.001	0.003	0.38	0.704	-0.005	0.007
Household/community inst	→	clustering coefficient	→	Externalising	0.000	0.001	-0.193	0.847	-0.003	0.002
Household/community inst	→	assortativity	→	Externalising	0.000	0.002	0.219	0.827	-0.003	0.004
Physical/sexual abuse	→	modularity	→	Externalising	-0.005	0.011	-0.466	0.641	-0.026	0.016
Physical/sexual abuse	→	clustering coefficient	→	Externalising	0.004	0.017	0.231	0.818	-0.03	0.037
Physical/sexual abuse	→	assortativity	→	Externalising	0.005	0.009	0.562	0.574	-0.013	0.023
Parental neglect	→	modularity	→	Externalising	0.004	0.006	0.60	0.549	-0.008	0.016
Parental neglect	→	clustering coefficient	→	Externalising	-0.002	0.007	-0.23	0.818	-0.016	0.013
Parental neglect	→	assortativity	→	Externalising	0.004	0.006	0.634	0.526	-0.009	0.017
Financial difficulties	→	modularity	→	Externalising	-0.005	0.007	-0.746	0.456	-0.018	0.008
Financial difficulties	→	clustering coefficient	→	Externalising	-0.002	0.007	-0.232	0.817	-0.016	0.013
Financial difficulties	→	assortativity	→	Externalising	0.002	0.004	0.605	0.545	-0.005	0.009

Notes. Delta method standard errors, normal theory 1000 bootstrap confidence intervals, ML estimator. Controlling for age, sex, MRI head motion, scanner-type, and baseline symptoms.

2.1.2 Sensitivity Analyses

Table S18. GLM association between cumulative adversity score and global network measures

	**Clustering Coefficient**				**Modularity**				**Assortativity**			
	β	SE	*t*	*P*	β	SE	*t*	*P*	β	SE	*t*	*P*
	Dev=3.919, X^2=0.916, *P*<.001				*Dev*=7.956, X^2= 1.569, *P*<.001				*Dev*=14.326, X^2=1.653, *P*<.001			
Age	0.000	0.000	4.713	<.001	0.000	0.000	2.444	0.015	0.000	0.000	-0.484	0.629
Sex (M)	-0.007	0.001	-9.856	<.001	-0.013	0.001	-13.267	<.001	-0.002	0.001	-1.514	0.130
MRI mean head motion	-0.054	0.003	-16.711	<.001	-0.085	0.005	-18.552	<.001	0.043	0.006	6.939	<.001
Scanner type (Philips)	0.000	0.001	-0.313	0.754	-0.018	0.002	-8.976	<.001	0.013	0.003	4.795	<.001
Scanner type (Siemens)	0.022	0.001	25.516	<.001	0.018	0.001	14.952	<.001	-0.030	0.002	-18.163	<.001
Cumulative adversity	-0.001	0.000	-4.707	<.001	0.000	0.000	-0.497	0.619	0.034	0.005	7.166	<.001

Notes. Generalized linear model (GLM) with age, sex, scanner head motion, and scanner type added as covariates. Age reported in years. Scanner type uses GE as reference category. Cumulative adversity score calculated by summing the total number of questions endorsed.

Table S19. GLM association between binary adversity class and global network measures

	**Clustering Coefficient**				**Modularity**				**Assortativity**			
	β	SE	*t*	*P*	β	SE	*t*	*P*	β	SE	*t*	*P*
	Dev=3.92, X^2=0.913, *P*<.001				*Dev*=7.965, X^2=1.570, *P*<.001				*Dev*=14.348, X^2=1.628, *P*<.001			
Age	0.000	0.000	4.703	<.001	0.000	0.000	2.427	0.015	0.000	0.000	-0.482	0.630
Sex (M)	-0.007	0.001	-9.889	<.001	-0.013	0.001	-13.258	<.001	-0.002	0.001	-1.459	0.145

	β	SE	*t*	*P*	β	SE	*t*	*P*	β	SE	*t*	*P*
MRI mean head motion	-0.054	0.003	-16.816	<.001	-0.085	0.005	-18.548	<.001	0.043	0.006	7.084	<.001
Scanner type (Philips)	0.000	0.001	-0.253	0.800	-0.018	0.002	-8.995	<.001	0.012	0.003	4.690	<.001
Scanner type (Siemens)	0.022	0.001	25.595	<.001	0.018	0.001	14.955	<.001	-0.030	0.002	-18.269	<.001
Adversity class (High)	-0.003	0.001	-4.384	<.001	-0.001	0.001	-1.007	0.314	0.008	0.001	5.449	<.001

Notes. Generalized linear model (GLM) with age, sex, scanner head motion, and scanner type added as covariates. Age reported in years. Scanner type uses GE as reference category. Adversity class defined using a median split of high and low adversity based on the total number of questions endorsed.

Table S20. GLM association between categories of adversity and topological properties of network null models.

	**Clustering Coefficient**				**Modularity**				**Assortativity**			
	β	SE	*t*	*P*	β	SE	*t*	*P*	β	SE	*t*	*P*
	Dev=2.155, X^2=0.306, *P*<.001				*Dev*=0.086, X^2= 0.009, *P*<.001				*Dev*=0.938, X^2=0.47, *P*<.001			
Age	0.000	0.000	4.268	<.001	0.000	0.000	1.138	0.255	0.000	0.000	1.225	0.220
Sex (M)	-0.004	0.001	-7.283	<.001	0.000	0.000	-3.225	0.001	-0.001	0.000	-3.868	<.001
MRI mean head motion	-0.020	0.002	-8.372	<.001	-0.005	0.000	-11.254	<.001	-0.010	0.002	-6.305	<.001
Scanner type (Philips)	0.005	0.001	5.200	<.001	-0.004	0.000	-19.993	<.001	-0.010	0.001	-13.897	<.001
Scanner type (Siemens)	0.015	0.001	23.085	<.001	-0.001	0.000	-6.870	<.001	-0.004	0.000	-9.186	<.001
Household/Community Inst	0.000	0.000	-1.956	0.051	0.000	0.000	-0.350	0.727	0.000	0.000	-0.386	0.699
Physical/Sexual Abuse	0.001	0.001	1.519	0.129	0.000	0.000	0.551	0.582	0.000	0.001	0.236	0.814
Parental Neglect	-0.001	0.000	-2.980	0.003	0.000	0.000	-0.522	0.602	0.000	0.000	-0.097	0.923
Financial Difficulties	-0.001	0.000	-3.215	0.001	0.000	0.000	2.830	0.005	0.001	0.000	3.437	<.001

Notes. Values for graph measures obtained by comparing actual network measures to those of a randomised network that preserves the degree, weight, and strength distributions. Age reported in years. Scanner type uses GE as reference category.

Table S21. GLM associations between categories of adversity and global network measures thresholded at 90%

	Clustering Coefficient				Modularity				Assortativity			
	β	SE	*t*	*P*	β	SE	*t*	*P*	β	SE	*t*	*P*
	Dev=6.604, X^2=1.443, *P*<.001				*Dev*=3.058, X^2=0.461, *P*<.001				*Dev*=21.199, X^2=0.256, *P*<.001			
Age	0.000	0.000	4.324	<.001	0.000	0.000	1.505	0.132	0.000	0.000	0.110	0.912
Sex (M)	-0.009	0.001	-9.494	<.001	-0.004	0.001	-6.016	<.001	0.006	0.002	3.529	<.001
MRI mean head motion	-0.079	0.004	-18.552	<.001	-0.049	0.003	-17.003	<.001	-0.018	0.008	-2.397	0.017
Scanner type (Philips)	0.002	0.002	1.095	0.274	-0.005	0.001	-4.154	<.001	0.012	0.003	3.840	<.001
Scanner type (Siemens)	0.026	0.001	23.186	<.001	0.013	0.001	17.025	<.001	0.011	0.002	5.277	<.001
Household/Community Inst	-0.001	0.000	-2.522	0.012	0.000	0.000	-1.222	0.222	0.001	0.001	1.509	0.131
Physical/Sexual Abuse	0.002	0.002	1.517	0.129	0.000	0.001	-0.042	0.966	0.011	0.003	3.966	<.001
Parental Neglect	-0.001	0.001	-2.013	0.044	0.000	0.000	-0.200	0.841	0.001	0.001	0.627	0.531
Financial Difficulties	-0.001	0.000	-3.005	0.003	0.000	0.000	-0.208	0.835	0.000	0.001	0.205	0.838

Notes. Results for thresholded matrices retaining top 10% of connection weights. Age reported in years. Scanner type uses GE as reference category.

Table S22. GLM associations between categories of adversity and global network measures thresholded at 80%

	Clustering Coefficient				Modularity				Assortativity			
	β	SE	*t*	*P*	β	SE	*t*	*P*	β	SE	*t*	*P*
**Model**	*Dev*=5.085, X^2=1.072, *P*<.001				*Dev*=3.741, X^2=0.717, *P*<.001				*Dev*=17.560, X^2=0.685, *P*<.001			
Age	0.000	0.000	4.367	<.001	0.000	0.000	1.506	0.132	0.000	0.000	-1.683	0.092
Sex (M)	-0.008	0.001	-9.598	<.001	-0.007	0.001	-9.874	<.001	0.005	0.002	3.349	0.001
MRI mean head motion	-0.063	0.004	-17.026	<.001	-0.062	0.003	-19.393	<.001	0.028	0.007	4.062	<.001

	β	SE	t	P	β	SE	t	P	β	SE	t	P
Scanner type (Philips)	0.004	0.002	2.642	0.008	-0.007	0.001	-5.284	<.001	0.028	0.003	9.574	<.001
Scanner type (Siemens)	0.024	0.001	24.078	<.001	0.015	0.001	17.651	<.001	-0.005	0.002	-2.471	0.014
Household/Community Inst	-0.001	0.000	-2.416	0.016	0.000	0.000	-1.462	0.144	0.002	0.001	2.726	0.006
Physical/Sexual Abuse	0.002	0.001	1.455	0.146	0.000	0.001	-0.031	0.975	0.006	0.003	2.282	0.023
Parental Neglect	-0.001	0.001	-2.226	0.026	0.000	0.001	-0.399	0.690	0.003	0.001	2.404	0.016
Financial Difficulties	-0.001	0.000	-2.807	0.005	0.000	0.000	0.374	0.709	0.001	0.001	1.009	0.313

Notes. Results for thresholded matrices retaining top 20% of connection weights. Age reported in years. Scanner type uses GE as reference category.

Table S23. GLM associations between categories of adversity and global network measures thresholded at 70%

	**Clustering Coefficient**				**Modularity**				**Assortativity**			
	β	SE	*t*	*P*	β	SE	*t*	*P*	β	SE	*t*	*P*
	Dev=4.012, X^2=0.849, *P*<.001				*Dev*=3.786, X^2=0.738, *P*<.001				*Dev*=16.461, X^2=1.203, *P*<.001			
Age	0.000	0.000	4.400	<.001	0.000	0.000	2.103	0.036	0.000	0.000	-2.513	0.012
Sex (M)	-0.007	0.001	-9.318	<.001	-0.008	0.001	-11.400	<.001	0.005	0.001	3.743	<.001
MRI mean head motion	-0.054	0.003	-16.259	<.001	-0.063	0.003	-19.528	<.001	0.053	0.007	7.941	<.001
Scanner type (Philips)	0.004	0.001	3.009	0.003	-0.008	0.001	-5.981	<.001	0.036	0.003	12.690	<.001
Scanner type (Siemens)	0.022	0.001	24.820	<.001	0.014	0.001	16.669	<.001	-0.007	0.002	-3.803	<.001
Household/Community Inst	-0.001	0.000	-2.311	0.021	0.000	0.000	-1.427	0.154	0.002	0.001	2.751	0.006
Physical/Sexual Abuse	0.002	0.001	1.551	0.121	0.000	0.001	0.350	0.726	0.003	0.002	1.350	0.177
Parental Neglect	-0.001	0.001	-2.347	0.019	0.000	0.001	-0.388	0.698	0.003	0.001	2.715	0.007
Financial Difficulties	-0.001	0.000	-2.868	0.004	0.000	0.000	0.351	0.726	0.000	0.001	0.537	0.591

Notes. Results for thresholded matrices retaining top 30% of connection weights. Age reported in years. Scanner type uses GE as reference category.

Table S24. GLM associations between categories of adversity and global network measures thresholded at 60%

	Clustering Coefficient				Modularity				Assortativity			
	β	SE	*t*	*P*	β	SE	*t*	*P*	β	SE	*t*	*P*
	Dev=3.471, X^2=0.743. *P*<.001				*Dev*=3.789, X^2=0.771, *P*<.001				*Dev*=15.033, X^2=1.322, *P*<.001			
Age	0.000	0.000	4.438	<.001	-0.001	0.000	-2.117	0.034	0.000	0.000	-3.071	0.002
Sex (M)	-0.006	0.001	-9.308	<.001	0.057	0.005	11.906	<.001	0.006	0.001	4.466	<.001
MRI mean head motion	-0.050	0.003	-16.213	<.001	0.479	0.024	19.963	<.001	0.063	0.006	9.841	<.001
Scanner type (Philips)	0.004	0.001	2.735	0.006	0.075	0.010	7.301	<.001	0.039	0.003	14.069	<.001
Scanner type (Siemens)	0.020	0.001	24.967	<.001	-0.090	0.006	-15.035	<.001	-0.005	0.002	-3.231	0.001
Household/Community Inst	-0.001	0.000	-2.268	0.023	0.004	0.002	1.692	0.091	0.002	0.001	2.690	0.007
Physical/Sexual Abuse	0.002	0.001	1.619	0.105	-0.003	0.008	-0.375	0.708	0.001	0.002	0.459	0.647
Parental Neglect	-0.001	0.001	-2.389	0.017	0.001	0.004	0.269	0.788	0.003	0.001	2.630	0.009
Financial Difficulties	-0.001	0.000	-2.837	0.005	-0.002	0.002	-0.882	0.378	0.000	0.001	-0.122	0.903

Notes. Results for thresholded matrices retaining top 40% of connection weights. Age reported in years. Scanner type uses GE as reference category.

Table S25. GLM associations between categories of adversity and global network measures from matrices thresholded at 0

	Clustering Coefficient				Modularity				Assortativity			
	β	SE	*t*	*P*	β	SE	*t*	*P*	β	SE	*t*	*P*
	Dev=3.355, X^2=0.741, *P*<.001				*Dev*=4.078, X^2=0.866, *P*<.001				*Dev*=12.075, X^2=0.941, *P*<.001			
Age	0.000	0.000	4.457	<.001	0.000	0.000	2.062	0.039	0.000	0.000	-3.273	0.001
Sex (M)	-0.006	0.001	-9.601	<.001	-0.009	0.001	-12.195	<.001	0.005	0.001	4.216	<.001
MRI mean head motion	-0.051	0.003	-16.760	<.001	-0.069	0.003	-20.780	<.001	0.054	0.006	9.487	<.001

Scanner type (Philips)	0.002	0.001	1.759	0.079	-0.013	0.001	-9.278	<.001	0.032	0.002	12.948	<.001
Scanner type (Siemens)	0.020	0.001	24.776	<.001	0.013	0.001	14.549	<.001	-0.005	0.002	-3.112	0.002
Household/Community Inst	-0.001	0.000	-2.272	0.023	0.000	0.000	-1.422	0.155	0.002	0.001	2.718	0.007
Physical/Sexual Abuse	0.002	0.001	1.648	0.099	0.001	0.001	0.458	0.647	0.000	0.002	0.108	0.914
Parental Neglect	-0.001	0.001	-2.403	0.016	0.000	0.001	-0.376	0.707	0.002	0.001	2.546	0.011
Financial Difficulties	-0.001	0.000	-2.712	0.007	0.000	0.000	1.162	0.245	0.000	0.001	-0.088	0.930

Notes. GLM results for thresholded matrices retaining only positive weights. Age reported in years. Scanner type uses GE as reference category.

Table S26. GLM associations between categories of adversity and overall functional connectivity

	**Average edge weight**			
	β	SE	*t*	*P*
	Dev=0.171 X^2=0.035 *P*<.001			
Age	0.000	0.000	-1.323	0.186
Sex (M)	0.001	0.000	6.586	<.001
MRI mean head motion	0.006	0.001	8.999	<.001
Scanner type (Philips)	0.009	0.000	30.567	<.001
Scanner type (Siemens)	0.002	0.000	9.724	<.001
Household/Community Instability	0.000	0.000	0.819	0.413
Physical/Sexual Abuse	0.000	0.000	0.126	0.899
Parental Neglect	0.000	0.000	1.578	0.115
Financial Difficulties	0.000	0.000	-3.521	<.001

Notes. Age reported in years. Scanner type uses GE as reference category.

2.1.3 Adversity and network-level topology

Table S27. GLM associations between categories of adversity and network-level measures

	Clustering Coefficient				Assortativity			
Network	β	SE	*t*	P_{FDR}	β	SE	*t*	P_{FDR}
**Auditory**	Dev=6.174 X^2=0.580 P<.001				Dev=2.310 X^2=0.120 P<.001			
Household/Community Instability	0.000	0.000	-0.404	0.686	0.000	0.000	1.581	0.212
Physical/Sexual Abuse	0.000	0.002	0.316	0.815	-0.001	0.001	-0.729	0.757
Parental Neglect	0.000	0.001	-0.371	0.711	0.000	0.000	0.538	0.698
Financial Difficulties	-0.001	0.000	-1.992	0.100	0.000	0.000	-0.143	0.887
**Cingulo-Opercular**	Dev=5.145 X^2=0.815 P<.001				Dev=1.168 X^2=0.067 P<.001			
Household/Community Instability	-0.001	0.000	-1.882	0.111	0.000	0.000	-1.470	0.231
Physical/Sexual Abuse	0.001	0.001	0.813	0.601	0.001	0.001	1.557	0.520
Parental Neglect	-0.001	0.001	-2.203	0.061	0.000	0.000	-1.682	0.302
Financial Difficulties	-0.001	0.000	-3.383	0.002	0.000	0.000	0.481	0.746
**Cingulo-Parietal**	Dev=4.308 X^2=0.252 P<.001				Dev=0.133 X^2=0.002 P<.001			
Household/Community Instability	-0.001	0.000	-2.132	0.072	0.000	0.000	2.168	0.098
Physical/Sexual Abuse	0.001	0.001	0.854	0.639	0.000	0.000	-0.549	0.758
Parental Neglect	-0.001	0.001	-1.691	0.131	0.000	0.000	0.811	0.602
Financial Difficulties	0.000	0.000	-0.882	0.410	0.000	0.000	3.444	0.004
**Dorsal Attention**	Dev=3.639 X^2=0.543 P<.001				Dev=0.540 X^2=0.018 P<.001			
Household/Community Instability	-0.001	0.000	-2.660	0.026	0.000	0.000	-1.843	0.141
Physical/Sexual Abuse	0.002	0.001	1.499	0.348	0.001	0.000	1.662	0.631
Parental Neglect	-0.001	0.001	-2.656	0.035	0.000	0.000	-0.434	0.664
Financial Difficulties	-0.001	0.000	-1.724	0.123	0.000	0.000	2.560	0.024
**Default**	Dev=5.499 X^2=1.038 P<.001				Dev=0.787 X^2=0.017 P<.001			
Household/Community Instability	-0.001	0.000	-2.891	0.050	0.000	0.000	-0.297	0.767
Physical/Sexual Abuse	0.003	0.001	1.915	0.364	0.000	0.001	0.325	0.880

Parental Neglect	-0.002	0.001	-2.345	0.049	0.000	0.000	1.305	0.499
Financial Difficulties	-0.001	0.000	-1.756	0.128	0.000	0.000	0.544	0.763
**Fronto-Parietal**	*Dev*=4.212 X^2=0.531 *P*<.001				*Dev*=0.498 X^2=0.017 *P*<.001			
Household/Community Instability	-0.001	0.000	-1.608	0.156	0.000	0.000	0.337	0.797
Physical/Sexual Abuse	0.002	0.001	1.816	0.301	0.001	0.000	2.234	0.325
Parental Neglect	-0.001	0.001	-2.046	0.076	0.000	0.000	0.628	0.689
Financial Difficulties	-0.001	0.000	-1.584	0.147	0.000	0.000	3.149	0.009
**Retrosplenial-Temporal**	*Dev*=6.385 X^2=0.955 *P*<.001				*Dev*=0.257 X^2=0.011 *P*<.001			
Household/Community Instability	-0.001	0.000	-2.833	0.022	0.000	0.000	-4.546	0.000
Physical/Sexual Abuse	0.001	0.002	0.563	0.678	0.000	0.000	1.240	0.699
Parental Neglect	-0.002	0.001	-3.333	0.011	0.000	0.000	-3.122	0.026
Financial Difficulties	-0.001	0.000	-1.770	0.143	0.000	0.000	-1.308	0.310
**Salience**	*Dev*=3.943 X^2=0.455 *P*<.001				*Dev*=0.130 X^2=0.002 *P*<.001			
Household/Community Instability	0.000	0.000	-1.421	0.202	0.000	0.000	2.639	0.035
Physical/Sexual Abuse	0.002	0.001	1.448	0.321	0.000	0.000	-0.711	0.689
Parental Neglect	0.000	0.001	-0.834	0.477	0.000	0.000	1.112	0.576
Financial Difficulties	-0.001	0.000	-3.387	0.003	0.000	0.000	2.566	0.026
**Sensorimotor Hand**	*Dev*=6.809 X^2=0.360 *P*<.001				*Dev*=6.265 X^2=0.508 *P*<.001			
Household/Community Instability	0.001	0.000	1.174	0.284	0.002	0.000	3.765	0.001
Physical/Sexual Abuse	0.000	0.002	0.286	0.775	-0.001	0.002	-0.963	0.728
Parental Neglect	0.001	0.001	0.764	0.482	0.001	0.001	2.018	0.191
Financial Difficulties	0.001	0.000	2.488	0.034	0.003	0.000	6.220	0.000
**Sensorimotor Mouth**	*Dev*=7.606 X^2=0.767 *P*<.001				*Dev*=0.530 X^2=0.024 *P*<.001			
Household/Community Instability	0.000	0.000	-0.634	0.570	0.000	0.000	2.058	0.104
Physical/Sexual Abuse	0.001	0.002	0.787	0.560	0.000	0.000	-0.777	0.812
Parental Neglect	-0.001	0.001	-1.862	0.102	0.000	0.000	0.908	0.592
Financial Difficulties	0.000	0.001	-0.586	0.558	0.000	0.000	3.010	0.010
**Ventral Attention**	*Dev*=4.461 X^2=0.732 *P*<.001				*Dev*=0.900 X^2=0.003 *P*=0.066			
Household/Community Instability	-0.001	0.000	-2.327	0.052	0.000	0.000	1.049	0.425

Physical/Sexual Abuse	0.002	0.001	1.942	0.676	0.001	0.001	0.968	0.866
Parental Neglect	-0.001	0.001	-0.952	0.443	0.001	0.000	2.161	0.202
Financial Difficulties	0.000	0.000	-1.283	0.235	0.000	0.000	0.152	0.953
**Visual**	*Dev*=32.296 X^2=4.824 *P*<.001				*Dev*=1.816 X^2=0.025 *P*<.001			
Household/Community Instability	-0.002	0.001	-1.852	0.104	0.000	0.000	-0.392	0.821
Physical/Sexual Abuse	0.005	0.003	1.570	0.377	0.000	0.001	0.305	0.823
Parental Neglect	-0.004	0.002	-2.414	0.052	0.000	0.000	1.002	0.587
Financial Difficulties	-0.004	0.001	-4.293	0.000	0.000	0.000	0.911	0.524
**Subcortical**	*Dev*=2.848 X^2=0.606 *P*<.001				*Dev*=2.581 X^2=0.137 *P*<.001			
Household/Community Instability	-0.001	0.000	-2.824	0.031	0.000	0.000	0.720	0.614
Physical/Sexual Abuse	0.001	0.001	1.202	0.426	0.000	0.001	-0.126	0.900
Parental Neglect	-0.001	0.000	-2.806	0.033	0.000	0.000	0.521	0.652
Financial Difficulties	-0.001	0.000	-4.358	0.000	0.000	0.000	-1.503	0.247

Notes. Functional networks defined using the Gordon atlas. Age, sex, scanner type, and scanner head motion were included as covariates in the model but are not reported here for the sake of brevity. P-values corrected for the number of networks (n=13) using false-discovery rate (FDR; q<0.05).

2.2 Partial Least Squares

Table 28. VIP scores for adversity items on global PLS

Adversity item	VIP	Coeff	Std. err	t	p	2.50%	97.50%
poor parental supervision	**1.676**	**-0.010**	**0.004**	**-2.23**	**0.026**	**-0.018**	**-0.001**
unsafe community	**1.644**	**-0.010**	**0.004**	**-2.46**	**0.014**	**-0.017**	**-0.003**
financial difficulties: rent payment	**1.609**	**-0.009**	**0.003**	**-3.09**	**0.002**	**-0.015**	**0.001**
financial difficulties: phone service	**1.548**	**-0.009**	**0.003**	**-2.80**	**0.005**	**-0.015**	**0.001**

financial difficulties: food	**1.458**	**-0.009**	**0.003**	**-2.92**	**0.004**	**-0.014**	**0.001**
sudden death of loved one	**1.333**	**-0.008**	**0.003**	**-2.33**	**0.020**	**-0.014**	**-0.003**
parent trouble with job/fights/police	**1.266**	**-0.007**	**0.003**	**-2.64**	**0.008**	**-0.013**	**-0.001**
financial difficulties: gas and electric	**1.214**	**-0.007**	**0.003**	**-2.76**	**0.006**	**-0.012**	**0.008**
financial difficulties: eviction	**1.103**	**-0.006**	**0.002**	**-2.66**	**0.008**	**-0.011**	**0.008**
low caregiver acceptance	**1.029**	**-0.006**	**0.003**	**-1.96**	**0.050**	**-0.012**	**0.000**
interparental violence	0.955	-0.006	0.003	-2.10	0.036	-0.011	0.000
financial difficulties: dentist	0.938	-0.005	0.002	-2.37	0.018	-0.010	0.005
sexual abuse by peer	0.680	-0.004	0.003	-1.49	0.136	-0.009	-0.002
parent alcohol problem	0.589	-0.003	0.002	-1.73	0.083	-0.007	0.000
community shooting or stabbing	0.583	-0.003	0.002	-1.67	0.096	-0.007	0.003
financial difficulties: medical care	0.553	-0.003	0.002	-1.75	0.081	-0.007	0.001
shot, stabbed, beaten by caregiver	0.540	-0.003	0.002	-1.62	0.105	-0.007	-0.002
death threat by family	0.529	0.003	0.002	1.28	0.202	-0.002	0.004
severely beaten by caregiver	0.446	0.003	0.003	1.01	0.311	-0.002	-0.001
shot, stabbed, beaten by non-family	0.334	-0.002	0.002	-1.14	0.256	-0.005	-0.003
sexual abuse by caregiver	0.224	0.001	0.002	0.76	0.450	-0.002	-0.002
parent drug use problem	0.222	0.001	0.002	0.65	0.516	-0.003	0.000
sexual abuse by non-family	0.121	0.001	0.002	0.37	0.711	-0.003	-0.002
death threat by non-family	0.086	-0.001	0.002	-0.27	0.784	-0.004	0.005

Notes. Variable importance in projection (VIP) was used to assess the relative importance of each adversity item in the PLS model. Bold indicates VIP scores considered most influential in terms of their explanatory power (>1). The stability of each item was assessed using cross-validation (nrepeat= 10, folds= 10). The Jack-Knife approach was used to obtain regression coefficients, confidence intervals, and p-values were obtained for each adversity item.

Table S29. Direct effects of PLS predictor score on mental health symptoms three years later

			Estimate	Std. Error	z-value	p	95% CI Lower	95% CI Upper
PLSpred	→	Internalising	0.109	0.102	1.07	0.285	-0.091	0.31
PLSpred	→	Externalising	0.152	0.088	1.723	0.085	-0.021	0.324

Notes. PLSpred= PLS predictor scores. Delta method standard errors, normal theory 1000 bootstrap confidence intervals, ML estimator. Controlling for age, sex, MRI head motion, scanner-type, and baseline symptoms.

Table S30. Indirect effects of PLS predictor score on mental health symptoms three years later

					Estimate	Std. Error	z-value	p	95% CI Lower	95% CI Upper
PLSpred	→	PLSresp	→	Internalising	0.001	0.01	0.086	0.931	-0.019	0.02
PLSpred	→	PLSresp	→	Externalising	0.009	0.008	1.043	0.297	-0.008	0.025

Notes. PLSpred= PLS predictor scores representing the latent score derived from adversity items; PLSresp= PLS response scores representing the latent score derived from global network measures. Delta method standard errors, normal theory 1000 bootstrap confidence intervals, ML estimator. Controlling for age, sex, MRI head motion, scanner-type, and baseline symptoms.

Table S31. Network-level PLS loadings

Network-level measure	Loading
clustcoef_Subcortical	-0.321
clustcoef_Visual	-0.309
clustcoef_COP	-0.284
clustcoef_DAN	-0.245
clustcoef_RSPT	-0.244
clustcoef_Default	-0.244
clustcoef_Salience	-0.232
assort_RSPT	-0.230
clustcoef_FPN	-0.199
clustcoef_VAN	-0.192
clustcoef_CPAR	-0.162
clustcoef_AUD	-0.154
assort_COP	-0.103
clustcoef_SMM	-0.098
assort_DAN	-0.024
assort_Visual	-0.022
assort_Default	-0.021
assort_Subcortical	0.027
assort_VAN	0.050
assort_FPN	0.079
assort_AUD	0.092
clustcoef_SMH	0.105
assort_Salience	0.142
assort_CPAR	0.192
assort_SMM	0.207
assort_SMH	0.397

Notes. Network-level clustering coefficient (clustcoef) and assortativity (assort) loadings for the PLS.

Table 32. VIP scores for adversity items on network-level PLS

Adversity item	VIP	Coeff	Std. err	t	p	2.50%	97.50%
unsafe community	**1.821**	**-0.014**	**0.0036**	**-3.91**	**< 0.001**	**-0.021**	**-0.007**
financial difficulties: phone service	**1.754**	**-0.014**	**0.0032**	**-4.28**	**< 0.001**	**-0.020**	**-0.007**
poor parental supervision	**1.634**	**-0.013**	**0.0035**	**-3.58**	**< 0.001**	**-0.020**	**-0.006**
financial difficulties: rent payment	**1.598**	**-0.012**	**0.0029**	**-4.31**	**< 0.001**	**-0.018**	**-0.007**
parent trouble with job/fights/police	**1.442**	**-0.011**	**0.0027**	**-4.08**	**< 0.001**	**-0.016**	**-0.006**
financial difficulties: gas and electric	**1.312**	**-0.010**	**0.0026**	**-3.82**	**< 0.001**	**-0.015**	**-0.005**
financial difficulties: food	**1.312**	**-0.010**	**0.0028**	**-3.66**	**< 0.001**	**-0.016**	**-0.005**
financial difficulties: dentist	**1.140**	**-0.009**	**0.0027**	**-3.28**	**0.001**	**-0.014**	**-0.004**
sudden death of loved one	**1.086**	**-0.008**	**0.0026**	**-3.16**	**0.002**	**-0.014**	**-0.003**
interparental violence	**1.068**	**-0.008**	**0.0025**	**-3.32**	**0.001**	**-0.013**	**-0.003**
financial difficulties: eviction	0.919	-0.007	0.0024	-3.01	0.003	-0.012	-0.002
low caregiver acceptance	0.807	-0.006	0.0024	-2.59	0.010	-0.011	-0.002
financial difficulties: medical care	0.596	-0.005	0.0022	-2.13	0.033	-0.009	0.000
community shooting or stabbing	0.590	-0.005	0.0021	-2.20	0.028	-0.009	0.000
death threat by family	0.541	0.004	0.0023	1.78	0.074	0.000	0.009
severely beaten by caregiver	0.450	0.003	0.0026	1.34	0.181	-0.002	0.009
sexual abuse by peer	0.357	-0.003	0.0023	-1.17	0.241	-0.007	0.002
sexual abuse by caregiver	0.355	0.003	0.0019	1.45	0.146	-0.001	0.006
parent alcohol problem	0.348	-0.003	0.002	-1.33	0.185	-0.007	0.001
shot, stabbed, beaten by caregiver	0.296	-0.002	0.0016	-1.38	0.166	-0.006	0.001
sexual abuse by non-family	0.200	0.002	0.0019	0.81	0.416	-0.002	0.005
death threat by non-family	0.196	-0.002	0.0019	-0.81	0.419	-0.005	0.002

shot, stabbed, beaten by non-family	0.079	-0.001	0.0017	-0.36	0.717	-0.004	0.003
parent drug use problem	0.062	0.000	0.002	0.24	0.808	-0.003	0.004

Notes. Variable importance in projection (VIP) was used to assess the relative importance of each adversity item in the PLS model. Bold indicates VIP scores considered most influential in terms of their explanatory power (>1). The stability of each item was assessed using cross-validation (nrepeat= 10, folds= 10). The Jack-Knife approach was used to obtain regression coefficients, confidence intervals, and p-values were obtained for each adversity item.

2.3 **Supplemental analyses controlling for race/ethnicity and parental education**

A set of supplemental analyses were conducted that additionally controlled for race/ethnicity and parental education, as these are commonly added as covariates in studies of adversity and brain organisation (e.g., Tooley et al., 2020; Kim et al., 2019; Gellci et al., 2019; Ohashi et al., 2017). We report these results for transparency, although we focus primarily on uncorrected results in the main text for two reasons: (1) as we have shown in the Results section, race/ethnicity and parental education are highly covaried with adversity in the ABCD sample, and (2) from a statistical perspective, it is not possible to accurately address the question of whether individuals who differ on race/ethnicity or parental education would still differ on their exposure to adversity if they did not differ on these demographic characteristics. In other words, statistically controlling for pre-existing group differences that are inherently related to adversity will remove some or all adversity-related variance, producing biased estimates and spurious results (*Lord's Paradox*; Lord, 1969; Miller & Chapman 2001). We therefore caution the reader when interpreting the below results, which are reported for comparison and to highlight the importance of analytical decisions with the growing availability of large representative samples that vary in their demographic composition.

2.3.1 Generalized Linear Model

Adversity and global network topology. We conducted a set of GLM analyses to determine whether categories of adversity were associated with variation in global clustering coefficient, modularity, and assortativity. Age, sex, parental education, race/ethnicity, scanner head motion, scanner type, and all four categories of adversity were included in the same model as covariates (Table S31). Greater financial difficulties were associated with significantly greater modularity (β= .001, p= .006). There were no significant associations of any other category of adversity with the three global network measures.

Table S33. GLM association between categories of adversity and global network measures controlling for race/ethnicity and parental education

	**Clustering Coefficient**				**Modularity**				**Assortativity**			
	β	SE	*t*	*P*	β	SE	*t*	*P*	β	SE	*t*	*P*
	Dev=3.689 X^2=1.064 *P*<.001				*Dev*=7.739 X^2=1.640 *P*<.001				*Dev*=13.437 X^2=2.102 *P*<.001			
Age	0.000	0.000	4.861	<.001	0.000	0.000	2.449	0.014	0.000	0.000	-1.060	0.289
Sex (M)	-0.007	0.001	-10.589	<.001	-0.013	0.001	-13.190	<.001	-0.002	0.001	-1.307	0.191
Race/Ethnicity (Asian)	0.007	0.003	2.460	0.014	0.000	0.004	0.019	0.985	-0.021	0.005	-3.951	<.001
Race/Ethnicity (Hispanic)	0.015	0.002	6.489	<.001	0.012	0.003	3.448	<.001	-0.023	0.004	-5.148	<.001
Race/Ethnicity (White)	0.015	0.001	12.988	<.001	0.009	0.002	5.093	<.001	-0.026	0.002	-11.679	<.001
Race/Ethnicity (Other)	0.011	0.001	9.406	<.001	0.010	0.002	5.416	<.001	-0.015	0.002	-6.535	<.001
College education	0.002	0.001	2.678	0.007	0.002	0.001	1.643	0.101	-0.003	0.002	-2.199	0.028
MRI mean head motion	-0.048	0.003	-14.955	<.001	-0.083	0.005	-17.818	<.001	0.031	0.006	5.125	<.001
Scanner type (Philips)	-0.001	0.001	-0.472	0.637	-0.018	0.002	-8.853	<.001	0.013	0.003	5.109	<.001
Scanner type (Siemens)	0.022	0.001	25.773	<.001	0.019	0.001	15.028	<.001	-0.030	0.002	-18.269	<.001
Household/Community Inst	0.000	0.000	-1.122	0.262	-0.001	0.000	-1.392	0.164	0.001	0.001	0.920	0.358
Physical/Sexual Abuse	0.002	0.001	1.383	0.167	0.001	0.002	0.583	0.560	0.003	0.002	1.406	0.160
Parental Neglect	-0.001	0.001	-1.275	0.202	-0.001	0.001	-0.966	0.334	0.001	0.001	1.490	0.136
Financial Difficulties	0.000	0.000	0.426	0.670	0.002	0.001	2.927	0.003	0.000	0.001	0.459	0.646

Notes. Generalized linear model (GLM) with age, sex, race/ethnicity, parental education, scanner head motion, and scanner type added as covariates. Age reported in years. Race/ethnicity uses African American as the reference category. College education reported as true if one or more caregivers has a college level degree. Scanner type uses GE as reference category.

Mediating effects of global modularity on mental health outcomes. We tested for possible mediating effects of modularity on the relationship between financial difficulties and mental health. Greater financial difficulties were associated with significantly greater concurrent internalising (β= 2.075, p<.001) and externalising difficulties (β= 1.806, p<.001) controlling for age, sex, parental education, race/ethnicity, scanner motion and scanner type. However, the mediating effect of modularity on the association between financial difficulties and internalising (β= .009, p= .124) and externalising difficulties (β= .005, p= .239) was non-significant. After controlling for baseline symptoms, financial difficulties did not predict greater internalising (β= 0.293, p=.069) nor externalising (β= 0.181, p= .19) difficulties 3 years later, and there were no mediating effects of modularity (*p's*= .247- .853).

Sensitivity Analyses. To test the utility of using data-driven categorisations of adversity, we ran two supplemental GLMs using (1) a cumulative adversity score and (2) a binary split of high- and low-adversity groups instead of the four adversity categories. We found a small but significant association of cumulative adversity with assortativity (β= .001, p= .026). There were no significant associations with modularity or clustering coefficient in the cumulative and the case-control adversity models (Tables S32-S33). Thus, the results did not align with the main GLM findings described above, indicating possible bias induced by controlling for race/ethnicity and parental education due to their covariance with adversity in ABCD.

Table S34. GLM association between cumulative adversity score and global network measures controlling for race/ethnicity and parental education.

	Clustering Coefficient				Modularity				Assortativity			
	β	SE	*t*	*P*	β	SE	*t*	*P*	β	SE	*t*	*P*
	Dev=3.70 X^2=1.062 *P*<.001				*Dev*=7.752 X^2=1.628 *P*<.001				*Dev*=013.442 X^2=2.097 *P*<.001			
Age	0.000	0.000	4.965	<.001	0.000	0.000	2.559	0.011	0.000	0.000	-1.111	0.267
Sex (M)	-0.007	0.001	-10.831	<.001	-0.013	0.001	-13.447	<.001	-0.002	0.001	-1.214	0.225
Race/Ethnicity (Asian)	0.007	0.003	2.474	0.013	0.000	0.004	0.007	0.995	-0.021	0.005	-3.924	<.001
Race/Ethnicity (Hispanic)	0.015	0.002	6.505	<.001	0.012	0.003	3.465	<.001	-0.023	0.004	-5.152	<.001
Race/Ethnicity (White)	0.015	0.001	13.039	<.001	0.008	0.002	4.928	<.001	-0.026	0.002	-11.675	<.001
Race/Ethnicity (Other)	0.011	0.001	9.429	<.001	0.009	0.002	5.290	<.001	-0.015	0.002	-6.508	<.001
College education	0.002	0.001	2.664	0.008	0.002	0.001	1.471	0.141	-0.003	0.002	-2.082	0.037
MRI mean head motion	-0.048	0.003	-14.986	<.001	-0.083	0.005	-17.865	<.001	0.032	0.006	5.176	<.001
Scanner type (Philips)	-0.001	0.001	-0.459	0.646	-0.017	0.002	-8.829	<.001	0.013	0.003	5.120	<.001
Scanner type (Siemens)	0.022	0.001	25.812	<.001	0.019	0.001	15.043	<.001	-0.030	0.002	-18.241	<.001
Cumulative adversity	0.000	0.000	-0.637	0.524	0.000	0.000	0.691	0.489	0.001	0.000	2.251	0.024

Notes. Generalized linear model (GLM) with age, sex, race/ethnicity, parental education, scanner head motion, and scanner type added as covariates. Age reported in years. Race/ethnicity uses Black as the reference category. College education reported as true if one or more caregivers has a college level degree. Scanner type uses GE as reference category. Cumulative adversity score calculated by summing the total number of questions endorsed.

Table S35. GLM association between binary adversity class and global network measures controlling for race/ethnicity and parental education.

	Clustering Coefficient				Modularity				Assortativity			
	β	SE	*t*	*P*	β	SE	*t*	*P*	β	SE	*t*	*P*
**Model**	*Dev*=3.70 X^2=1.062 *P*<.001				*Dev*=7.752 X^2=1.627 *P*<.001				*Dev*=13.446 X^2=2.093 *P*<.001			
Age	0.000	0.000	4.957	<.001	0.000	0.000	2.545	0.011	0.000	0.000	-1.114	0.265
Sex (M)	-0.007	0.001	-10.833	<.001	-0.013	0.001	-13.424	<.001	-0.002	0.001	-1.181	0.238
Race/Ethnicity (Asian)	0.007	0.003	2.470	0.014	0.000	0.004	-0.051	0.959	-0.021	0.005	-3.991	<.001
Race/Ethnicity (Hispanic)	0.015	0.002	6.512	<.001	0.011	0.003	3.415	<.001	-0.023	0.004	-5.228	<.001
Race/Ethnicity (White)	0.015	0.001	13.039	<.001	0.008	0.002	4.857	<.001	-0.026	0.002	-11.762	<.001
Race/Ethnicity (Other)	0.011	0.001	9.445	<.001	0.009	0.002	5.243	<.001	-0.015	0.002	-6.593	<.001
College education	0.002	0.001	2.709	0.007	0.002	0.001	1.316	0.188	-0.004	0.002	-2.357	0.018
MRI mean head motion	-0.048	0.003	-14.993	<.001	-0.083	0.005	-17.849	<.001	0.032	0.006	5.209	<.001
Scanner type (Philips)	-0.001	0.001	-0.461	0.645	-0.018	0.002	-8.864	<.001	0.013	0.003	5.082	<.001
Scanner type (Siemens)	0.022	0.001	25.833	<.001	0.018	0.001	15.018	<.001	-0.030	0.002	-18.301	<.001
Adversity class (High)	-0.001	0.001	-0.799	0.424	0.000	0.001	0.086	0.931	0.002	0.001	1.561	0.119

Notes. Generalized linear model (GLM) with age, sex, race/ethnicity, parental education, scanner head motion, and scanner type added as covariates. Age reported in years. Race/ethnicity uses Black as the reference category. College education reported as true if one or more caregivers has a college level degree. Scanner type uses GE as reference category. Adversity class defined using a median split of high and low adversity based on the total number of questions endorsed.

Table S36. GLM association between categories of adversity and topological properties of network null models controlling for race/ethnicity and parental education

	Clustering Coefficient				Modularity				Assortativity			
	β	SE	*t*	*P*	β	SE	*t*	*P*	β	SE	*t*	*P*
	Dev=2.085 X^2=0.376 *P*<.001				*Dev*=0.085 X^2=0.009 *P*<.001				*Dev*=0.932 X^2=0.054 *P*<.001			
Age	0.000	0.000	4.511	<.001	0.000	0.000	1.154	0.248	0.000	0.000	1.218	0.223
Sex (M)	-0.004	0.001	-8.109	<.001	0.000	0.000	-3.137	0.002	-0.001	0.000	-3.599	<.001
Race/Ethnicity (Asian)	0.005	0.002	2.555	0.011	-0.001	0.000	-1.583	0.114	-0.001	0.001	-0.792	0.428
Race/Ethnicity (Hispanic)	0.010	0.002	5.827	<.001	0.000	0.000	-0.327	0.743	-0.001	0.001	-0.758	0.449
Race/Ethnicity (White)	0.011	0.001	12.576	<.001	0.000	0.000	-2.226	0.026	-0.003	0.001	-4.702	<.001
Race/Ethnicity (Other)	0.008	0.001	8.846	<.001	0.000	0.000	-0.323	0.747	-0.001	0.001	-1.216	0.224
College education	0.001	0.001	1.133	0.257	0.000	0.000	1.020	0.308	0.000	0.000	-0.264	0.792
MRI mean head motion	-0.016	0.002	-6.813	<.001	-0.006	0.000	-11.399	<.001	-0.011	0.002	-6.951	<.001
Scanner type (Philips)	0.005	0.001	5.144	<.001	-0.004	0.000	-19.801	<.001	-0.009	0.001	-13.507	<.001
Scanner type (Siemens)	0.015	0.001	23.287	<.001	-0.001	0.000	-6.754	<.001	-0.004	0.000	-8.786	<.001
Household/Community Inst	0.000	0.000	-1.075	0.282	0.000	0.000	-0.418	0.676	0.000	0.000	-0.751	0.453
Physical/Sexual Abuse	0.001	0.001	1.279	0.201	0.000	0.000	0.580	0.562	0.000	0.001	0.348	0.728
Parental Neglect	-0.001	0.000	-1.994	0.046	0.000	0.000	-0.653	0.514	0.000	0.000	-0.499	0.618
Financial Difficulties	0.000	0.000	-0.287	0.774	0.000	0.000	2.405	0.016	0.000	0.000	2.088	0.037

Notes. Values are obtained by comparing actual network measures to those of a randomised network that preserves the degree, weight, and strength distributions. Age reported in years. Race/Ethnicity uses Black as the reference category. College education reported as true if one or more caregivers has a college level degree. Scanner type uses GE as reference category. The network measures obtained for the empirical networks were significantly different from those expected by chance (p's< .001). Financial difficulties were associated with modularity in the randomised network (β= .0001, p= .016), suggesting that differences in network strength were contributing to the association between financial difficulties and modularity.

Adversity and network-level topology. To test whether the effects of adversity varied across functional networks, we repeated our GLMs using the local clustering coefficient and assortativity measure for each 13 Gordon networks. One significant association emerged when race/ethnicity and parental education were included in the model: Greater household and community instability was associated with significantly less assortativity in the retrosplenial-temporal cortex (RSPT; β= -.00003, P_{FDR}= .005). There were no significant associations of adversity with the clustering coefficient computed at each Gordon network (Figure S35).

Table S37. GLM associations between categories of adversity and network-level measures controlling for race/ethnicity and parental education

	Clustering Coefficient				Assortativity			
Network	β	SE	*t*	P_{FDR}	β	SE	*t*	P_{FDR}
**Auditory**	*Dev*=6.115 X^2=0.638 *P*<.001				*Dev*=2.295 X^2=0.135 *P*<.001			
Household/Community Instability	0.000	0.000	0.089	0.929	0.000	0.000	1.037	0.557
Physical/Sexual Abuse	0.000	0.001	0.223	0.824	-0.001	0.001	-0.610	1.174
Parental Neglect	0.000	0.001	0.130	0.971	0.000	0.000	0.172	0.936
Financial Difficulties	0.000	0.000	-0.645	0.675	0.000	0.000	-1.471	0.229
**Cingulo-Opercular**	*Dev*=4.944 X^2=1.016 *P*<.001				*Dev*=1.161 X^2=0.075 *P*<.001			
Household/Community Instability	0.000	0.000	-0.696	0.791	0.000	0.000	-0.979	0.531
Physical/Sexual Abuse	0.001	0.001	0.526	0.779	0.001	0.001	1.448	0.641
Parental Neglect	-0.001	0.001	-1.062	0.535	0.000	0.000	-1.242	0.696
Financial Difficulties	0.000	0.000	0.100	0.997	0.000	0.000	1.684	0.239
**Cingulo-Parietal**	*Dev*=4.211 X^2=0.349 *P*<.001				*Dev*=0.131 X^2=0.003 *P*<.001			
Household/Community Instability	0.000	0.000	-1.359	0.452	0.000	0.000	1.350	0.384
Physical/Sexual Abuse	0.001	0.001	0.651	0.744	0.000	0.000	-0.364	0.931
Parental Neglect	0.000	0.001	-0.851	0.571	0.000	0.000	0.194	1.000
Financial Difficulties	0.001	0.000	1.461	1.872	0.000	0.000	1.303	0.277

**Dorsal Attention**	*Dev*=3.53 X^2=0.652 *P*<.001				*Dev*=0.538 X^2=0.020 *P*<.001			
Household/Community Instability	-0.001	0.000	-1.723	0.368	0.000	0.000	-2.168	0.195
Physical/Sexual Abuse	0.001	0.001	1.265	0.670	0.001	0.000	1.698	0.585
Parental Neglect	-0.001	0.001	-1.684	0.399	0.000	0.000	-0.741	0.9945
Financial Difficulties	0.000	0.000	1.156	1.075	0.000	0.000	1.728	0.364
**Default**	*Dev*=5.360 X^2=1.178 *P*<.001				*Dev*=0.287 X^2=0.019 *P*<.001			
Household/Community Instability	-0.001	0.000	-1.857	0.410	0.000	0.000	-0.091	1.004
Physical/Sexual Abuse	0.002	0.001	1.683	0.598	0.000	0.001	0.304	0.899
Parental Neglect	-0.001	0.001	-1.420	0.406	0.000	0.000	1.438	0.654
Financial Difficulties	0.000	0.000	1.087	0.900	0.000	0.000	0.980	0.425
**Fronto-Parietal**	*Dev*=3.480 X^2=0.654 *P*<.001				*Dev*=0.494 X^2=0.021 *P*<.001			
Household/Community Instability	0.000	0.000	-0.730	0.864	0.000	0.000	-0.041	0.967
Physical/Sexual Abuse	0.002	0.001	1.577	0.498	0.001	0.000	2.344	0.247
Parental Neglect	-0.001	0.001	-1.338	0.392	0.000	0.000	0.220	1.193
Financial Difficulties	0.000	0.000	0.689	0.709	0.000	0.000	1.945	0.338
**Retrosplenial-Temporal**	*Dev*=6.21 X^2=0.13 *P*<.001				*Dev*=0.249 X^2=0.019 *P*<.001			
Household/Community Instability	-0.001	0.000	-2.164	0.390	0.000	0.000	-3.528	0.005
Physical/Sexual Abuse	0.000	0.002	0.324	0.808	0.000	0.000	0.943	0.900
Parental Neglect	-0.002	0.001	-2.463	0.182	0.000	0.000	-2.171	0.390
Financial Difficulties	0.000	0.000	0.733	0.752	0.000	0.000	1.725	0.276
**Salience**	*Dev*=3.860 X^2=0.538 *P*<.001				*Dev*=0.121 X^2=0.004 *P*<.001			
Household/Community Instability	0.000	0.000	-0.539	0.767	0.000	0.000	1.709	0.286
Physical/Sexual Abuse	0.001	0.001	1.236	0.468	0.000	0.000	-0.533	0.858
Parental Neglect	0.000	0.001	0.006	0.995	0.000	0.000	0.523	0.977
Financial Difficulties	0.000	0.000	-0.741	0.852	0.000	0.000	0.506	0.724
**Sensorimotor Hand**	*Dev*=6.76 X^2=0.409 *P*<.001				*Dev*=5.905 X^2=0.869 *P*<.001			
Household/Community Instability	0.000	0.000	0.396	0.818	0.001	0.000	1.947	0.225
Physical/Sexual Abuse	0.001	0.002	0.424	0.793	-0.001	0.001	-0.589	1.033
Parental Neglect	0.000	0.001	0.305	0.988	0.000	0.001	0.584	1.038

Financial Difficulties	0.000	0.000	0.807	1.092	0.001	0.000	1.518	0.240
**Sensorimotor Mouth**	*Dev*=7.574 X^2=0.799 *P*<.001				*Dev*=0.522 X^2=0.032 *P*<.001			
Household/Community Instability	0.000	0.000	-0.602	0.790	0.000	0.000	0.918	0.519
Physical/Sexual Abuse	0.001	0.002	0.755	0.731	0.000	0.000	-0.588	0.905
Parental Neglect	-0.001	0.001	-1.630	0.335	0.000	0.000	0.145	0.885
Financial Difficulties	0.000	0.001	-0.166	1.026	0.000	0.000	0.393	0.752
**Ventral Attention**	*Dev*=4.384 X^2=0.809 *P*<.001				*Dev*=0.895 X^2=0.007 *P*<.001			
Household/Community Instability	0.000	0.000	-1.242	0.464	0.000	0.000	1.354	0.458
Physical/Sexual Abuse	0.002	0.001	1.757	1.027	0.001	0.001	0.973	1.073
Parental Neglect	0.000	0.001	-0.140	1.051	0.001	0.000	2.067	0.254
Financial Difficulties	0.000	0.000	1.240	1.398	0.000	0.000	0.319	0.750
**Visual**	*Dev*=30.497 X^2=6.623 *P*<.001				*Dev*=1.806 X^2=0.034 *P*<.001			
Household/Community Instability	0.000	0.001	-0.360	0.779	0.000	0.000	-0.527	0.777
Physical/Sexual Abuse	0.004	0.003	1.244	0.554	0.000	0.001	0.167	0.939
Parental Neglect	-0.002	0.002	-1.056	0.473	0.000	0.000	1.196	0.603
Financial Difficulties	0.000	0.001	-0.008	0.994	0.000	0.000	1.527	0.275
**Subcortical**	*Dev*=2.681 X^2=0.606 *P*<.001				*Dev*=2.572 X^2=0.147 *P*<.001			
Household/Community Instability	0.000	0.000	-1.584	0.367	0.000	0.000	0.451	0.771
Physical/Sexual Abuse	0.001	0.001	0.829	0.756	0.000	0.001	-0.053	0.957
Parental Neglect	-0.001	0.000	-1.828	0.442	0.000	0.000	0.213	1.080
Financial Difficulties	0.000	0.000	-0.759	0.971	-0.001	0.000	-2.290	0.286

Notes. Functional networks defined using the Gordon atlas. Age, sex, race/ethnicity, parental college education, scanner type, and scanner head motion were included as covariates in the model but are not reported here for the sake of brevity. P-values corrected for the number of networks (n=13) using false-discovery rate (FDR; $q<0.05$).

2.3.2 Partial Least Squares

The correlation between predictor and response scores in the global PLS after adding race/ethnicity and parental education as additional covariates was non-significant (r= 0.02, p= 0.07). The correlation between predictor and response scores in the regional PLS after adding race/ethnicity and parental education as additional covariates was r= 0.05, p= 0.01. These results highlight that including race/ethnicity and parental education washes out adversity-related effects, even when statistical models are designed to optimise for covariance between adversity and brain network measures.

Table S38. Indirect effects of adversity categories on concurrent mental health using raw scores

					**Est**	**Std. Err**	**Lower**	**Upper**	**p**
Household_Community_Inst	→	clustering	→	INT	0.00	0.00	-0.01	0.00	0.27
Household_Community_Inst	→	modularity	→	INT	0.00	0.00	0.00	0.00	0.92
Household_Community_Inst	→	assortativity	→	INT	0.00	0.00	-0.01	0.00	0.14
Physical_Sexual_Abuse	→	clustering	→	INT	0.01	0.01	-0.01	0.03	0.29
Physical_Sexual_Abuse	→	modularity	→	INT	0.00	0.00	-0.01	0.01	0.92
Physical_Sexual_Abuse	→	assortativity	→	INT	-0.01	0.01	-0.03	0.01	0.33
Parental_Neglect	→	clustering	→	INT	-0.01	0.01	-0.02	0.00	0.25
Parental_Neglect	→	modularity	→	INT	0.00	0.00	-0.01	0.01	0.92
Parental_Neglect	→	assortativity	→	INT	-0.01	0.01	-0.02	0.00	0.08
Financial_Difficulties	→	clustering	→	INT	-0.01	0.00	-0.01	0.00	0.23
Financial_Difficulties	→	modularity	→	INT	0.00	0.00	-0.01	0.01	0.92
**Financial_Difficulties**	→	**assortativity**	→	INT	**-0.01**	**0.00**	**-0.02**	**0.00**	**0.04**
Household_Community_Inst	→	clustering	→	EXT	0.00	0.00	-0.01	0.00	0.47
Household_Community_Inst	→	modularity	→	EXT	0.00	0.00	0.00	0.00	0.99

Household_Community_Inst	→	assortativity	→	EXT	0.00	0.00	0.00	0.00	0.84
Physical_Sexual_Abuse	→	clustering	→	EXT	0.01	0.01	-0.01	0.02	0.48
Physical_Sexual_Abuse	→	modularity	→	EXT	0.00	0.00	-0.01	0.01	0.99
Physical_Sexual_Abuse	→	assortativity	→	EXT	0.00	0.00	-0.01	0.01	0.84
Parental_Neglect	→	clustering	→	EXT	0.00	0.01	-0.01	0.01	0.46
Parental_Neglect	→	modularity	→	EXT	0.00	0.00	-0.01	0.01	0.99
Parental_Neglect	→	assortativity	→	EXT	0.00	0.00	-0.01	0.01	0.84
Financial_Difficulties	→	clustering	→	EXT	0.00	0.00	-0.01	0.01	0.45
Financial_Difficulties	→	modularity	→	EXT	0.00	0.00	-0.01	0.01	0.99
Financial_Difficulties	→	assortativity	→	EXT	0.00	0.00	-0.01	0.01	0.84

Notes. INT= internalising raw score; EXT= externalising raw score. Delta method standard errors, normal theory 1000 bootstrap confidence intervals, ML estimator. Controlling for age, sex, MRI head motion and scanner-type.
